# Supplementary material for: Training the Trainer: Preparing Anesthesiology Residents to be Trainers in the Operating Room
Source: MedEdPORTAL. 2021 Mar 4;17:11116. doi: 10.15766/mep_2374-8265.11116 (PMC7970634; doi:10.15766/mep_2374-8265.11116)
Supplement: Supplementary file 1 — Primer Document.docxWorkshop Handout.docxWorkshop PowerPoint.pptxInstructor Manual.docxPresurvey.pdfPostsurvey.pdf1-Week Follow-up Survey.docx1-Month Follow-up Survey.docxNew CA 1 Survey.docx [file mep_2374-8265.11116-s001.zip › A. Primer Document.docx]

Leverage Learning Theory to Become a Better Trainer

**Goal:**

- Equip residents with the knowledge and skills to be an effective trainer in July.

**Objectives:**

- Discuss cognitive load theory as it applies to training-in.
- Describe general principles of effective teaching.
- Evaluate the quality of teaching using a standardized framework.
- Learn how to use microskills for clinical teaching.

**Objective #1: Discuss cognitive load theory as it applies to training-in:**

Cognitive load theory originated in the 1950s with the finding that short-term memory in humans is limited to 7 ± 2 units of information.^1^ Subsequent work suggested that achievement of mastery in a subject involves “chunking” to organize information in short-term memory, also known as **schema** construction.^2^ Muscle memory is an example of schema construction. Think of schemas as the cognitive framework which allows you to develop **automaticity**: “the ability to do things without occupying the mind with the low-level details.”^3^ Automaticity is vital to reducing cognitive load, allowing the brain to focus on higher-level matters. Consider bag mask ventilation: a novice thinks about the placement of each individual finger as well as the amount of pressure to exert in each location. The experienced provider manipulates their hand into the appropriate configuration automatically, allowing them to focus their attention on higher-level tasks such as monitoring the vital signs, assessing lung compliance during bag ventilation, etc.

There are 3 types of cognitive load: intrinsic, extraneous, and germane.

**Intrinsic** = inherent difficulty of the task. For example, placing a central line has a higher intrinsic cognitive load compared to placing a peripheral IV.

**Extraneous** = difficulty created by how information is delivered to the learner. For example, if you were trying to describe a square to someone else, using words alone generates more extraneous cognitive load compared to showing a picture of the square.

**Germane** = processing, construction, and automation of schema. This is the key work that the learner’s brain is performing in order to progress towards mastery.

Our goal as mentors is to **minimize extraneous cognitive load** to allow learners to **maximize germane cognitive load**. With respect to procedural learning, we are letting our learners ingrain patterns that they can carry out automatically with minimal cognitive effort. With this in mind, it is clear that training-in is a crucial period of time, as bad habits that develop during this time are very difficult to unlearn and correct.

How can we decrease extraneous cognitive load?^4^

**Minimize distractions:** Prevent the learner from losing focus on their primary task. This is where our actions can have a huge impact. Maximize the signal:noise ratio. Delegate tasks to keep things simple. For example, when your mentee is initially learning how to mask ventilate, tell them to focus solely on masking while you take care of everything else (i.e. watching the blood pressure and heart rate, watching the volatile anesthetic level, obtaining vascular access, etc).

**Scaffolding:** Provide assistance while the learner is performing a task then gradually withdraw the assistance until the learner is performing the task independently. In a broader sense, this describes residency training. However, this concept can also be applied on an individual level when teaching procedures. For example, you will start out the training-in period doing most of the OR setup in the morning, and gradually peel back until your learner is setting up the entire OR on their own.

**Worked examples:**^5^ Breaking down a complex task into discrete simple steps. Organizing the task into simpler sub-tasks will reduce the cognitive load for the learner. For example, when you are teaching IV placement, break down the process into smaller steps (e.g. identifying landmarks, prepping the skin, getting the flash, and threading the catheter). This makes it easier for the learner to follow along and target their troubleshooting to a specific step.

**Integration:** Auditory and visual data are processed separately by the brain. If you can align the verbal explanation with what the learner is seeing, it will be more likely to stick as the same information is being delivered via multiple avenues. For example, think of chalk talks, or explaining what you are doing as you demonstrate a procedure.

**Split-Attention effect:**^6-7^ Avoid competing stimuli. Avoid content that is not absolutely necessary, even if it is interesting. For example, when you are initially teaching your mentee how to intubate with a Miller 2 blade, let them finish learning about the Miller 2 before you start explaining how the MAC 3 differs from the Miller 2.

**Objective #2: Discuss general principles of effective teaching:**

Principle #1: Allow sufficient time.

As mentioned above, the cognitive load on your CA-1 is intense. As a trainer, your goal is to match the cognitive load to what the learner needs at each specific point in time. As experienced mentors, we have already automated a significant amount of tasks. This can complicate teaching because something may seem obvious and simple to you, or you may perform the task instinctively without much thought required. However, the trainee is still processing a lot of information and forming those mental connections (**germane cognitive load**). Remember that classroom knowledge is not the same as practical knowledge. For example, reciting all of the steps to placing a central line is very different from actually placing a central line in real life. Be patient and give your mentee enough time to form those mental connections. Multiple sleep cycles are necessary to ingrain the necessary knowledge. **Be careful not to cram too much information into too short a period of time.**


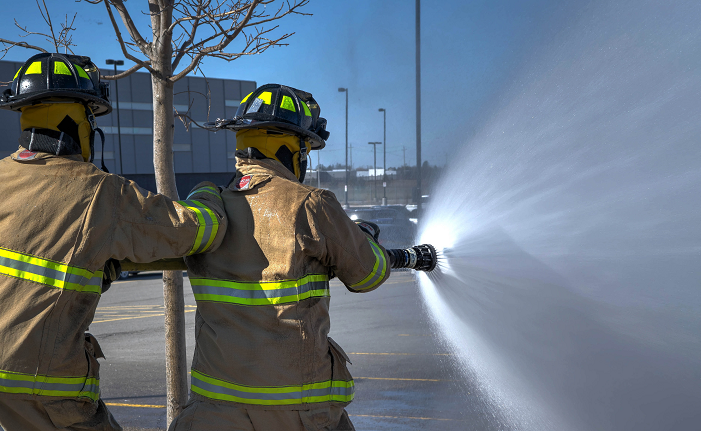


*It is easy to inadvertently be the fire hose for your CA-1. Image by Michael Jeffrey, retrieved from: https://unsplash.com/photos/lgCb_XiLgp8 on 5/16/2020. Image is in the public domain.*

Principle #2: Let them figure it out.

Instead of telling your trainee the answer, use questions and prompts to help guide them to the correct answer.

Example:

Trainer: What do you expect phenylephrine to do to the heart rate?

Trainee: I’m not sure.

Trainer: Okay, what about the mechanism of action?

Trainee: I remember it’s an alpha agonist.

Trainer: That’s right.

Trainee: I think there’s a reflex that slows down the heart rate when the blood pressure goes up?

Trainer: Precisely – that’s the baroreceptor reflex

By making your trainee work a little bit for the answer, you will help form more long-lasting mental connections. Keep in mind that this increases cognitive load, so be careful with this early on during training-in.

Principle #3: Encourage autonomy

What motivates people to want to learn? One answer is **self-determination theory** which breaks down into the following components:^8^

- Relatedness
- Autonomy
- Competence

**Relatedness:** a sense of community – knowing there are other people in the same boat as you.

**Competence:** the capability of an individual to do something successfully AND efficiently.

**Autonomy:** as a trainer, you can directly impact the sense of autonomy. Think back to the last time someone took over your procedure, and think about how you felt.

One of the hardest parts of being a trainer is learning to be hands off. It’s been ingrained in us to help out. **To prevent this, step back and force yourself to keep your arms folded**. This will help you fight the urge to jump in immediately when something happens.

Example: waiting for your trainee to notice that the IV fluid bag is empty, rather than putting up a new bag yourself).

**Objective #3: Evaluate the quality of teaching using a standardized framework:**

These 5 items are adapted from the framework used at Stanford to evaluate teachers. Before you continue reading, take a moment to recall a time when you received excellent teaching. Then think of a time when you received poor teaching. Now keep those 2 experiences in mind as you go through the framework below:

Step 1: Foster a learning climate

Think back to the last time someone yelled at you in the OR. Your learning was probably impaired immediately after the event. For maximum learning, it is important to establish a non-punitive climate and provide psychological safety. Let your trainee know upfront that it is okay to admit when they don’t know the answer. Validate that this is fully expected as a beginner.

Step 2: Set clear goals

It is easier for your trainee to follow along if you let them know beforehand what you are about to cover, so that they have a mental model in place. For example, “Let’s talk about how to place an arterial line” or “Let’s discuss blood pressure support with phenylephrine and ephedrine.”

Step 3: Promote understanding

**Adult learning theory** differs from child learning theory. Children have no basis for comparison and accept information a priori (e.g. “This is the color red”). In contrast, adults learn by relating information back to what they already know. As a teacher, you can promote understanding by **activating prior knowledge**. For example, when talking about atelectasis and lung compliance, ask learners to think of inflating a balloon. The more you can tie a new concept back to something they already know, the better it will stick. Also, be careful to **avoid information overload**, as excessive cognitive load impairs learning.

Step 4: Ask quality questions

There are various types of questions you can ask to **activate prior knowledge**.^9^

1. **Recall**: “What are the criteria for severe aortic stenosis?”
2. **Synthesis**: “What will happen to the left ventricle with longstanding severe aortic stenosis?”
3. **Application**: “What are your hemodynamic goals for a patient with severe aortic stenosis?”

**Recall** questions are the easiest ones to ask, but the least effective at promoting learning. Higher-order **Synthesis** and **Application** questions allow you to challenge learners to think more deeply and form those mental connections.

Step 5: Seek out feedback

Ensure that there is two-way communication so that you and your trainee remain on the same page. For example: Did that make sense? Am I moving too quickly? Too slowly?

**Objective #4: Learn how to use microskills for clinical teaching:**

**Microskills** allow you to quickly assess what your trainee knows and then provide targeted teaching, all in the span of a few minutes.^10-11^ Note that this increases cognitive load, so this should be avoided during the initial phase of training-in when the trainee is already being overwhelmed with information. However, microskills may be helpful for developing critical thinking in the trainee once they have the basics down, potentially during the last week of training-in, and certainly later on throughout the rest of the year.

Step 1: Get a commitment

Ask your trainee to select a possible explanation. It’s okay if it turns out that they are incorrect. The purpose of the exercise is to develop their critical thinking skills.

Step 2: Probe for supporting evidence

Ask your trainee to look for clinical evidence that supports or argues against their hypothesis. Remember, this is still the fact-gathering stage – do not confirm or deny their hypothesis yet.

Step 3: Teach general rules

Explain how you would approach a similar situation. What are the most common explanations for this scenario? What critical issues do you need to rule out?

Step 4: Reinforce what was done right

Now for the feedback: start by highlighting the things they did well.

Step 5: Correct mistakes

And finally, explain any errors that they made and how to correct them.

-----------------------

Example: End tidal CO_2_ is increasing during a laparoscopic procedure.

Step 1: Get a commitment:

This is malignant hyperthermia.

Step 2: Probe for supporting evidence:

Temperature is normal

No muscle rigidity

Mildly hypertensive

Minute ventilation is unchanged

Step 3: Teach general rules:

Laparoscopy is performed with CO2 insufflation, which can result in hypercapnia.

Step 4: Reinforce what was done right:

Good job examining their masseter muscle.

I like that you considered a potentially life-threatening diagnosis on your differential.

Step 5: Correct mistakes:

It is important to keep an eye on what the surgeons are doing. Expect hypercarbia during laparoscopy and consider increasing minute ventilation prophylactically. Ruling out both common things as well as life threatening events is important in what we do for the patients.

-----------------------

**Strengths:**

- Very quick to use.
- Ability to assess the learner’s knowledge base and selectively target your teaching.
- Delivers small pearls of wisdom to avoid overwhelming the learner with material.

**Limitations:**

- Increases cognitive load.
- Ineffective if learner has little knowledge to draw on and is making a blind guess from the beginning without any reasoning behind it.
- Not designed for teaching procedural skills.

**References:**

1. Miller GA. The Magical Number Seven, Plus or Minus Two: Some Limits on our Capacity for Processing Information. Psychological Review. 1956;63: 81-97.
2. Chase WG, Simon HA. Perception in chess. Cognitive Psychology. 1973;4(1): 55–81.
3. <https://en.wikipedia.org/wiki/Automaticity>. Accessed on September 1, 2020.
4. Clark R, Nguyen F, Sweller J. Kilgore D, ed. Efficiency in Learning: Evidence-Based Guidelines to Manage Cognitive Load. San Francisco, CA: Pfeiffer; 2006.
5. Sweller J. The worked example effect and human cognition. Learning and Instruction. 2006;16: 165-169.
6. Chandler P, Sweller J. The Split-Attention Effect as a Factor in the Design of Instruction. Educational Psychology. 1992;62(2): 233-246.
7. Mayer RE, Moreno R. Nine ways to reduce cognitive load in multimedia learning. Educational Psychologist. 2003;38(1): 43-52.
8. Ryan RM, Deci EL. Self-determination theory and the facilitation of intrinsic motivation, social development, and well-being. Am Psychol. 2000;55(1):68-78.
9. Bloom B, Englehart MD, Furst E, Hill WH, Krathwohl DR. Taxonomy of educational objectives: The classification of educational goals. Handbook I: Cognitive domain. New York: David McKay Company; 1956.
10. Neher JO, Gordon KC, Meyer B, Stevens N. A five-step “microskills” model of clinical teaching. J Am Board Fam Pract. 1992;5: 419-24.
11. Neher JO, Stevens NG. The One-minute Preceptor: Shaping the Teaching Conversation. Fam Med. 2003;35(6): 391-3.
