# Supplementary material for: Training the Trainer: Preparing Anesthesiology Residents to be Trainers in the Operating Room
Source: MedEdPORTAL. 2021 Mar 4;17:11116. doi: 10.15766/mep_2374-8265.11116 (PMC7970634; doi:10.15766/mep_2374-8265.11116)
Supplement: Supplementary file 1 — Primer Document.docxWorkshop Handout.docxWorkshop PowerPoint.pptxInstructor Manual.docxPresurvey.pdfPostsurvey.pdf1-Week Follow-up Survey.docx1-Month Follow-up Survey.docxNew CA 1 Survey.docx [file mep_2374-8265.11116-s001.zip › B. Workshop Handout.docx]

**Cognitive Load and Effective Teaching**

**Cognitive load:**

Q1. What are the three types of cognitive load?

1.

2.

3.

Q2. What are ways to reduce cognitive load? Give an example of how you can apply each method.

1.

2.

3.

4.

5.

**Effective teaching:**

Q3. What are the components of self-determination theory?

1.

2.

3.

**Evaluating quality of teaching:**

*(adapted from Susan Farrell, MD, EdMl)*

Q4. A) Think of your “best” learning experience. What were you learning?

B) What was the teacher doing?

C) What were you doing?

D) How did the learning make you feel?

Q5. A) What are 5 steps for evaluating the quality of teaching? B) Explain the importance of each step.

1.

2.

3.

4.

5.

**Types of questions:**

Q6. Select your favorite medical condition or pathophysiology.

Write an example of a Recall question.

Write an example of a Synthesis question.

Write an example of an Application question.

**Microteaching:**

Q7. Choose a simple task to teach your partner within 5 minutes (May be outside of medicine). Your partner will then provide you with feedback using the above 5 criteria during this microteaching session.

**Five Microskills for Clinical Teaching**

(aka. One-minute Preceptor)

**List and describe the 5 microskills.**

1)

2)

3)

4)

5)

**Scenario:**

You are the senior resident on call. Your partner (the junior resident on call) asks you for advice on a patient in the PACU who is experiencing one of the following events. Practice microskills teaching with your partner. Feel free to make up additional vital signs or relevant data points for your practice scenario.

1. Hypertension (full bladder – junior resident thinks it is pain).
2. Low oxygen saturations (COPD – junior resident thinks it is too much narcotic).
3. Tachycardia (missed a dose of a beta blocker – junior resident thinks it is hypovolemia).
4. Somnolence (too much narcotic – junior resident thinks it is residual anesthetic).

**References:**

1. Neher JO, Gordon KC, Meyer B, Stevens N. A five-step “microskills” model of clinical teaching. J Am Board Fam Pract. 1992;5: 419-24.
2. Neher JO, Stevens NG. The One-minute Preceptor: Shaping the Teaching Conversation. Fam Med. 2003;35(6): 391-3.
