# Supplementary material for: Training the Trainer: Preparing Anesthesiology Residents to be Trainers in the Operating Room
Source: MedEdPORTAL. 2021 Mar 4;17:11116. doi: 10.15766/mep_2374-8265.11116 (PMC7970634; doi:10.15766/mep_2374-8265.11116)
Supplement: Supplementary file 1 — Primer Document.docxWorkshop Handout.docxWorkshop PowerPoint.pptxInstructor Manual.docxPresurvey.pdfPostsurvey.pdf1-Week Follow-up Survey.docx1-Month Follow-up Survey.docxNew CA 1 Survey.docx [file mep_2374-8265.11116-s001.zip › C. Workshop PowerPoint.pptx]

## Slide 1
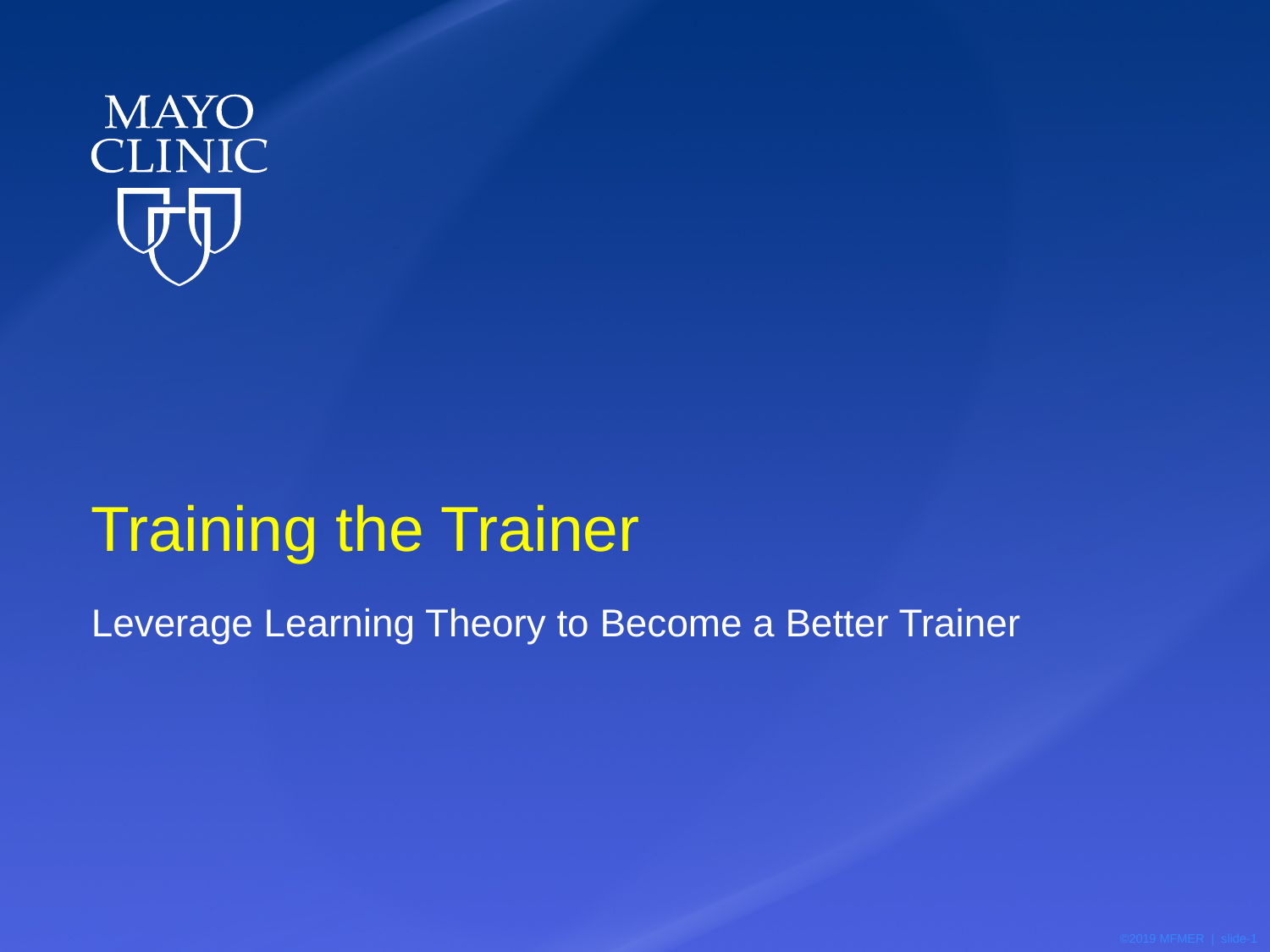

# Training the Trainer
Leverage Learning Theory to Become a Better Trainer

## Slide 2
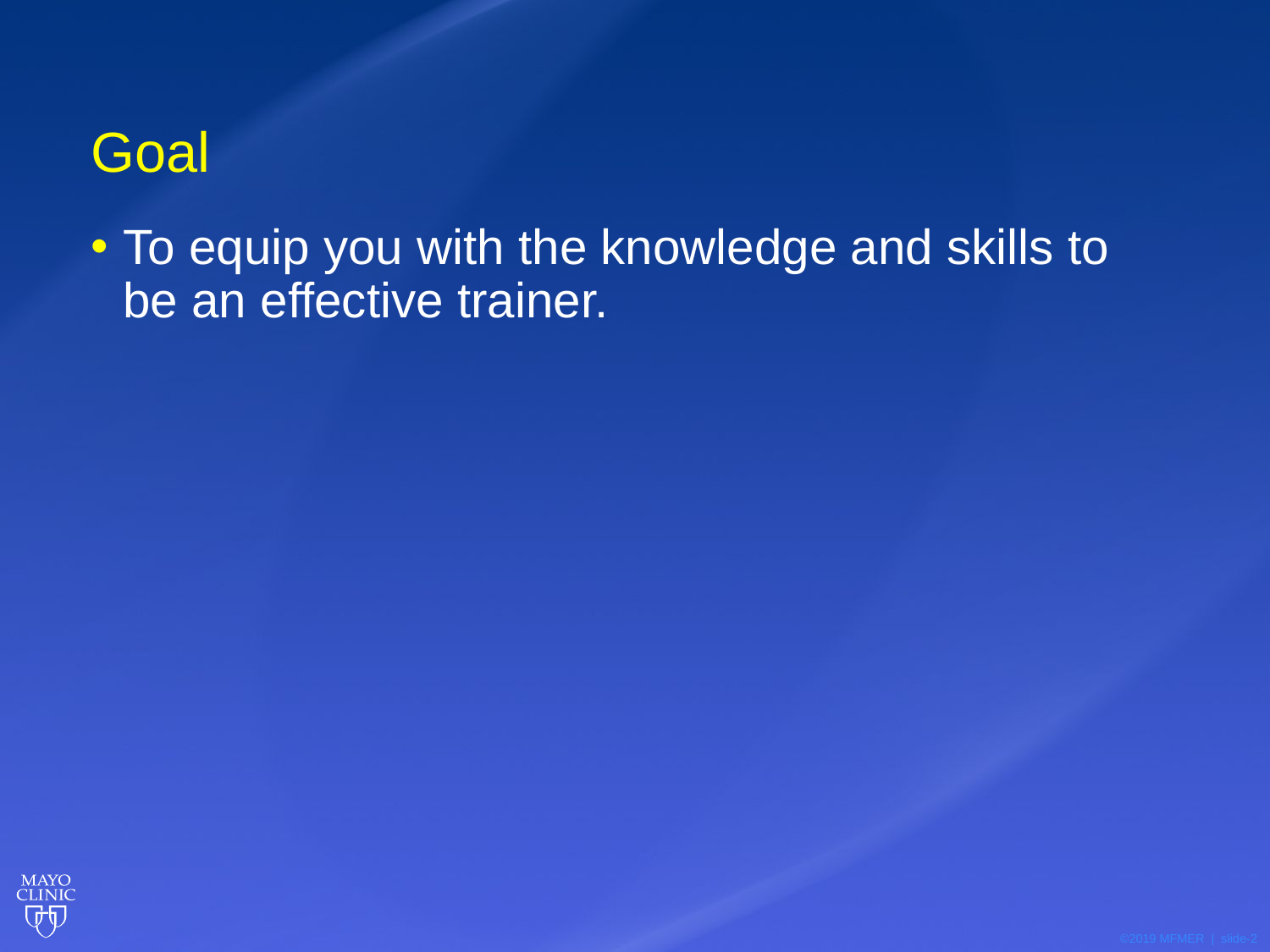

# Goal
To equip you with the knowledge and skills to be an effective trainer.

## Slide 3
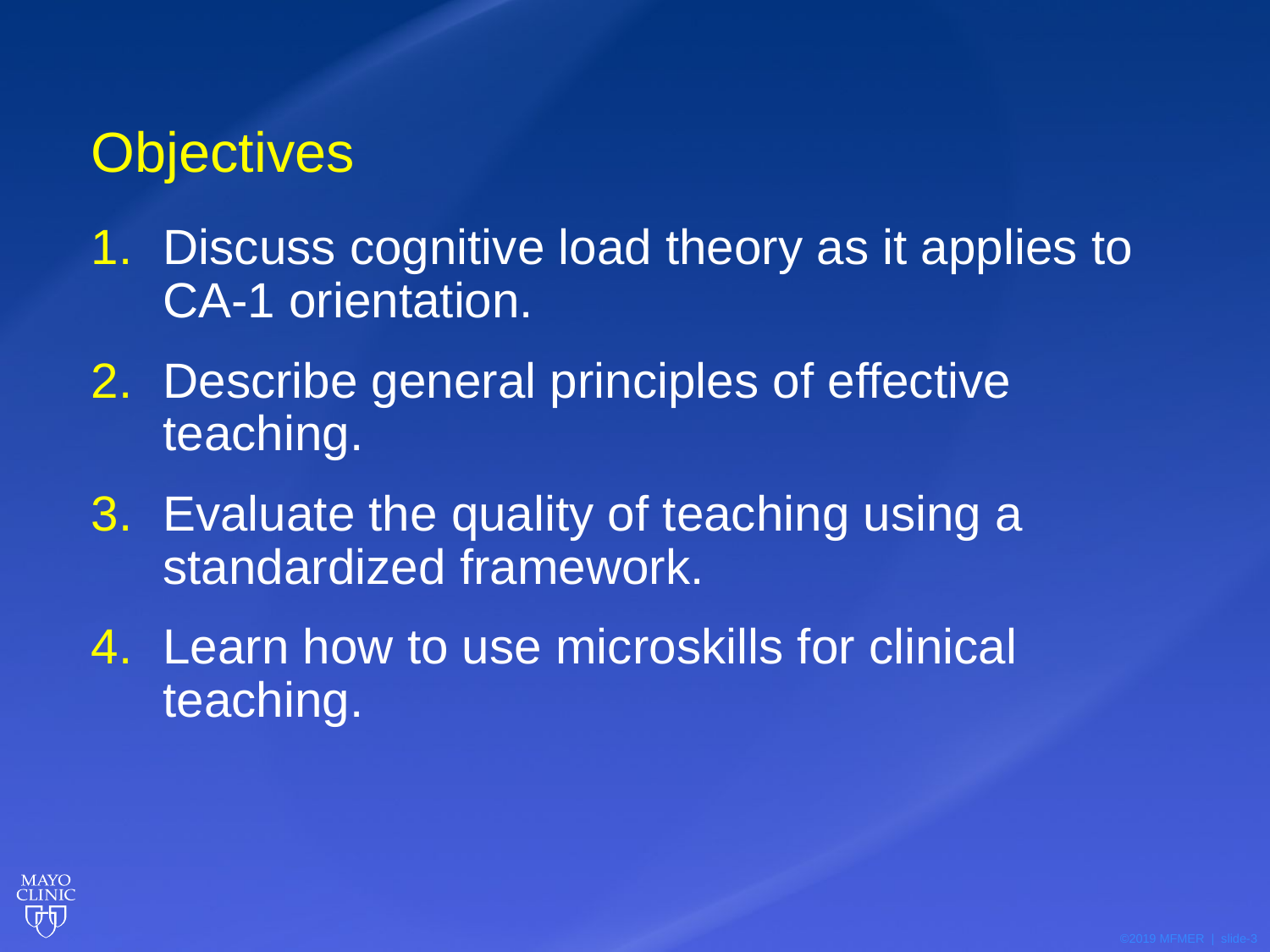

# Objectives
Discuss cognitive load theory as it applies to CA-1 orientation.
Describe general principles of effective teaching.
Evaluate the quality of teaching using a standardized framework.
Learn how to use microskills for clinical teaching.

## Slide 4
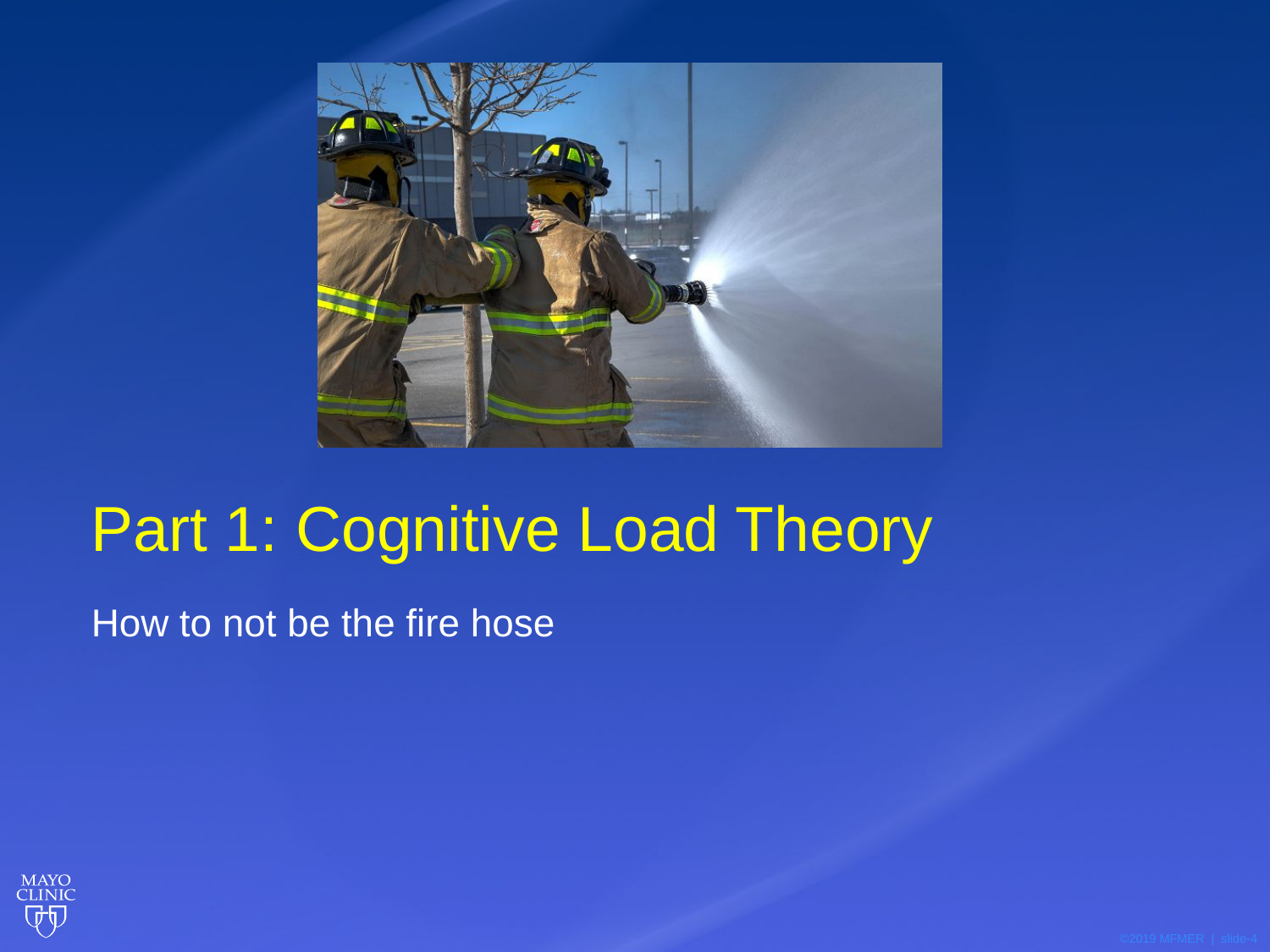

# Part 1: Cognitive Load Theory
How to not be the fire hose

## Slide 5
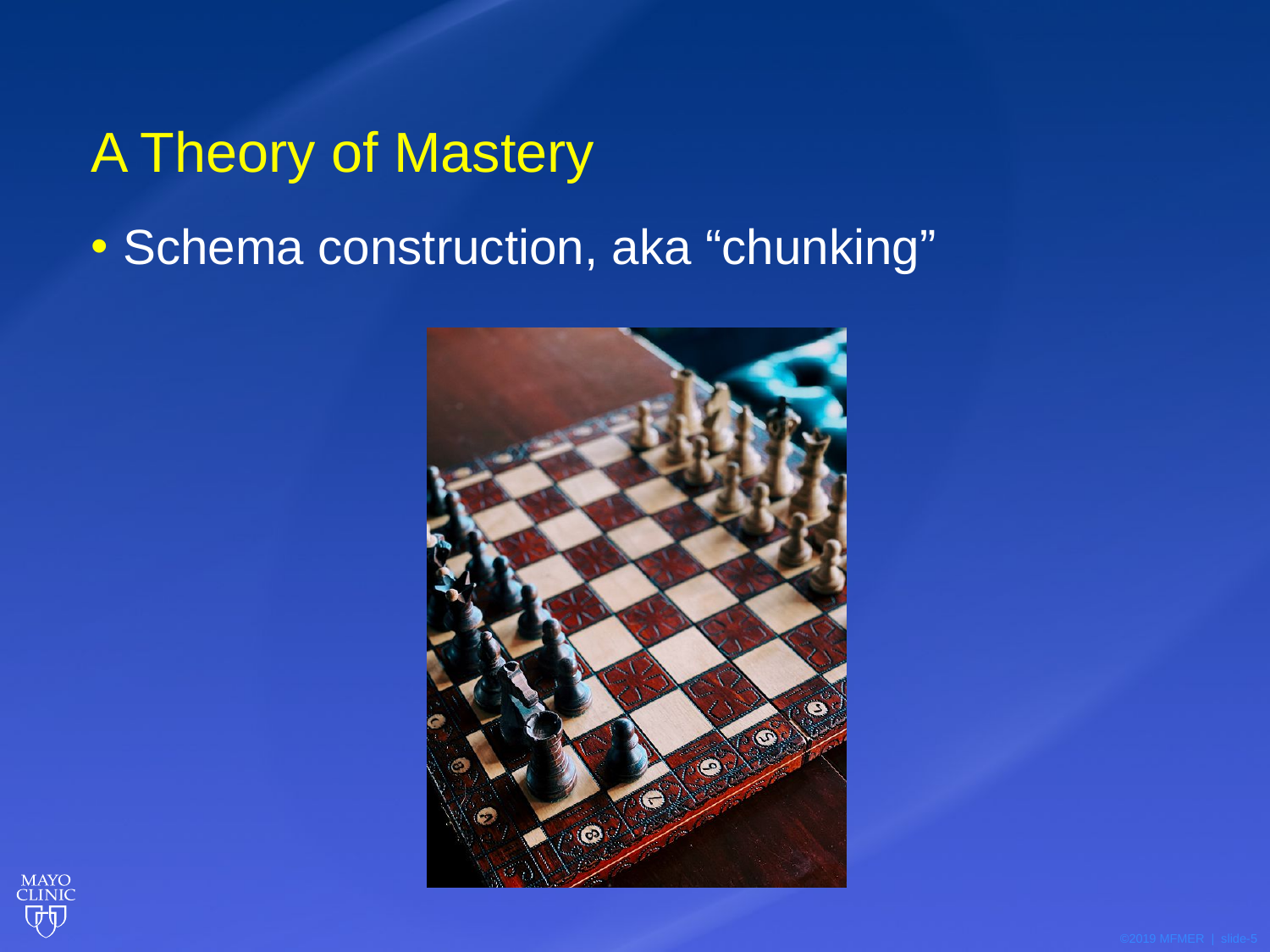

# A Theory of Mastery
Schema construction, aka “chunking”

## Slide 6
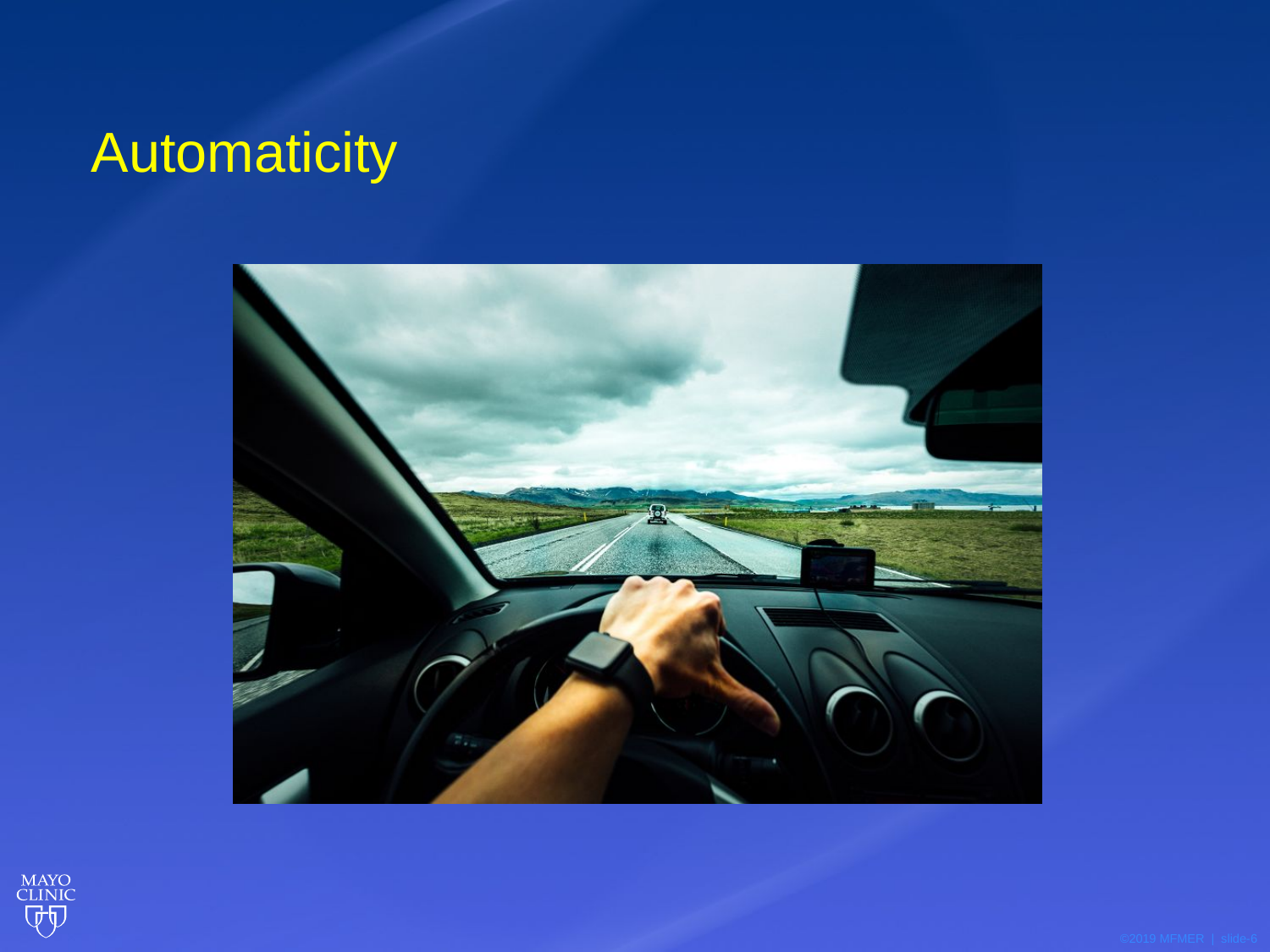

# Automaticity

## Slide 7
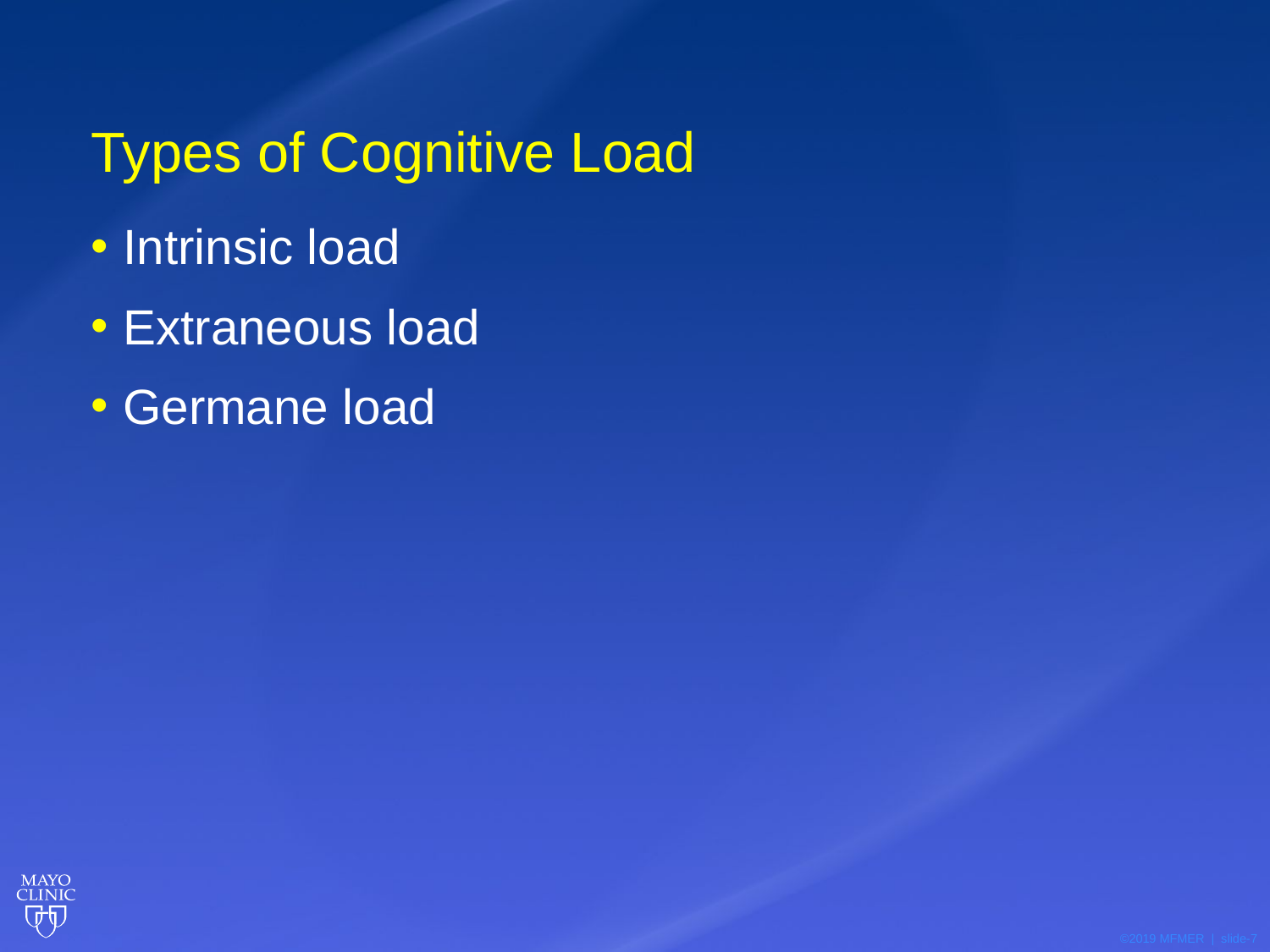

# Types of Cognitive Load
Intrinsic load
Extraneous load
Germane load

## Slide 8
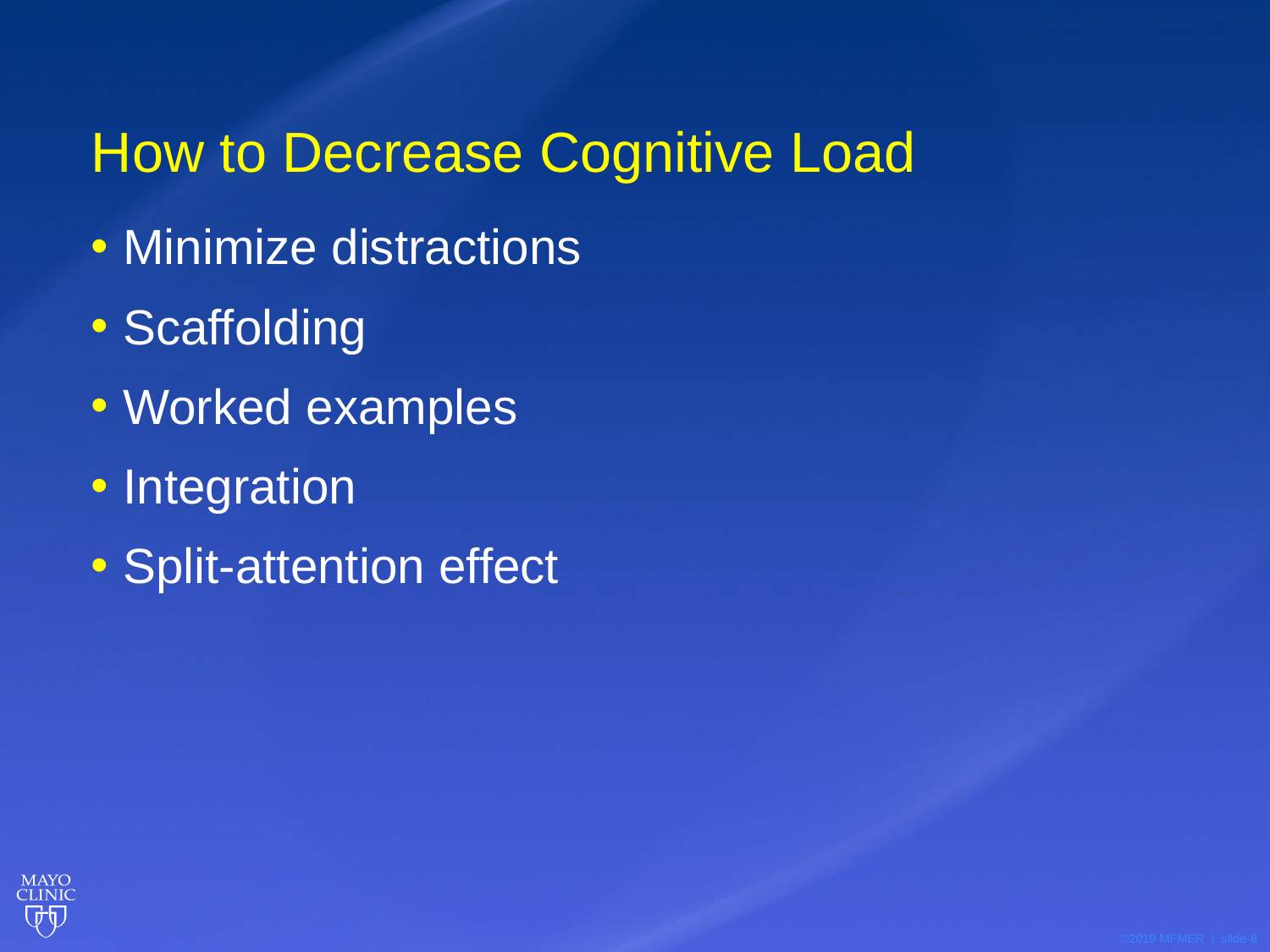

# How to Decrease Cognitive Load
Minimize distractions
Scaffolding
Worked examples
Integration
Split-attention effect

## Slide 9
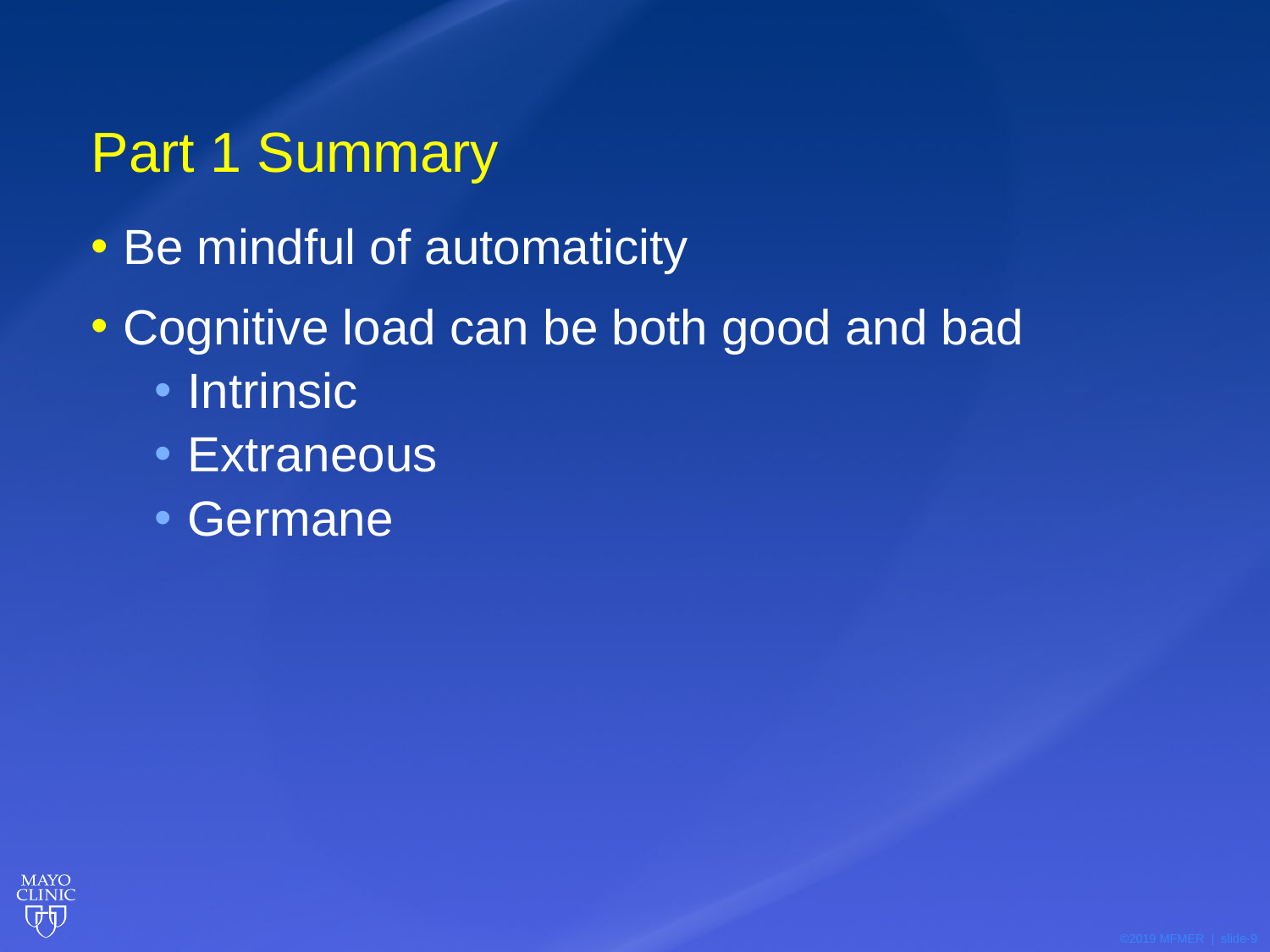

# Part 1 Summary
Be mindful of automaticity
Cognitive load can be both good and bad
Intrinsic
Extraneous
Germane

## Slide 10
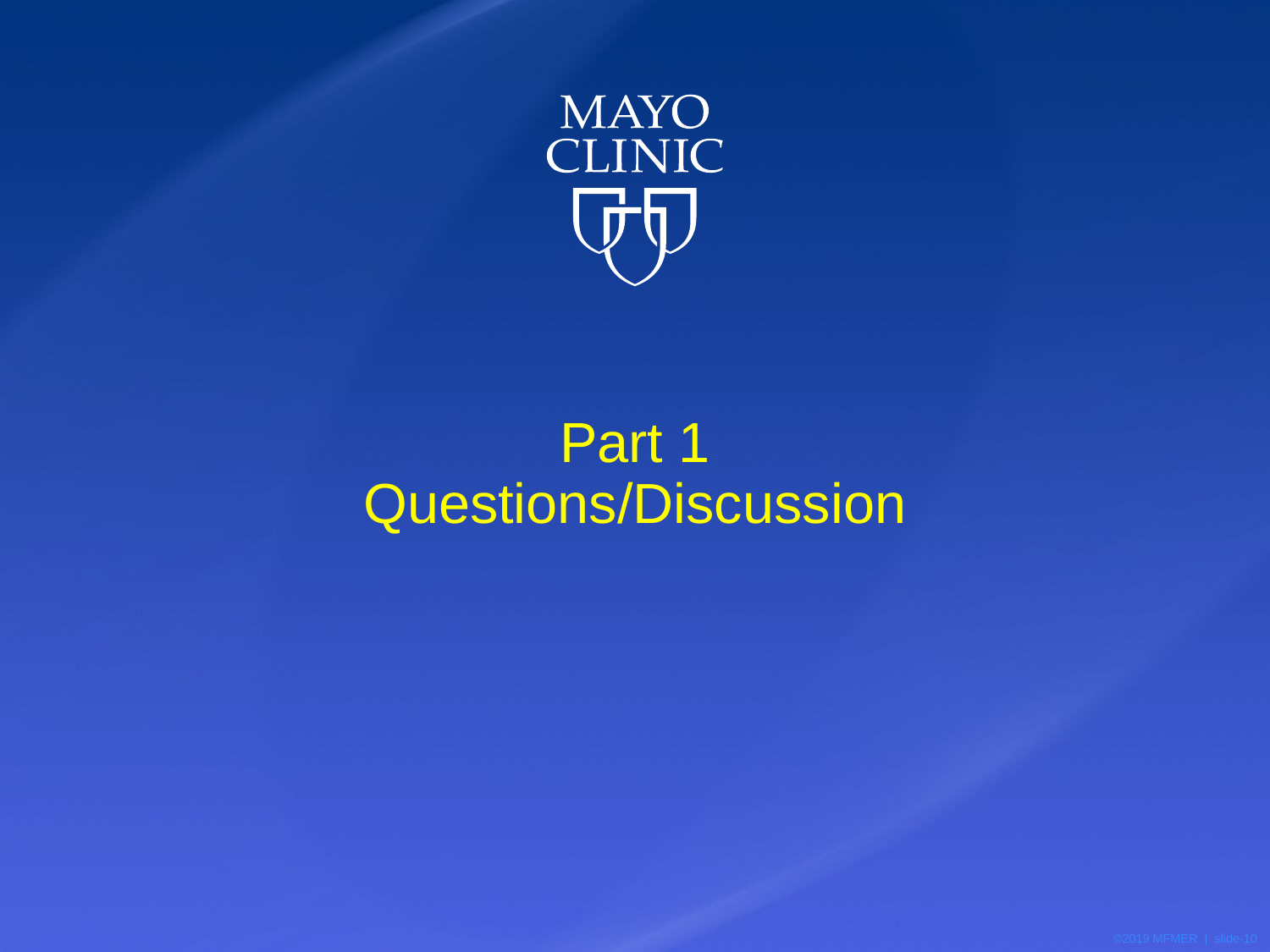

# Part 1 Questions/Discussion

## Slide 11
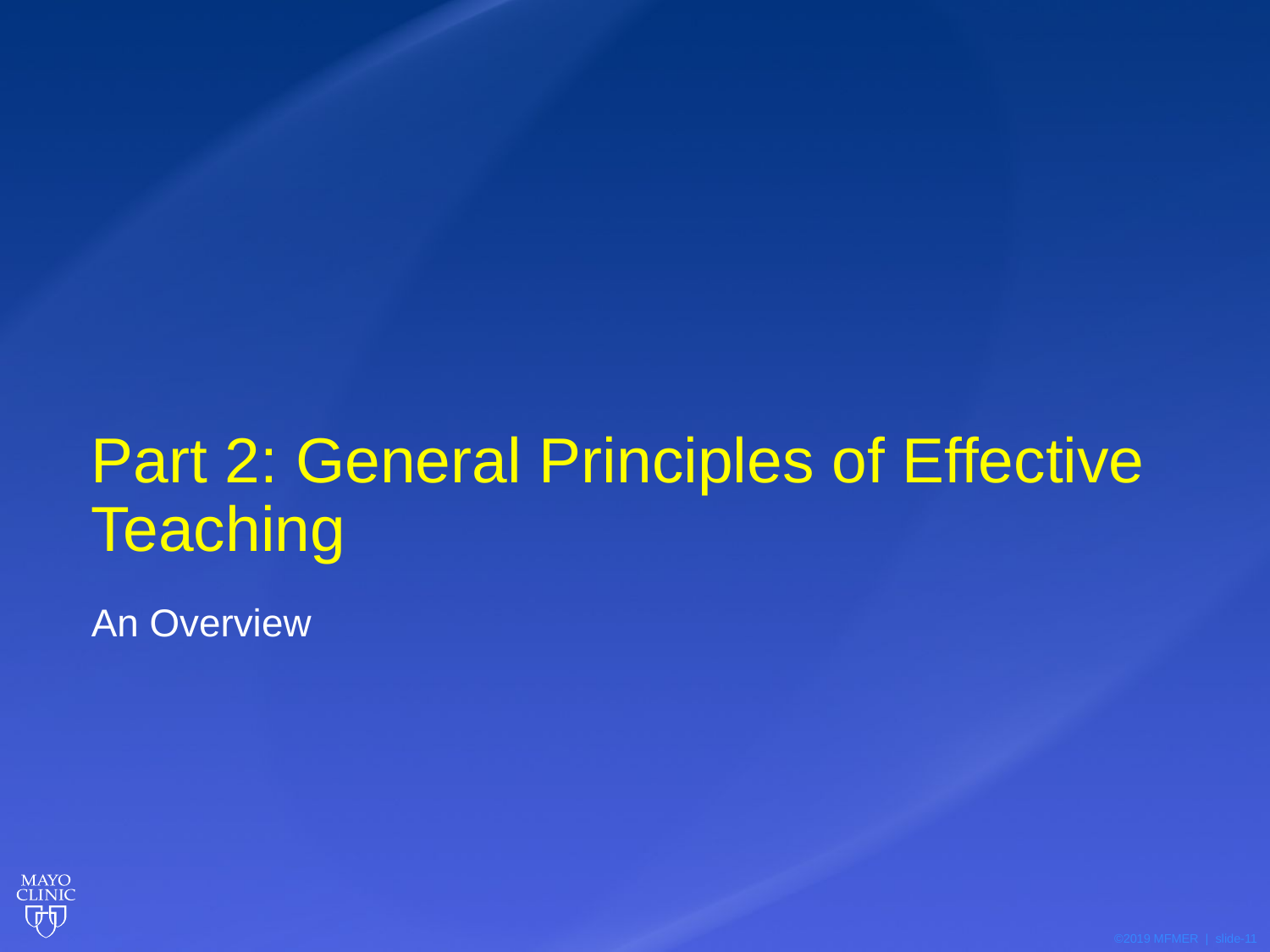

# Part 2: General Principles of Effective Teaching
An Overview

## Slide 12
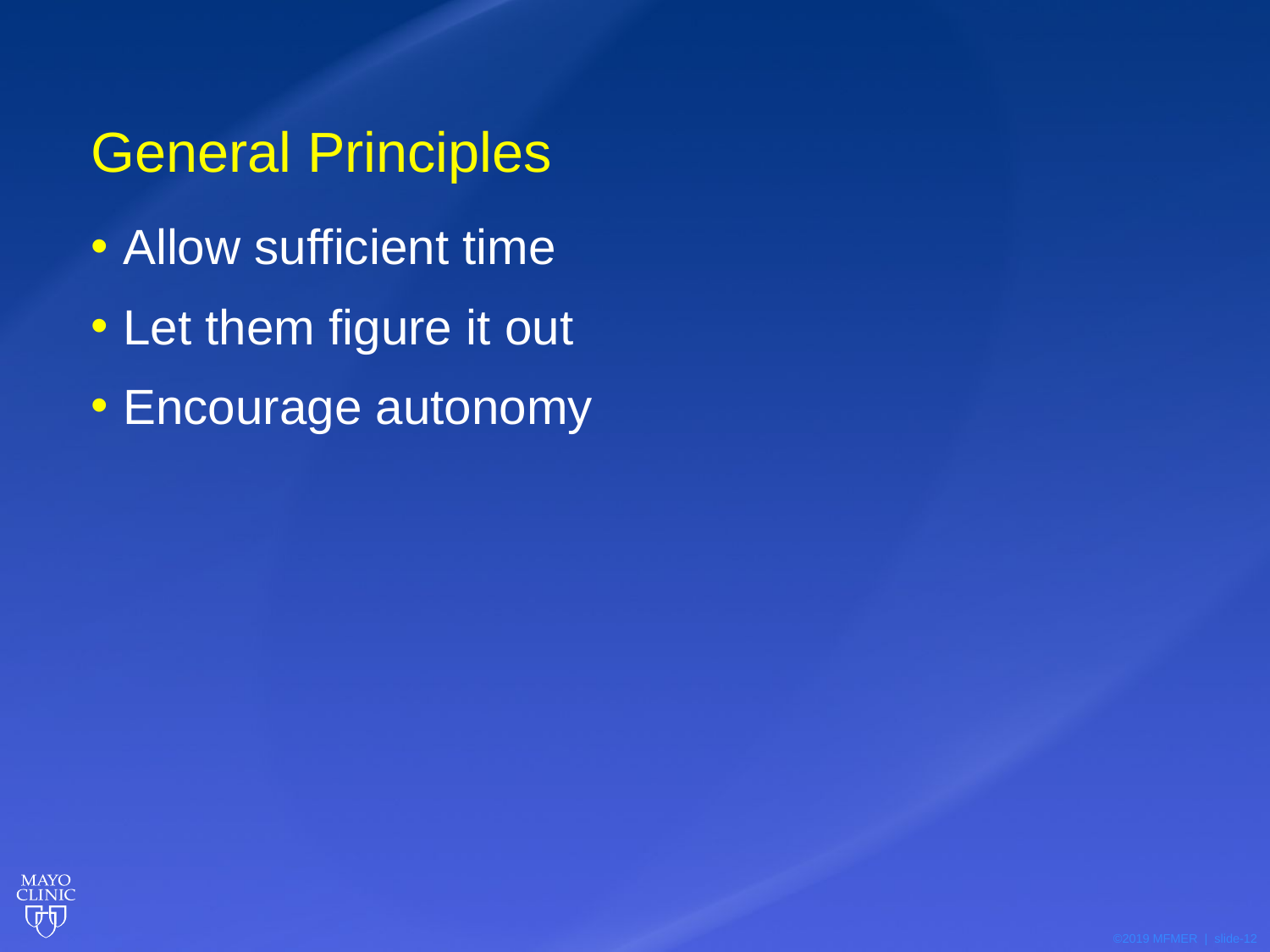

# General Principles
Allow sufficient time
Let them figure it out
Encourage autonomy

## Slide 13
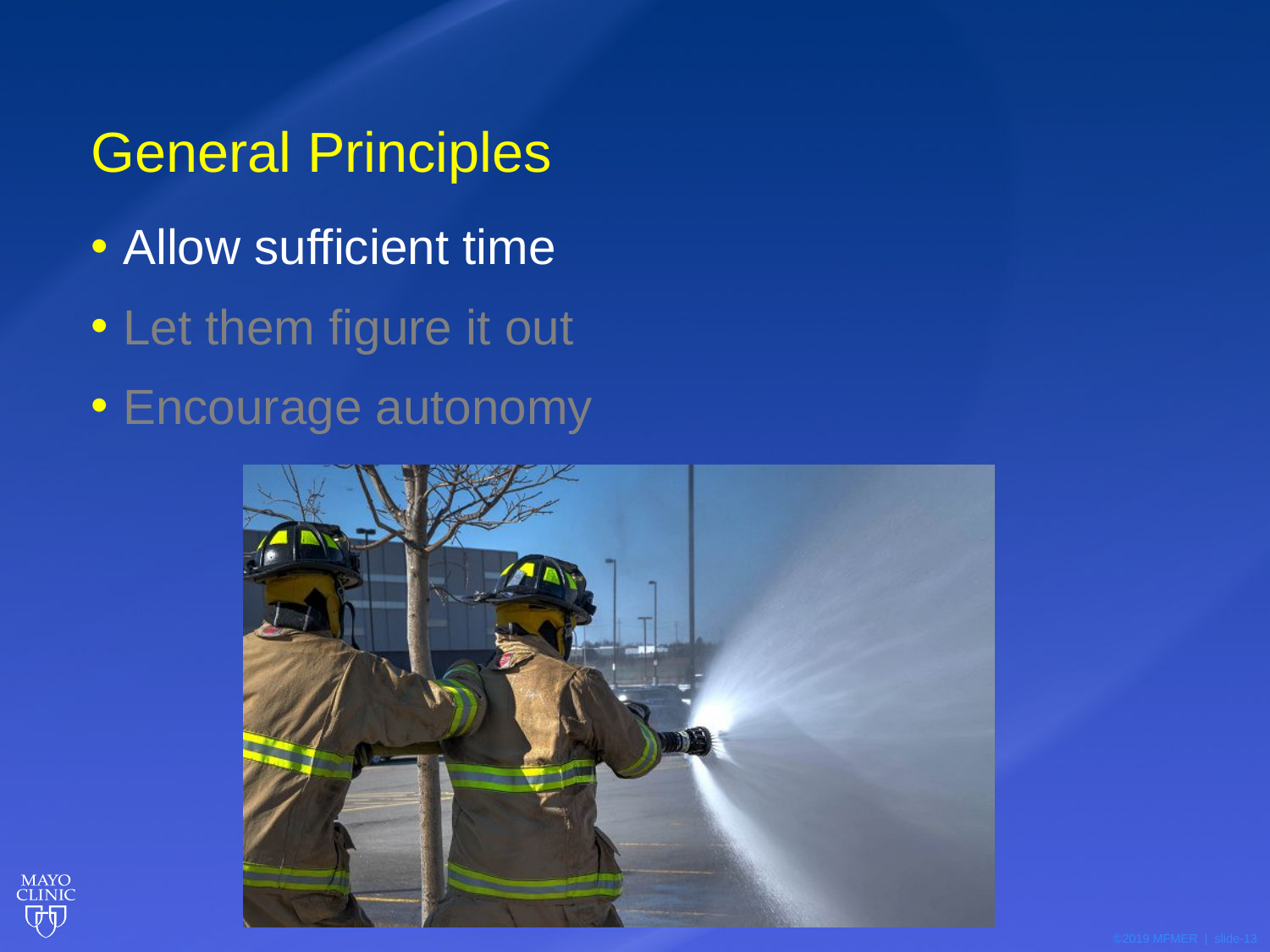

# General Principles
Allow sufficient time
Let them figure it out
Encourage autonomy

## Slide 14
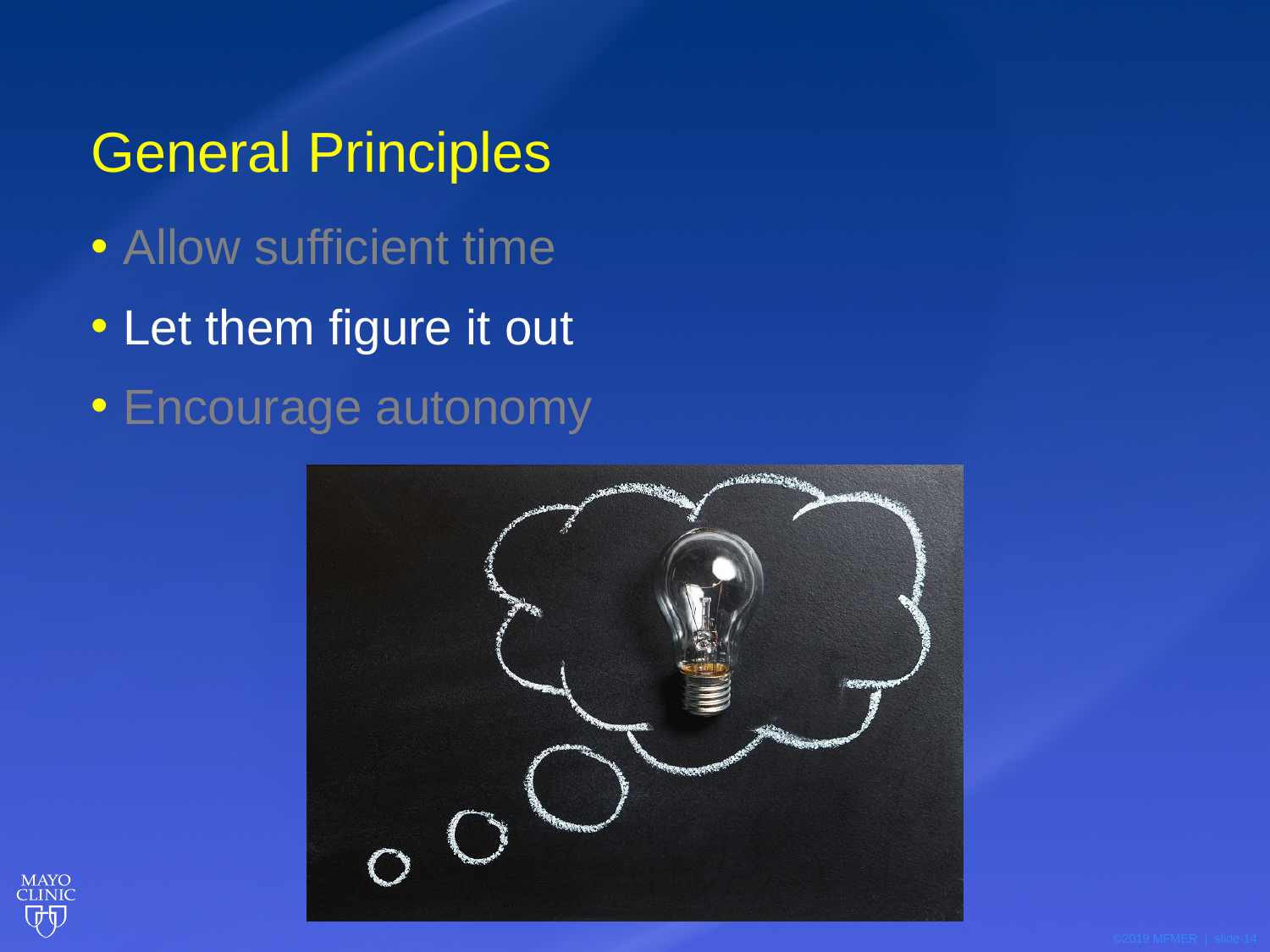

# General Principles
Allow sufficient time
Let them figure it out
Encourage autonomy

## Slide 15
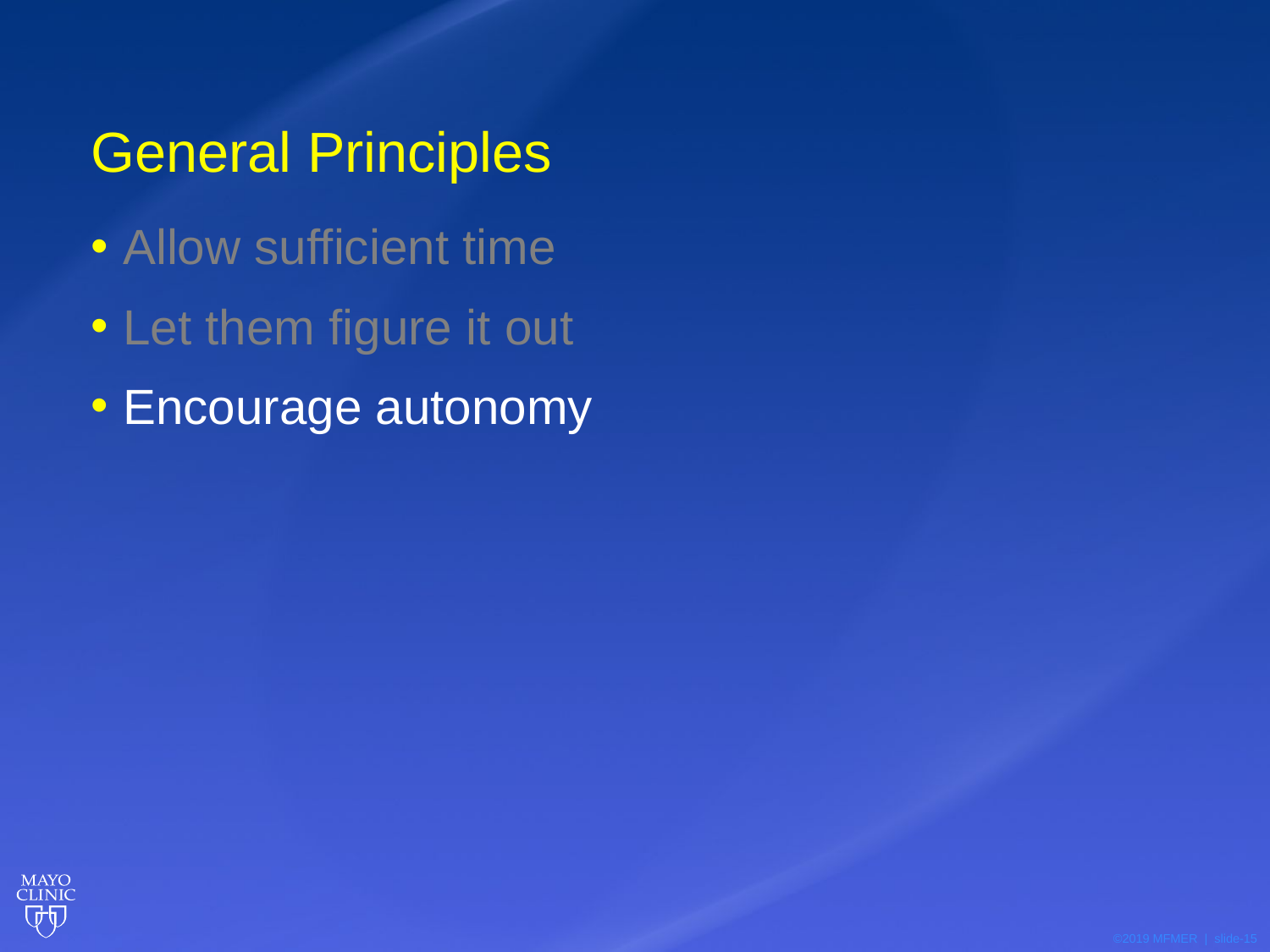

# General Principles
Allow sufficient time
Let them figure it out
Encourage autonomy

## Slide 16
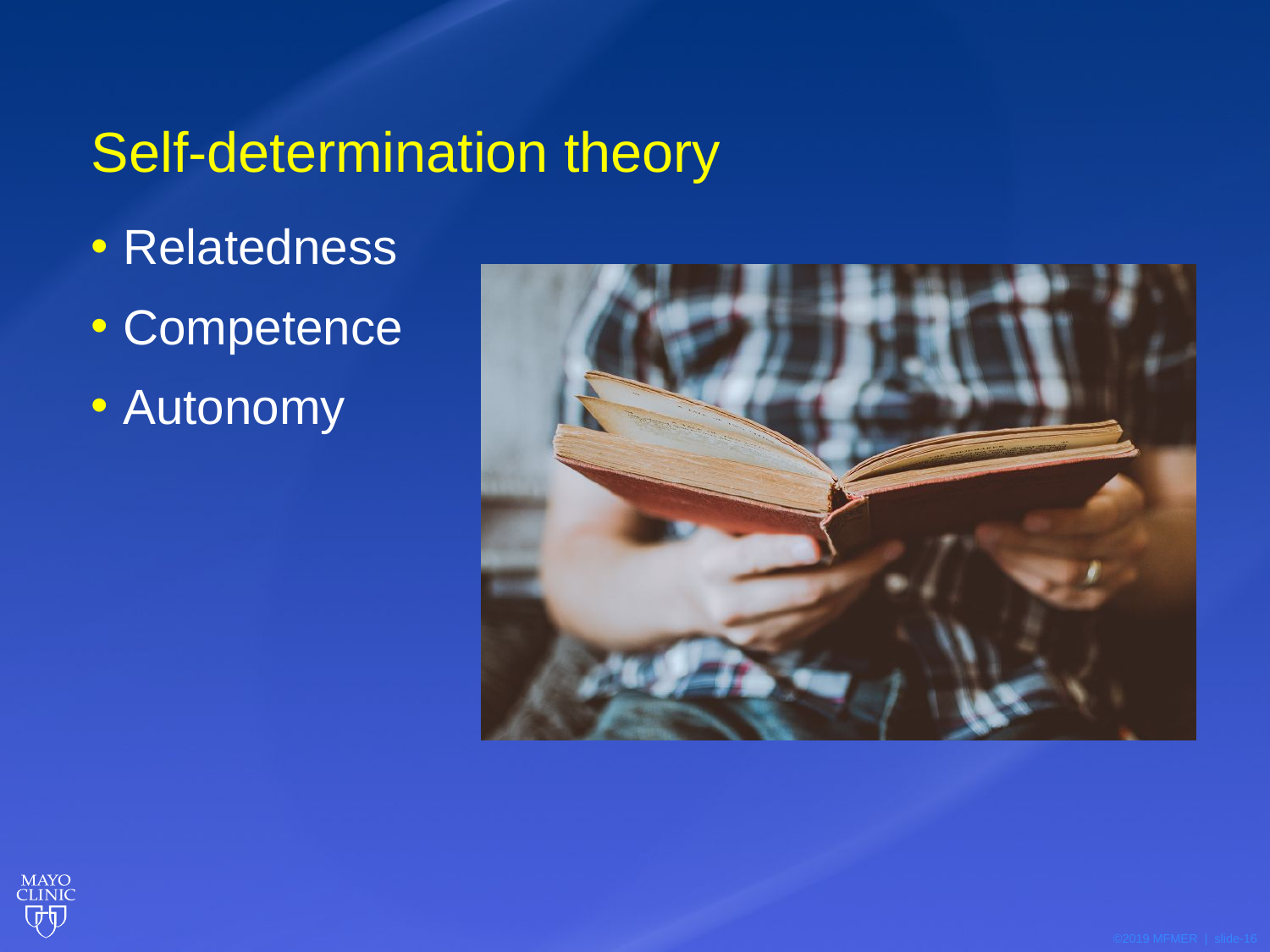

# Self-determination theory
Relatedness
Competence
Autonomy

## Slide 17
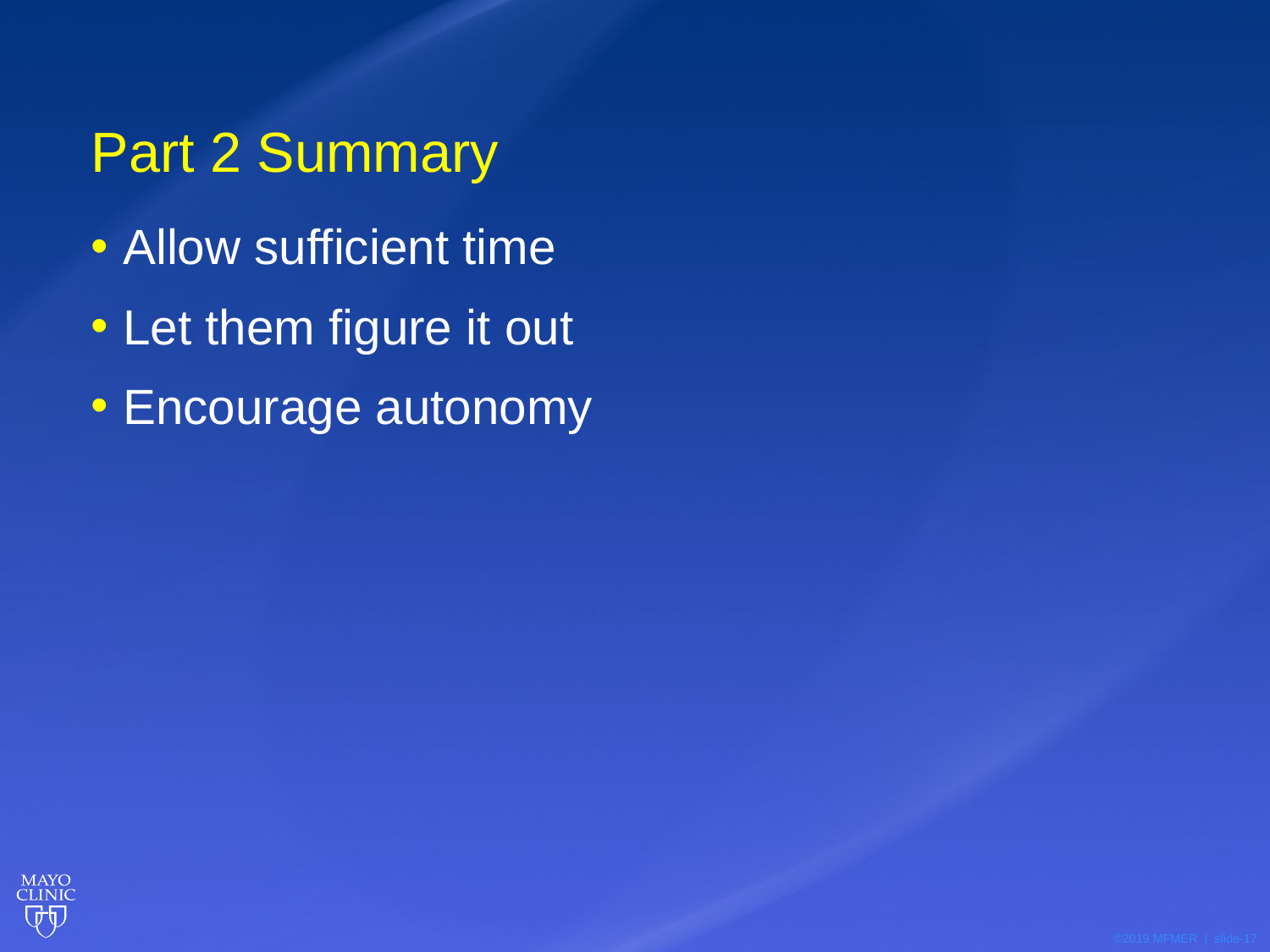

# Part 2 Summary
Allow sufficient time
Let them figure it out
Encourage autonomy

## Slide 18
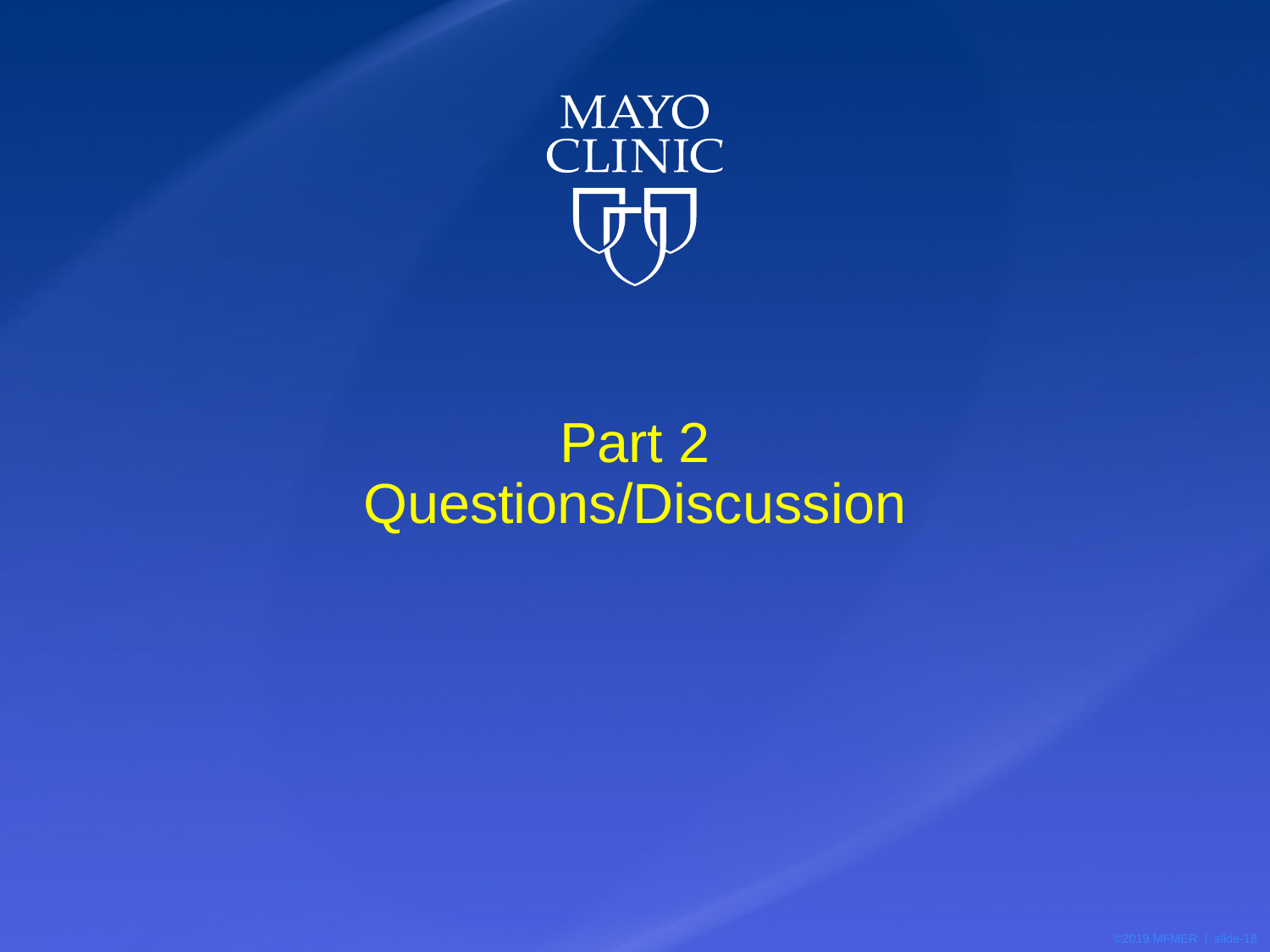

# Part 2 Questions/Discussion

## Slide 19
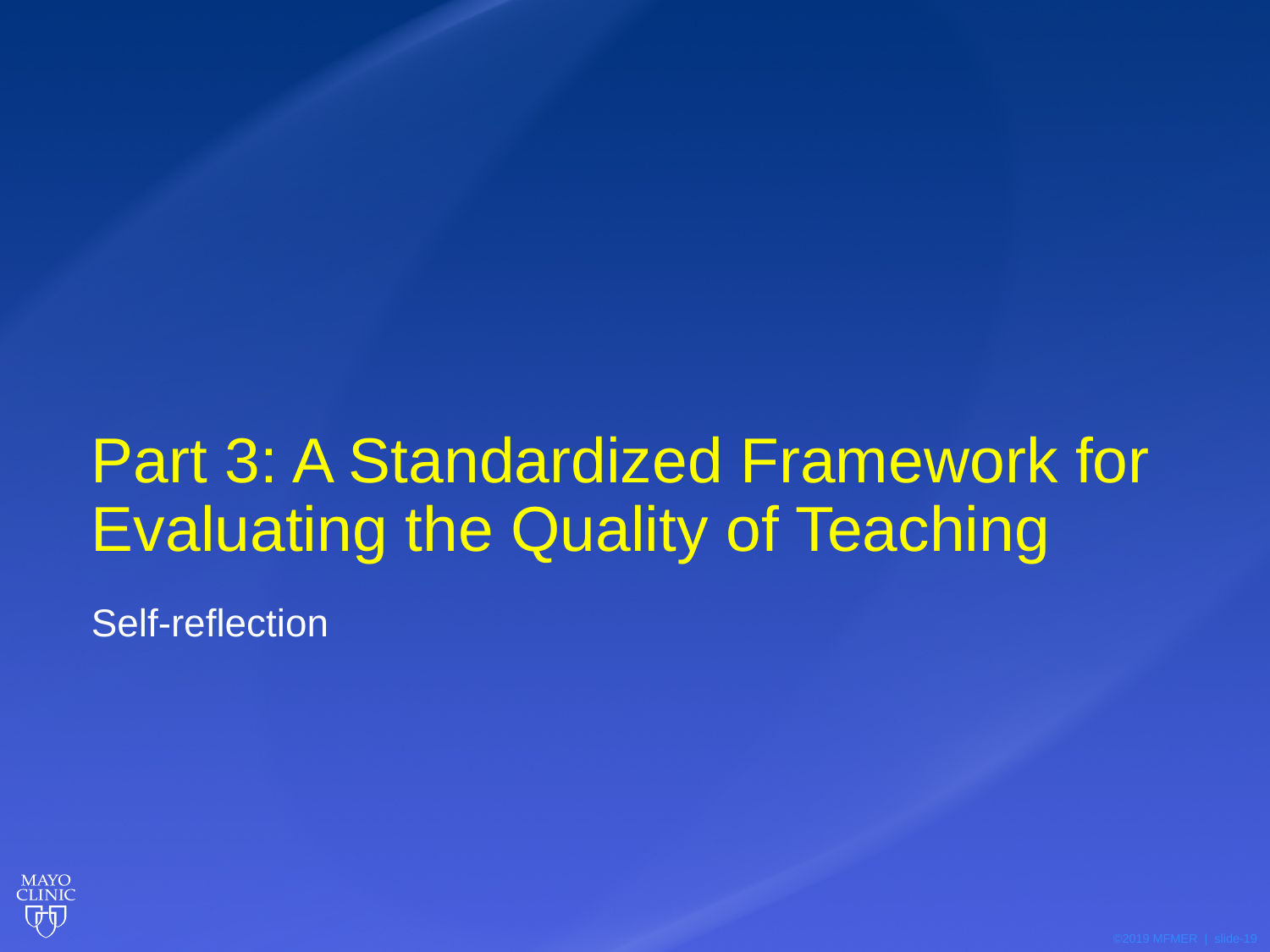

# Part 3: A Standardized Framework for Evaluating the Quality of Teaching
Self-reflection

## Slide 20
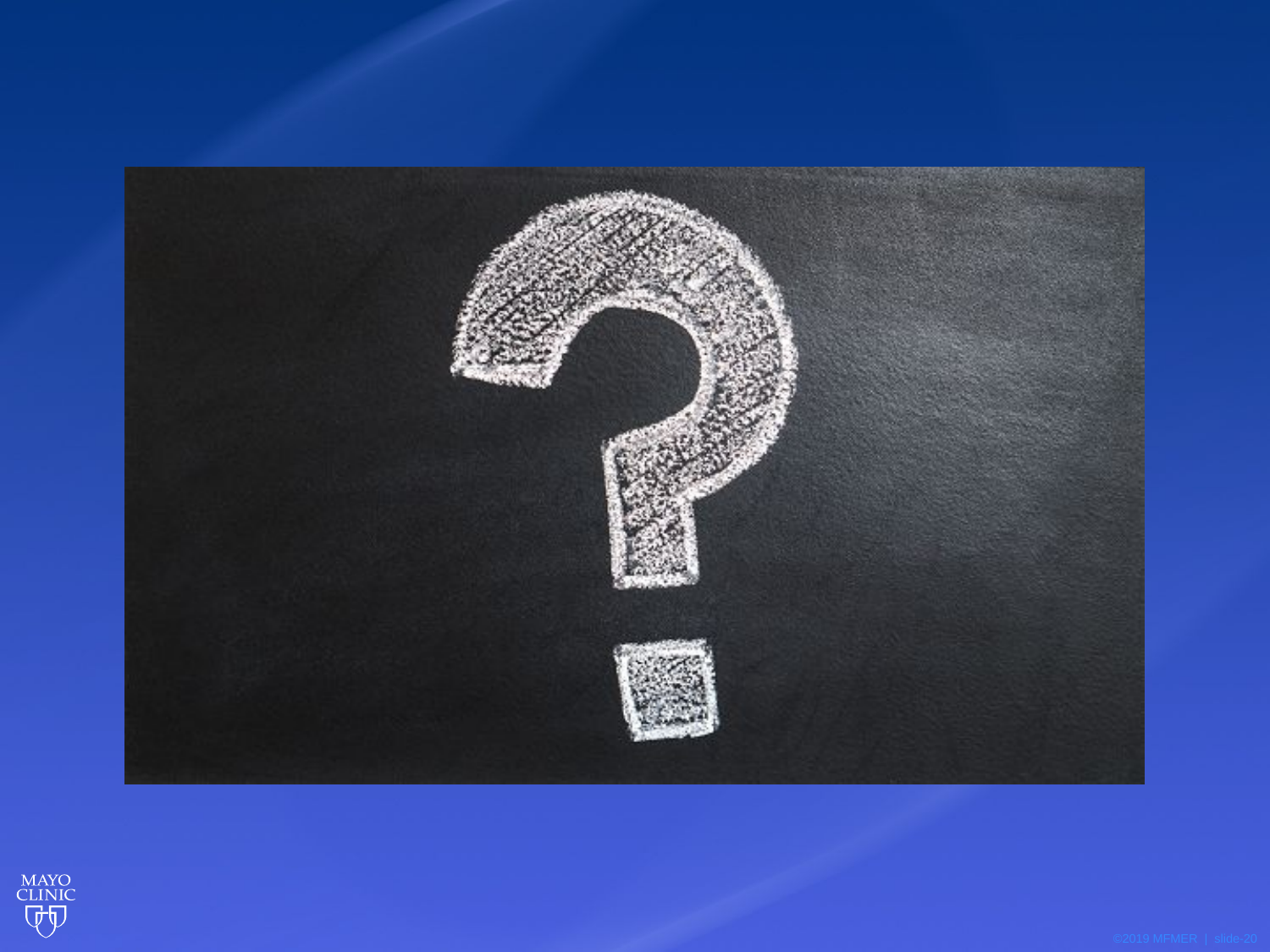

## Slide 21
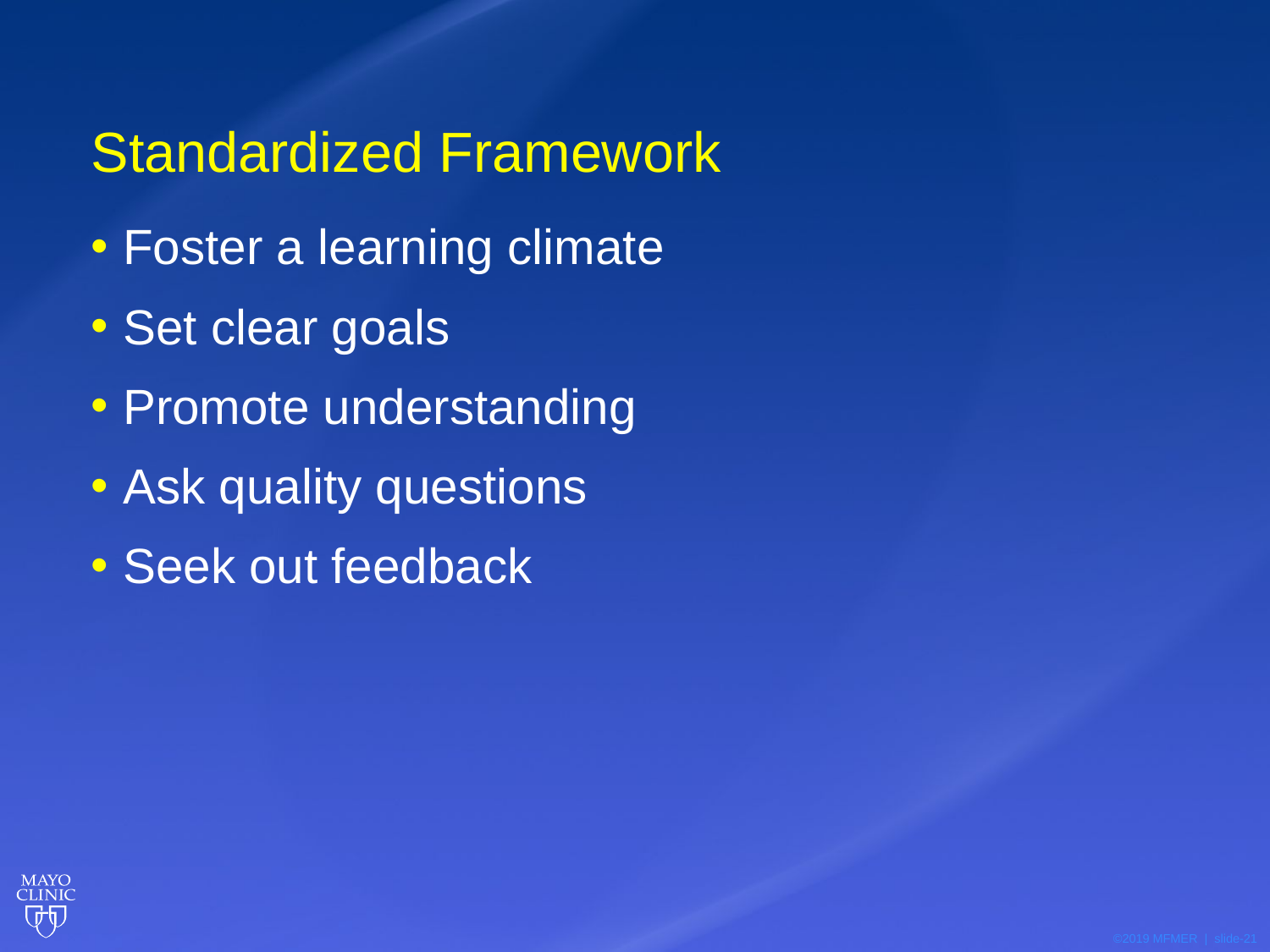

# Standardized Framework
Foster a learning climate
Set clear goals
Promote understanding
Ask quality questions
Seek out feedback

## Slide 22
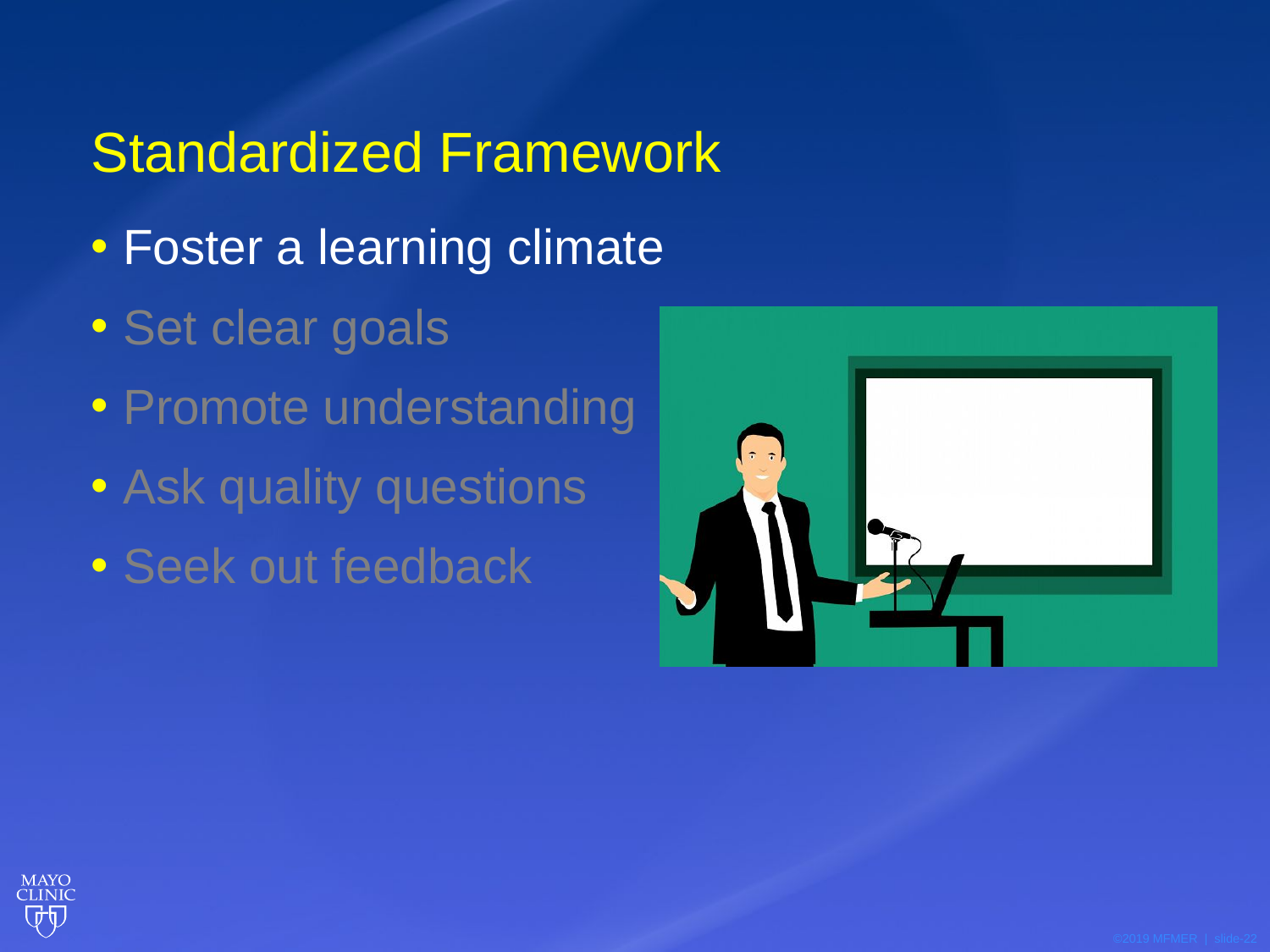

# Standardized Framework
Foster a learning climate
Set clear goals
Promote understanding
Ask quality questions
Seek out feedback

## Slide 23
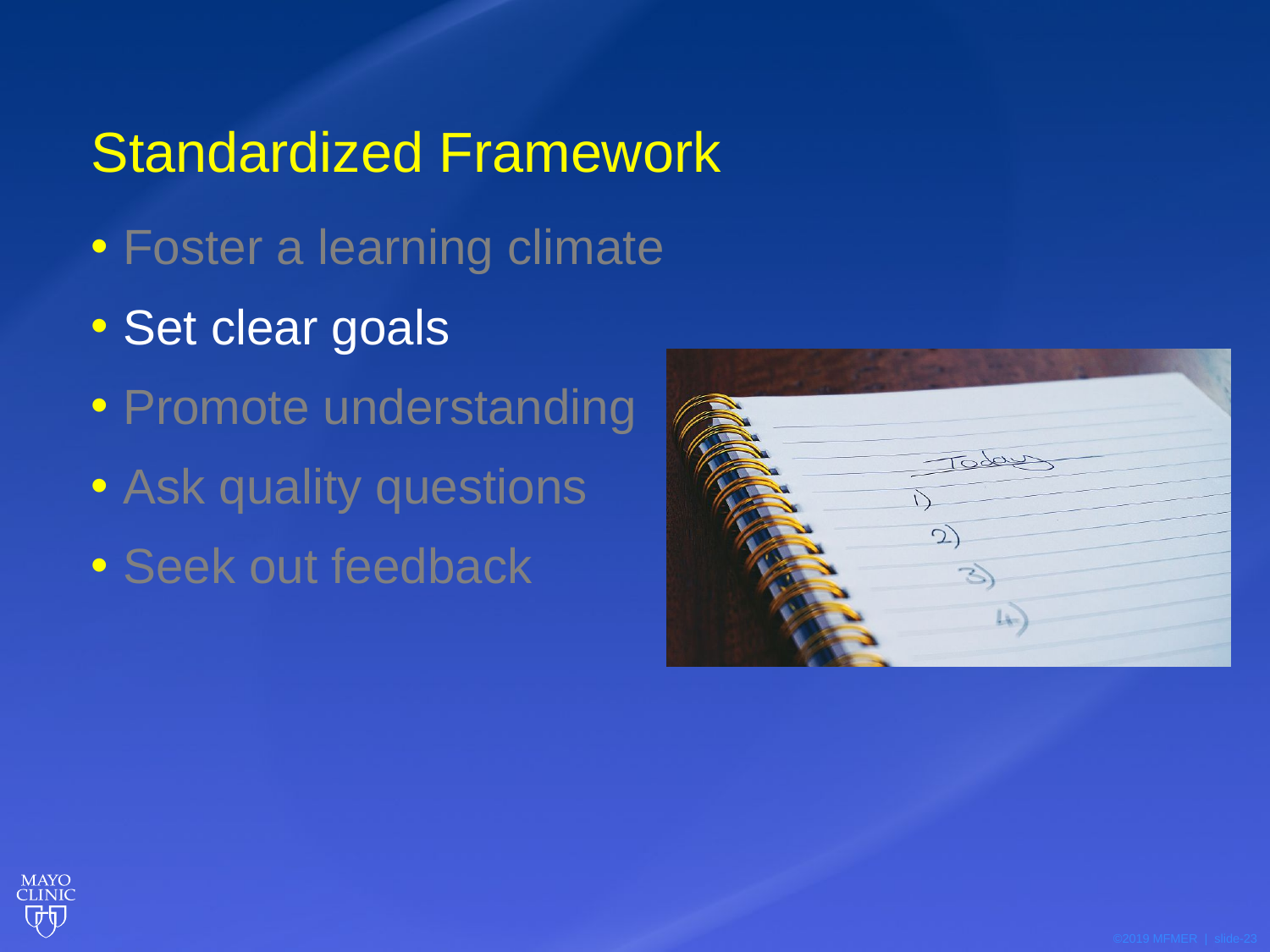

# Standardized Framework
Foster a learning climate
Set clear goals
Promote understanding
Ask quality questions
Seek out feedback

## Slide 24
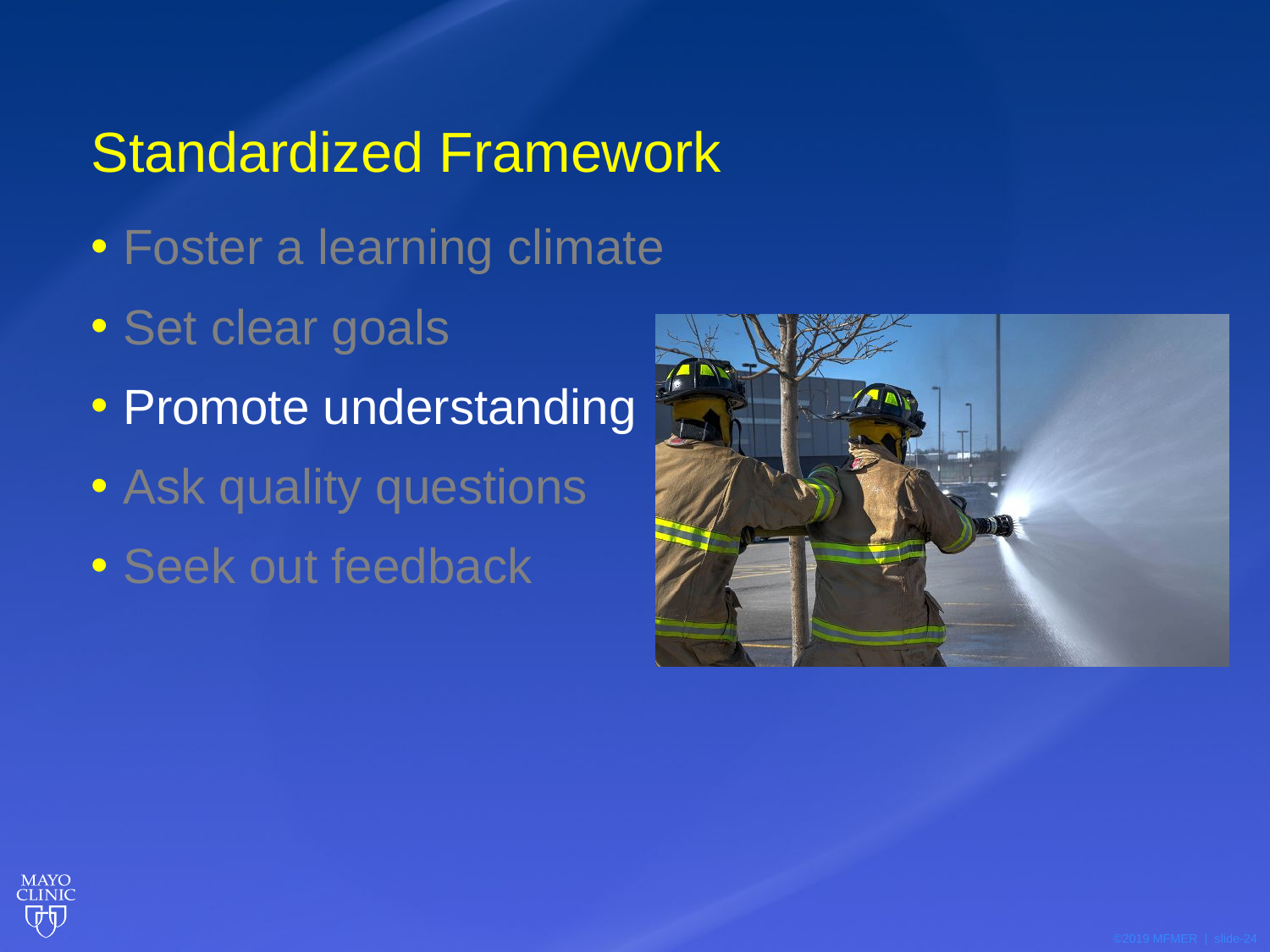

# Standardized Framework
Foster a learning climate
Set clear goals
Promote understanding
Ask quality questions
Seek out feedback

## Slide 25
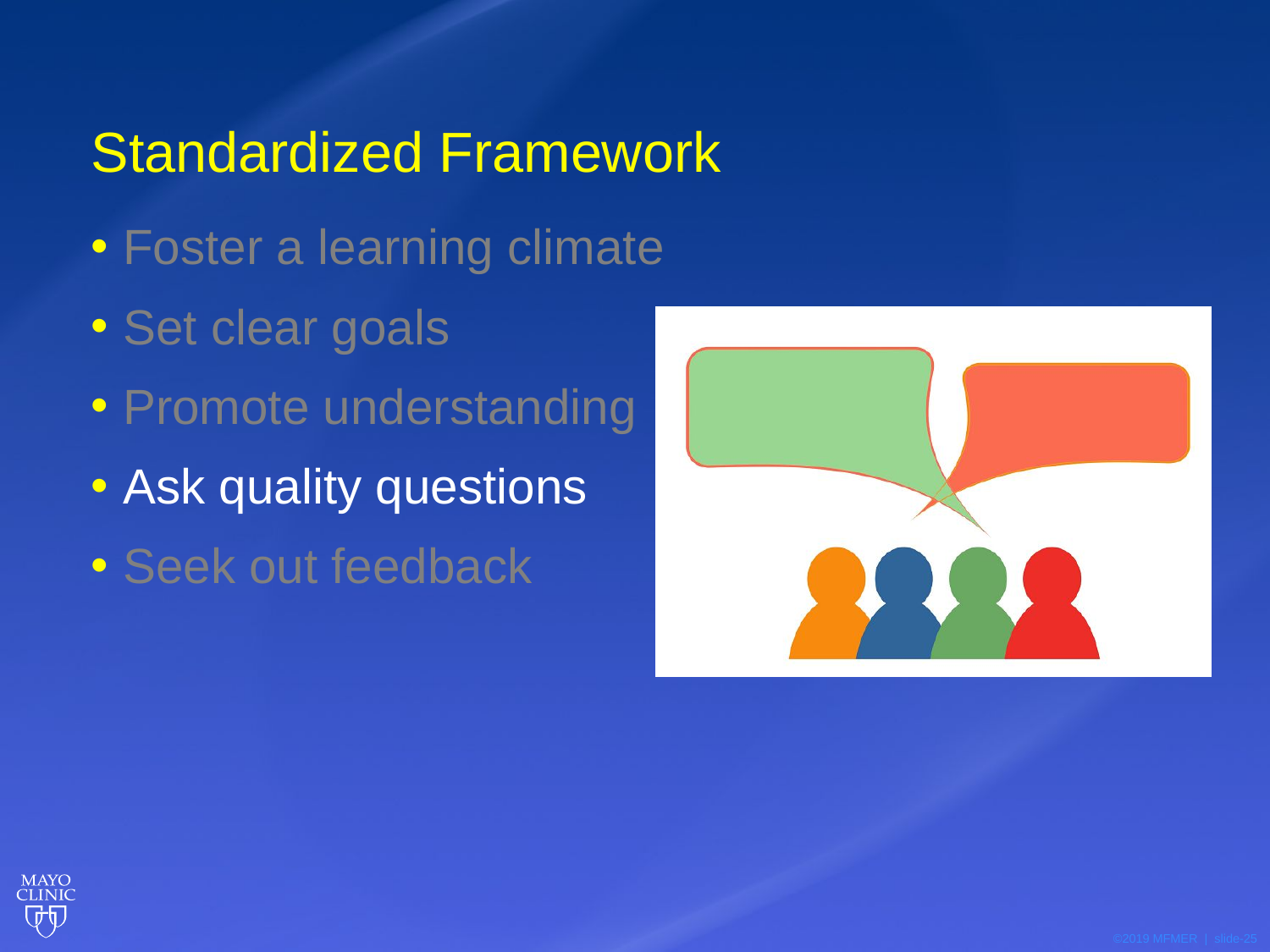

# Standardized Framework
Foster a learning climate
Set clear goals
Promote understanding
Ask quality questions
Seek out feedback

## Slide 26
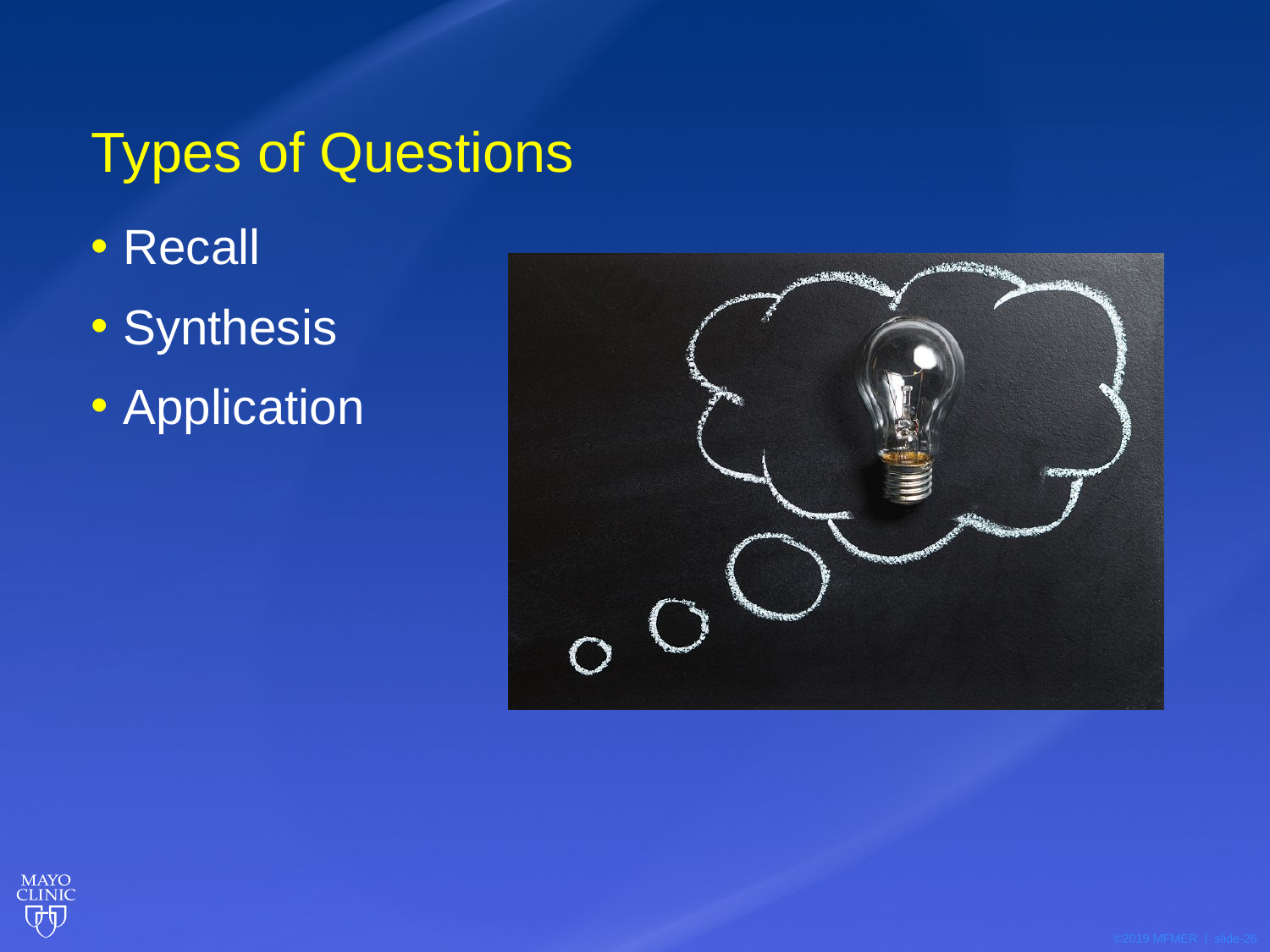

# Types of Questions
Recall
Synthesis
Application

## Slide 27
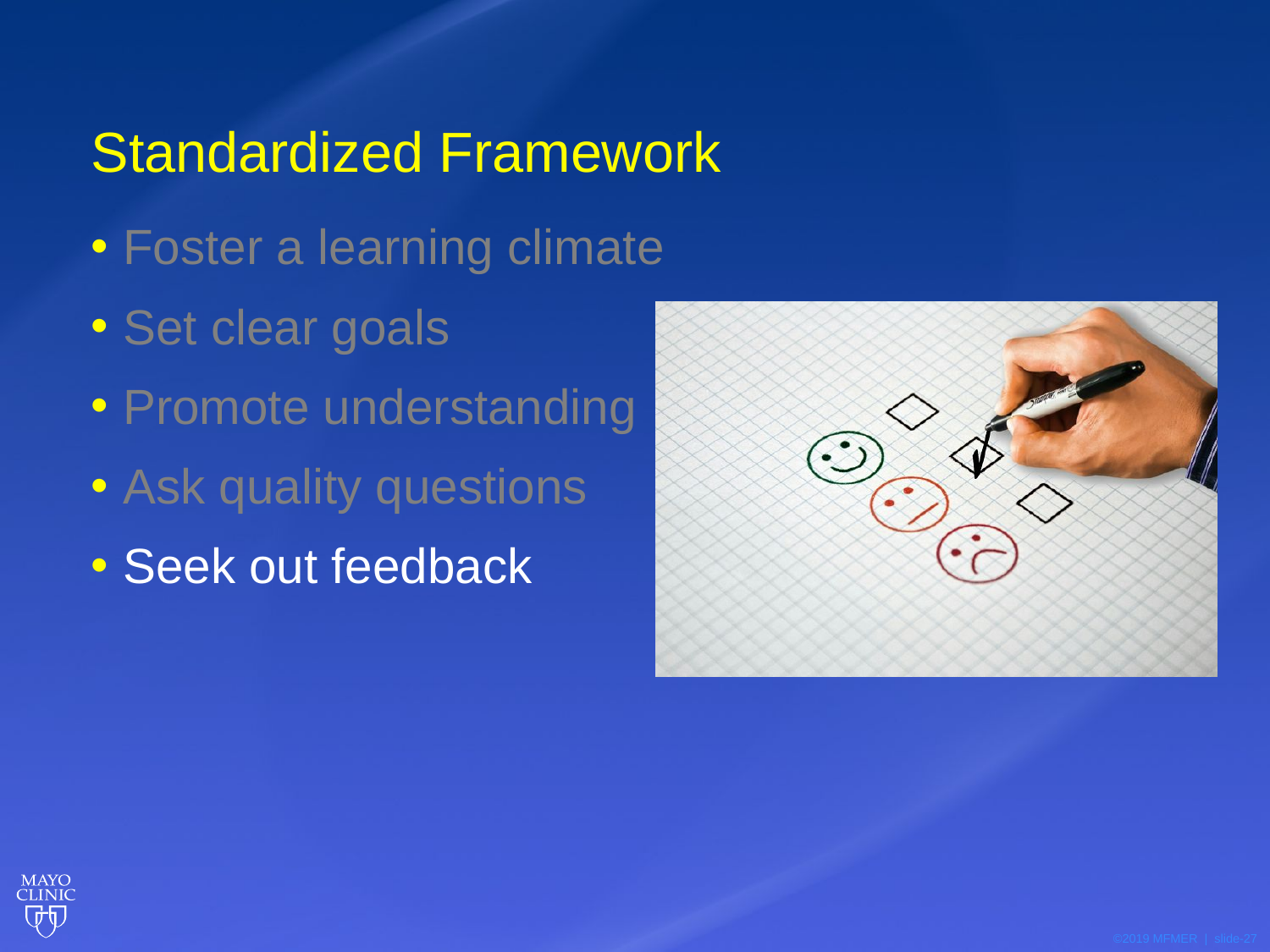

# Standardized Framework
Foster a learning climate
Set clear goals
Promote understanding
Ask quality questions
Seek out feedback

## Slide 28
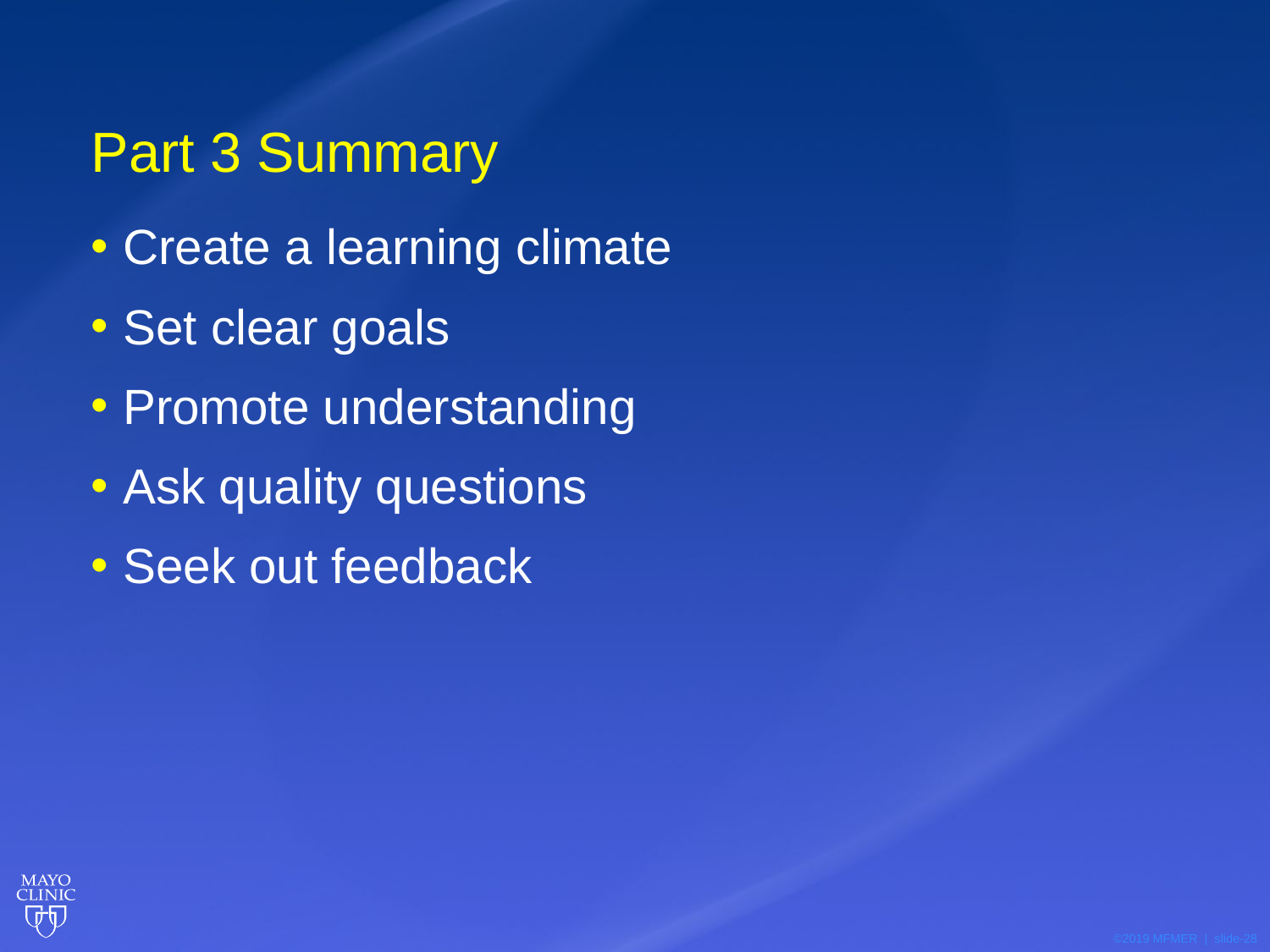

# Part 3 Summary
Create a learning climate
Set clear goals
Promote understanding
Ask quality questions
Seek out feedback

## Slide 29
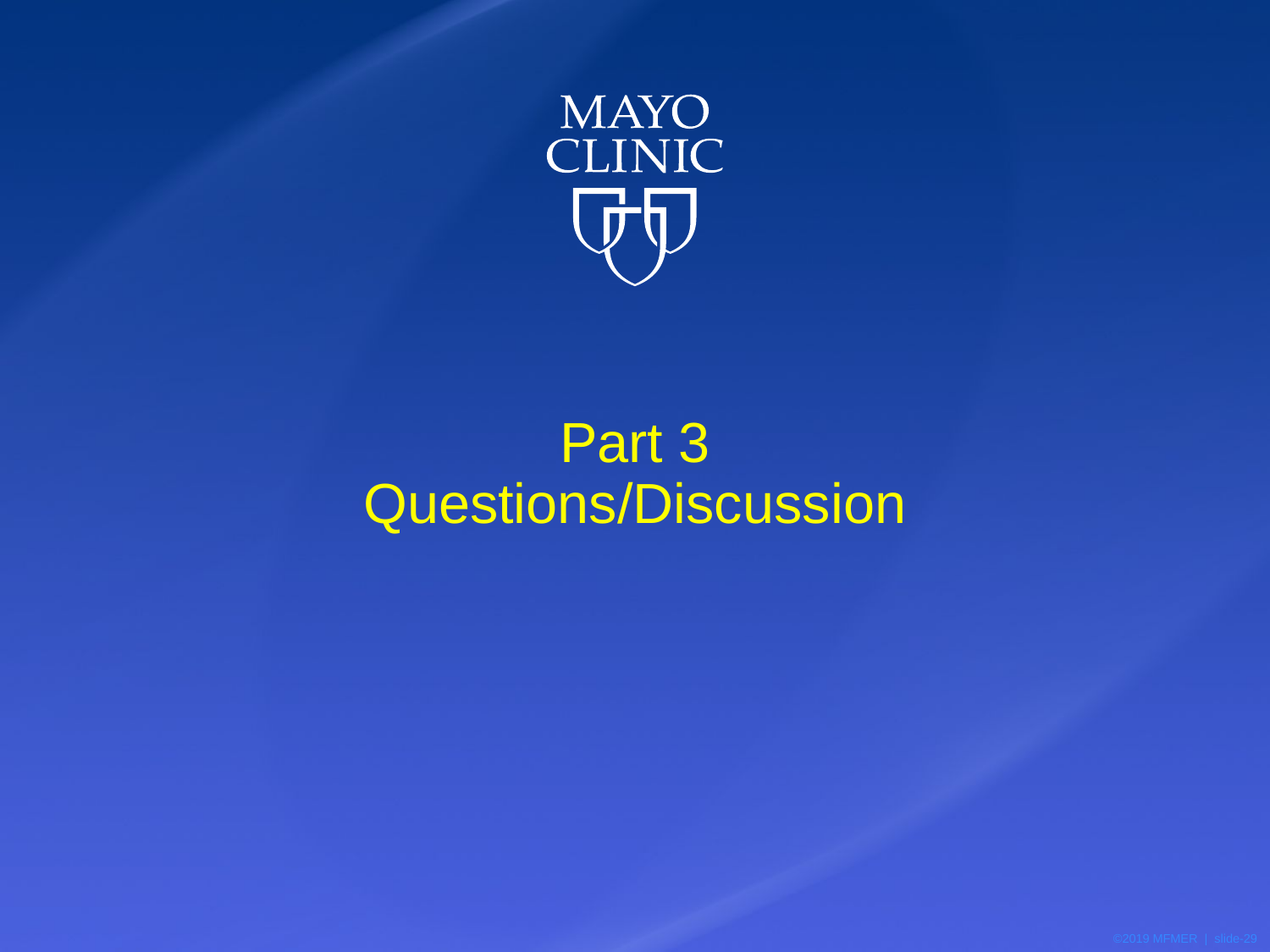

# Part 3 Questions/Discussion

## Slide 30
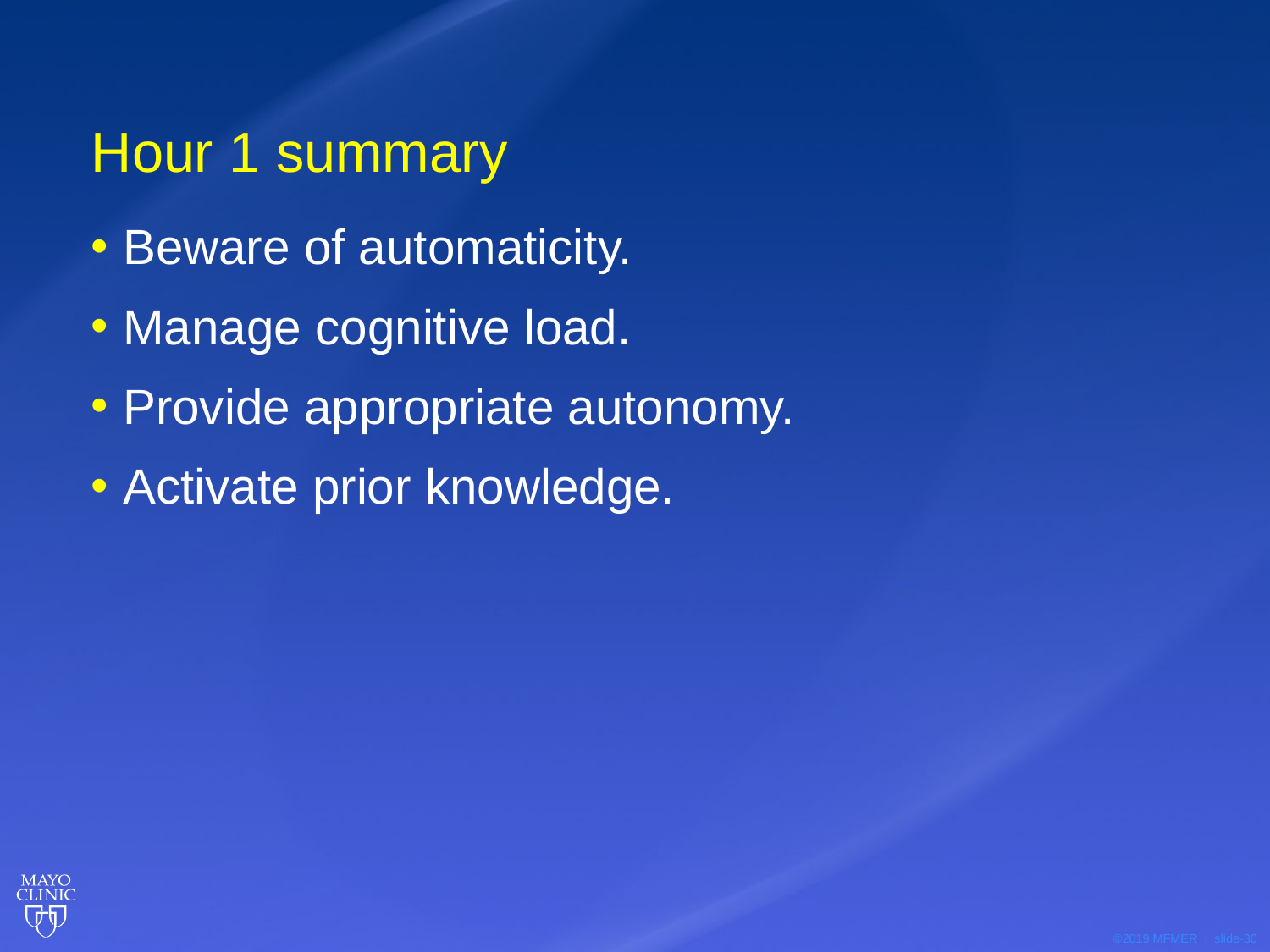

# Hour 1 summary
Beware of automaticity.
Manage cognitive load.
Provide appropriate autonomy.
Activate prior knowledge.

## Slide 31
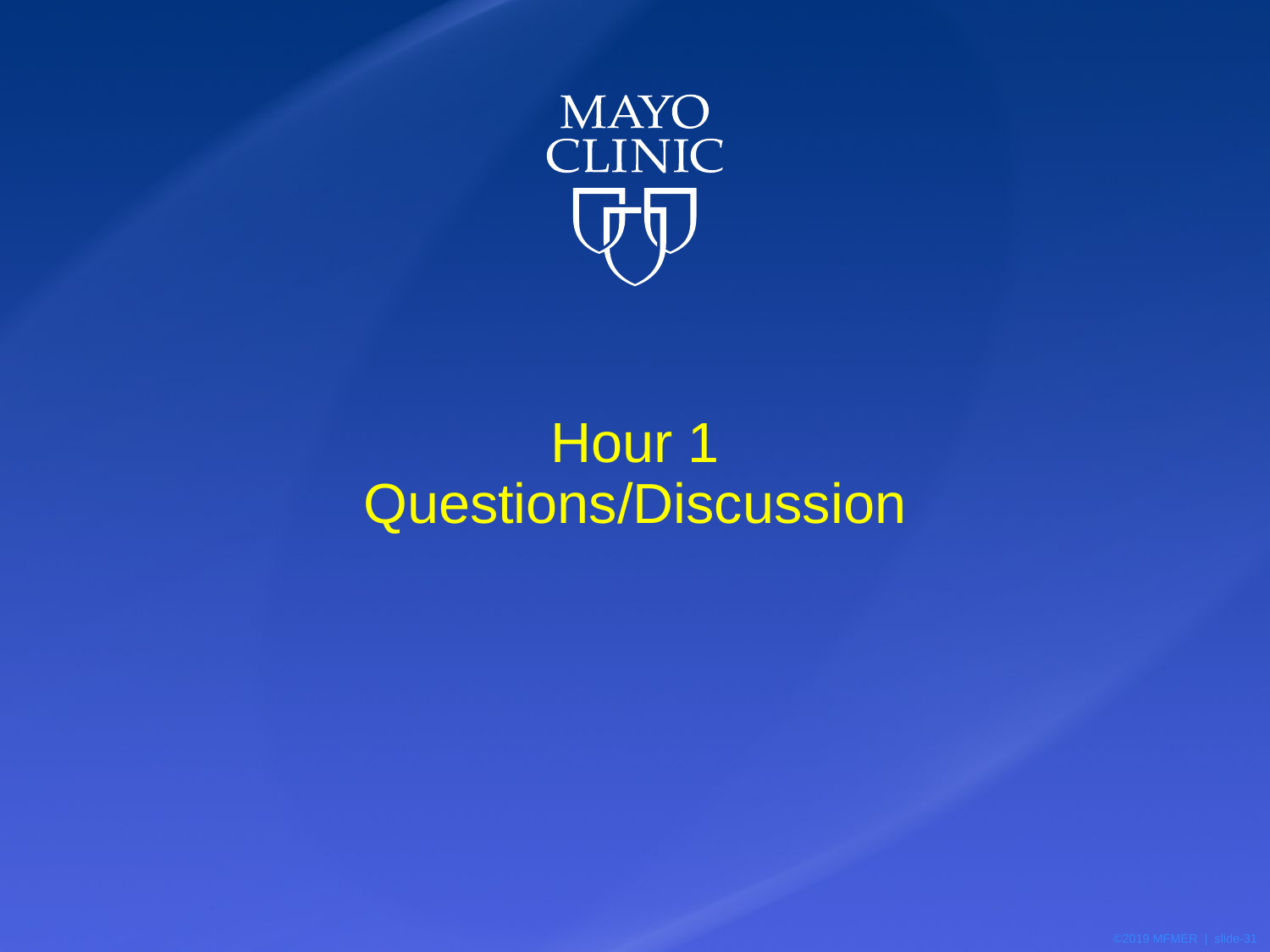

# Hour 1 Questions/Discussion

## Slide 32
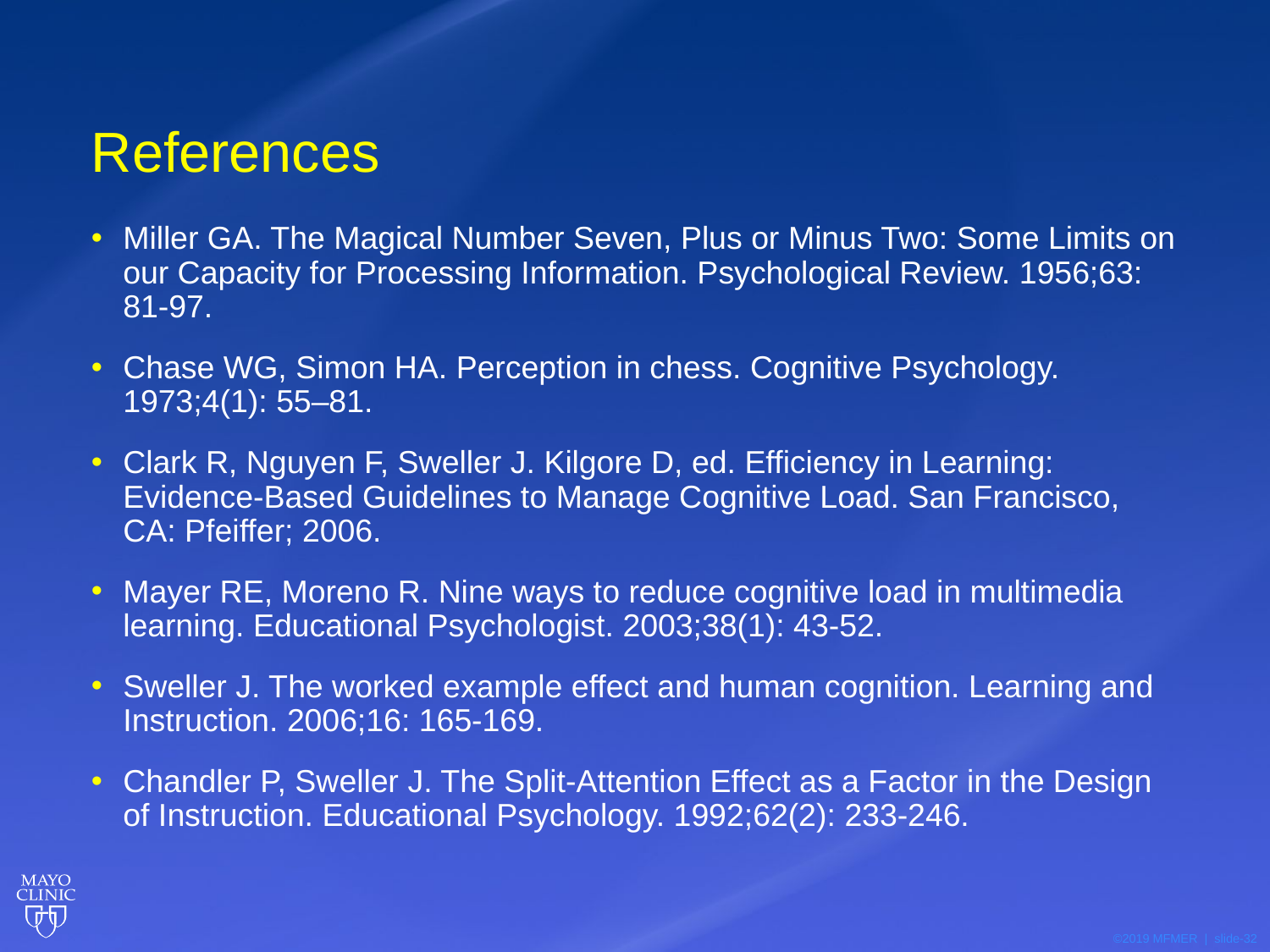

# References
Miller GA. The Magical Number Seven, Plus or Minus Two: Some Limits on our Capacity for Processing Information. Psychological Review. 1956;63: 81-97.
Chase WG, Simon HA. Perception in chess. Cognitive Psychology. 1973;4(1): 55–81.
Clark R, Nguyen F, Sweller J. Kilgore D, ed. Efficiency in Learning: Evidence-Based Guidelines to Manage Cognitive Load. San Francisco, CA: Pfeiffer; 2006.
Mayer RE, Moreno R. Nine ways to reduce cognitive load in multimedia learning. Educational Psychologist. 2003;38(1): 43-52.
Sweller J. The worked example effect and human cognition. Learning and Instruction. 2006;16: 165-169.
Chandler P, Sweller J. The Split-Attention Effect as a Factor in the Design of Instruction. Educational Psychology. 1992;62(2): 233-246.

## Slide 33
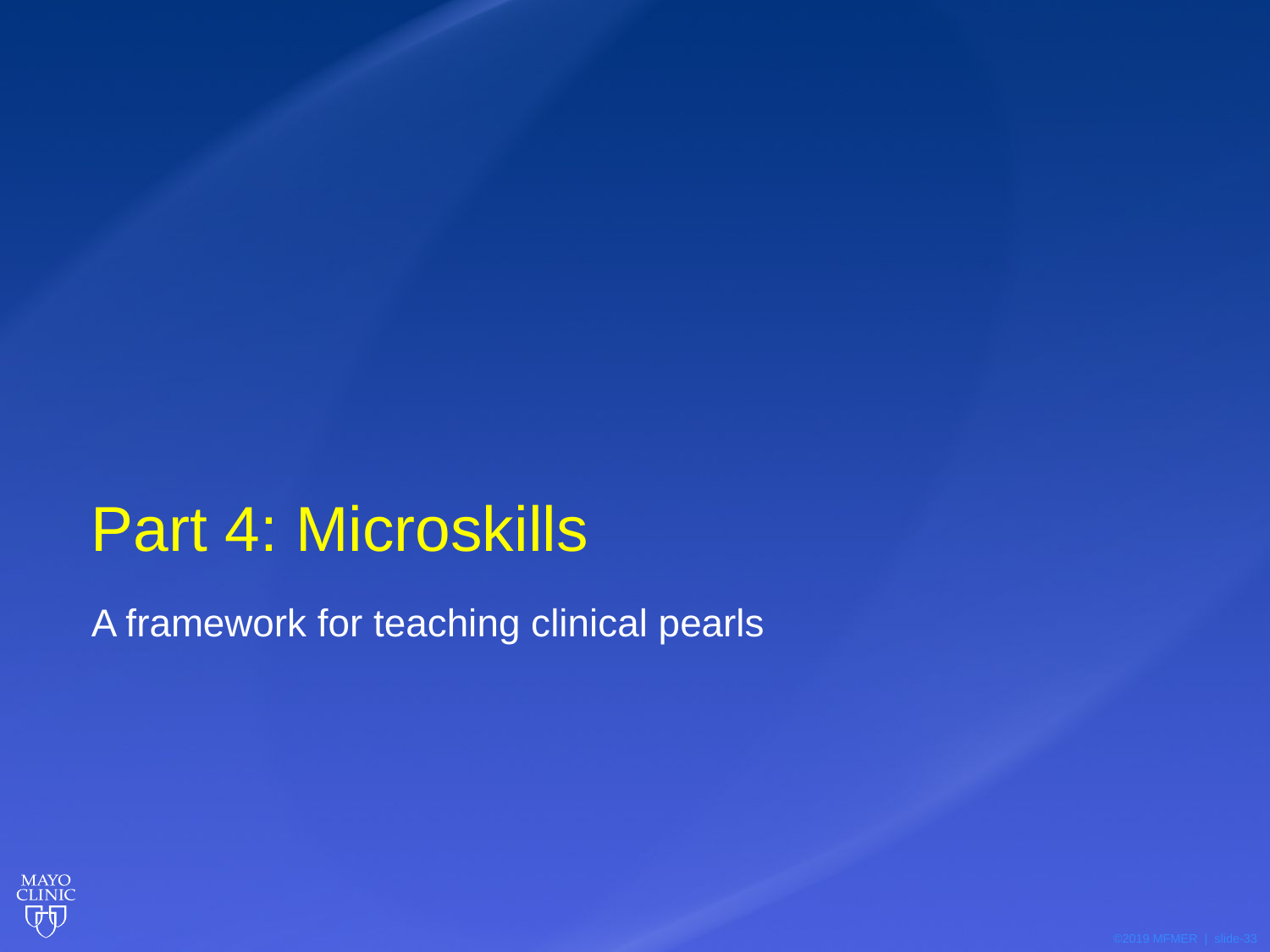

# Part 4: Microskills
A framework for teaching clinical pearls

## Slide 34
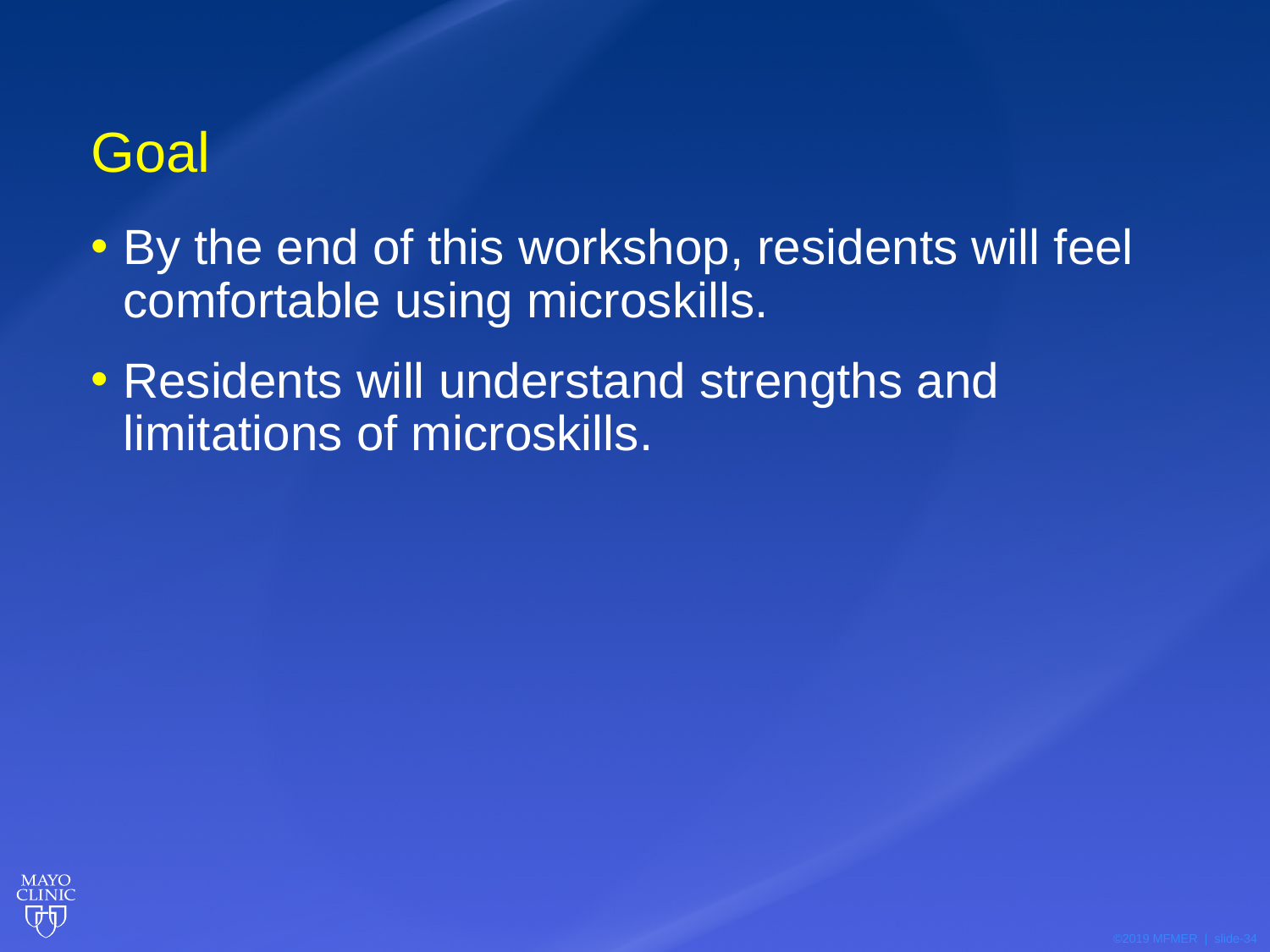

# Goal
By the end of this workshop, residents will feel comfortable using microskills.
Residents will understand strengths and limitations of microskills.

## Slide 35
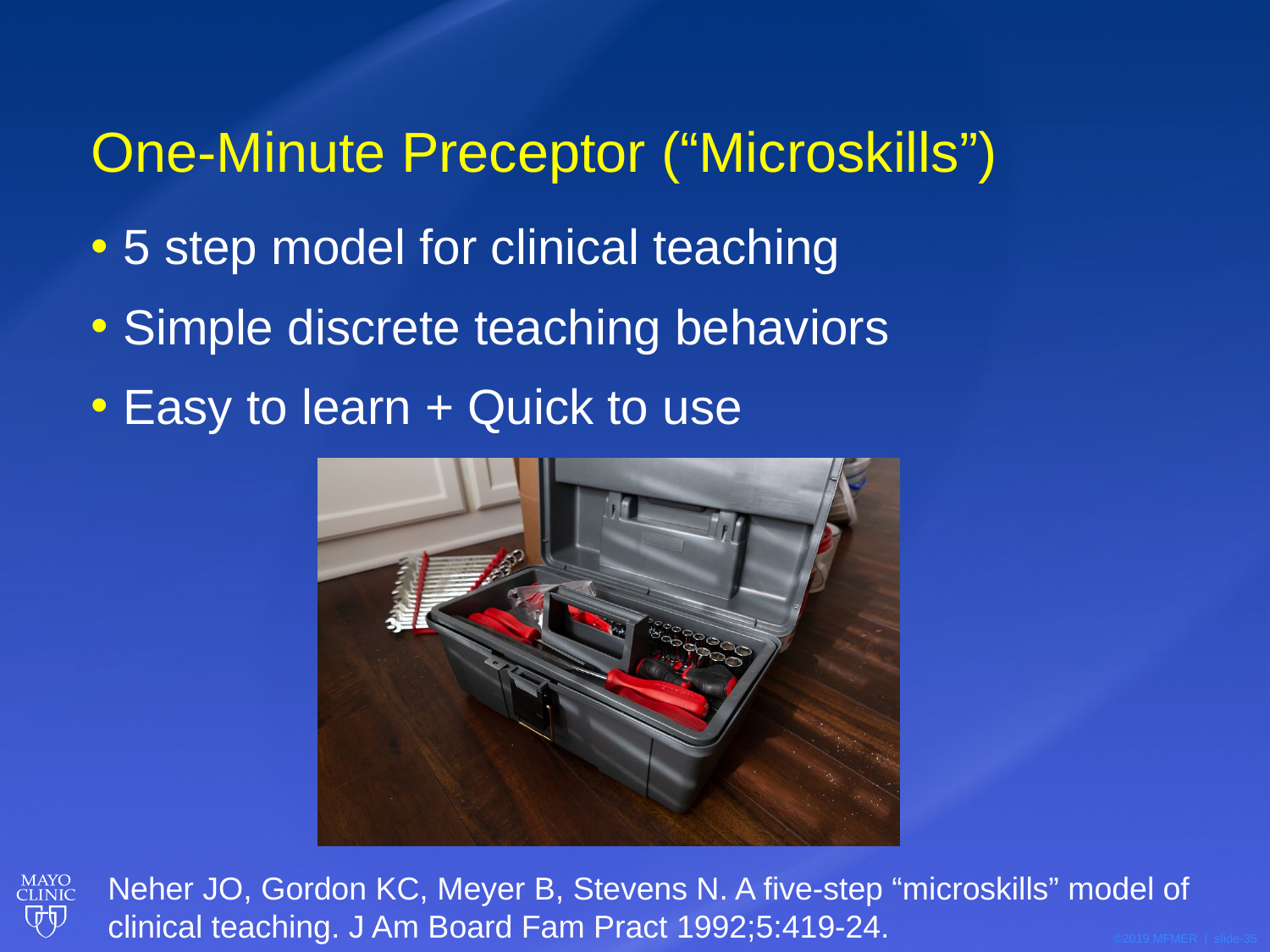

# One-Minute Preceptor (“Microskills”)
5 step model for clinical teaching
Simple discrete teaching behaviors
Easy to learn + Quick to use
Neher JO, Gordon KC, Meyer B, Stevens N. A five-step “microskills” model of clinical teaching. J Am Board Fam Pract 1992;5:419-24.

## Slide 36
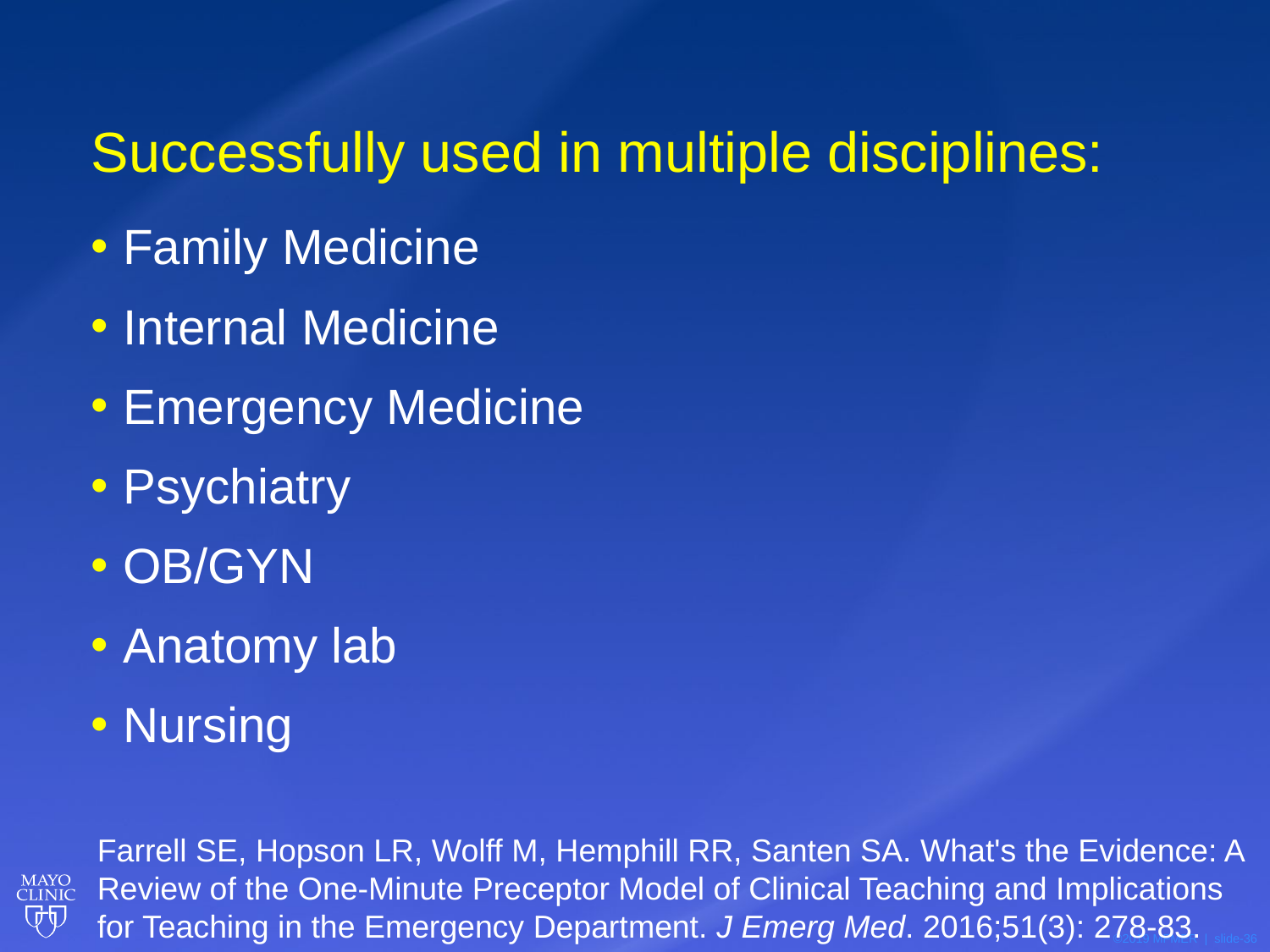

# Successfully used in multiple disciplines:
Family Medicine
Internal Medicine
Emergency Medicine
Psychiatry
OB/GYN
Anatomy lab
Nursing
Farrell SE, Hopson LR, Wolff M, Hemphill RR, Santen SA. What's the Evidence: A Review of the One-Minute Preceptor Model of Clinical Teaching and Implications for Teaching in the Emergency Department. J Emerg Med. 2016;51(3): 278-83.

## Slide 37
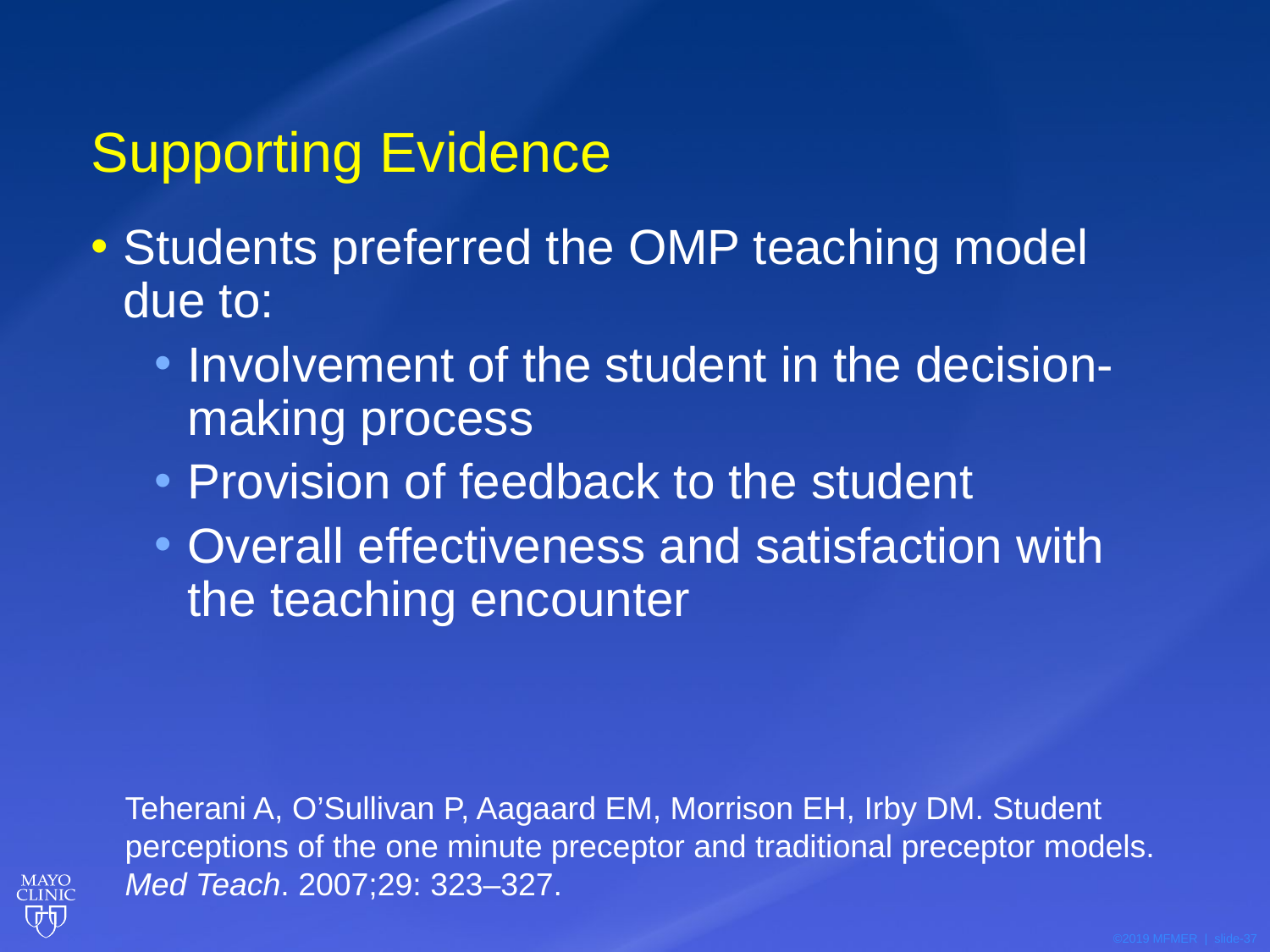

# Supporting Evidence
Students preferred the OMP teaching model due to:
Involvement of the student in the decision-making process
Provision of feedback to the student
Overall effectiveness and satisfaction with the teaching encounter
Teherani A, O’Sullivan P, Aagaard EM, Morrison EH, Irby DM. Student perceptions of the one minute preceptor and traditional preceptor models. Med Teach. 2007;29: 323–327.

## Slide 38
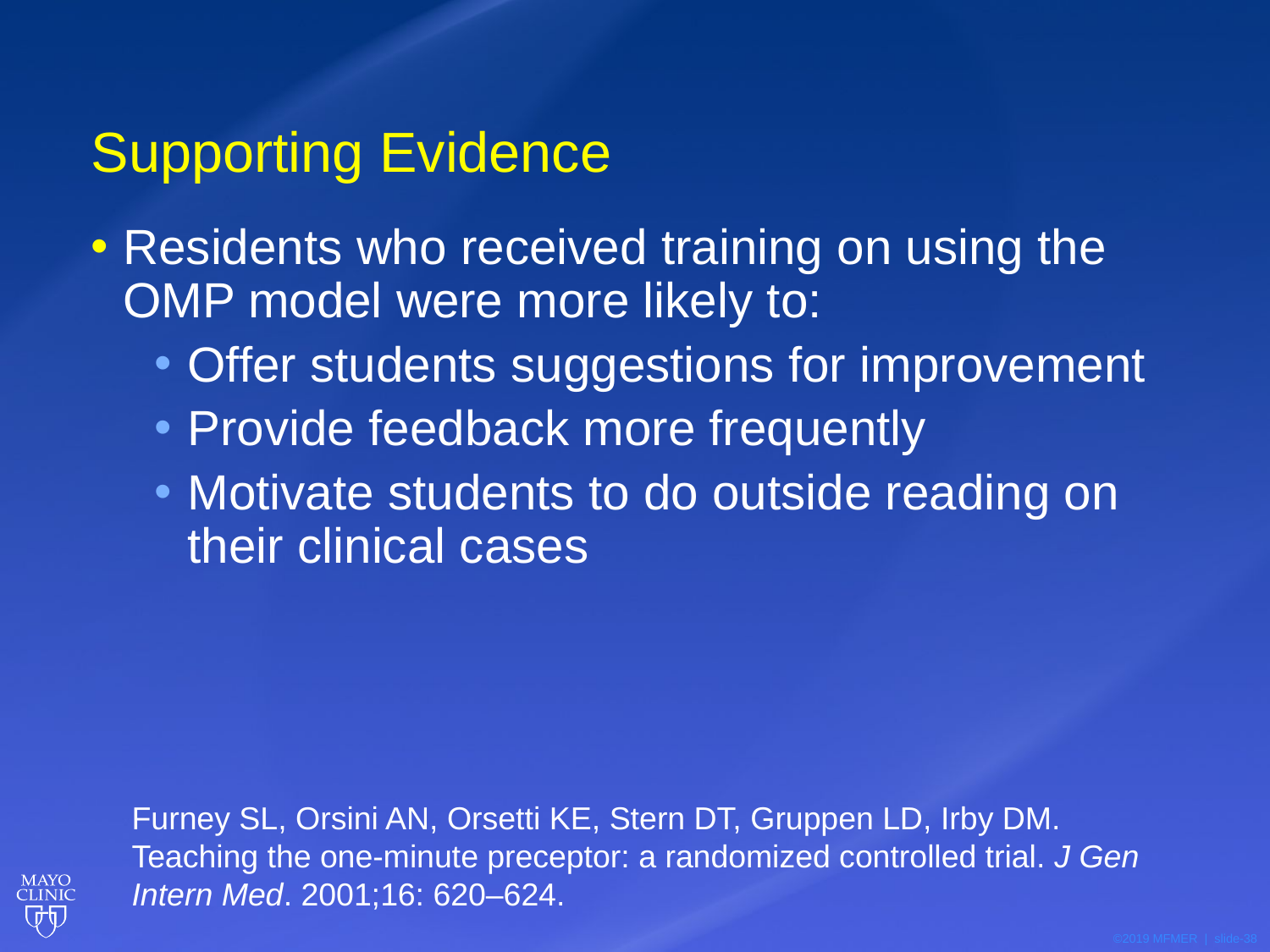

# Supporting Evidence
Residents who received training on using the OMP model were more likely to:
Offer students suggestions for improvement
Provide feedback more frequently
Motivate students to do outside reading on their clinical cases
Furney SL, Orsini AN, Orsetti KE, Stern DT, Gruppen LD, Irby DM. Teaching the one-minute preceptor: a randomized controlled trial. J Gen Intern Med. 2001;16: 620–624.

## Slide 39
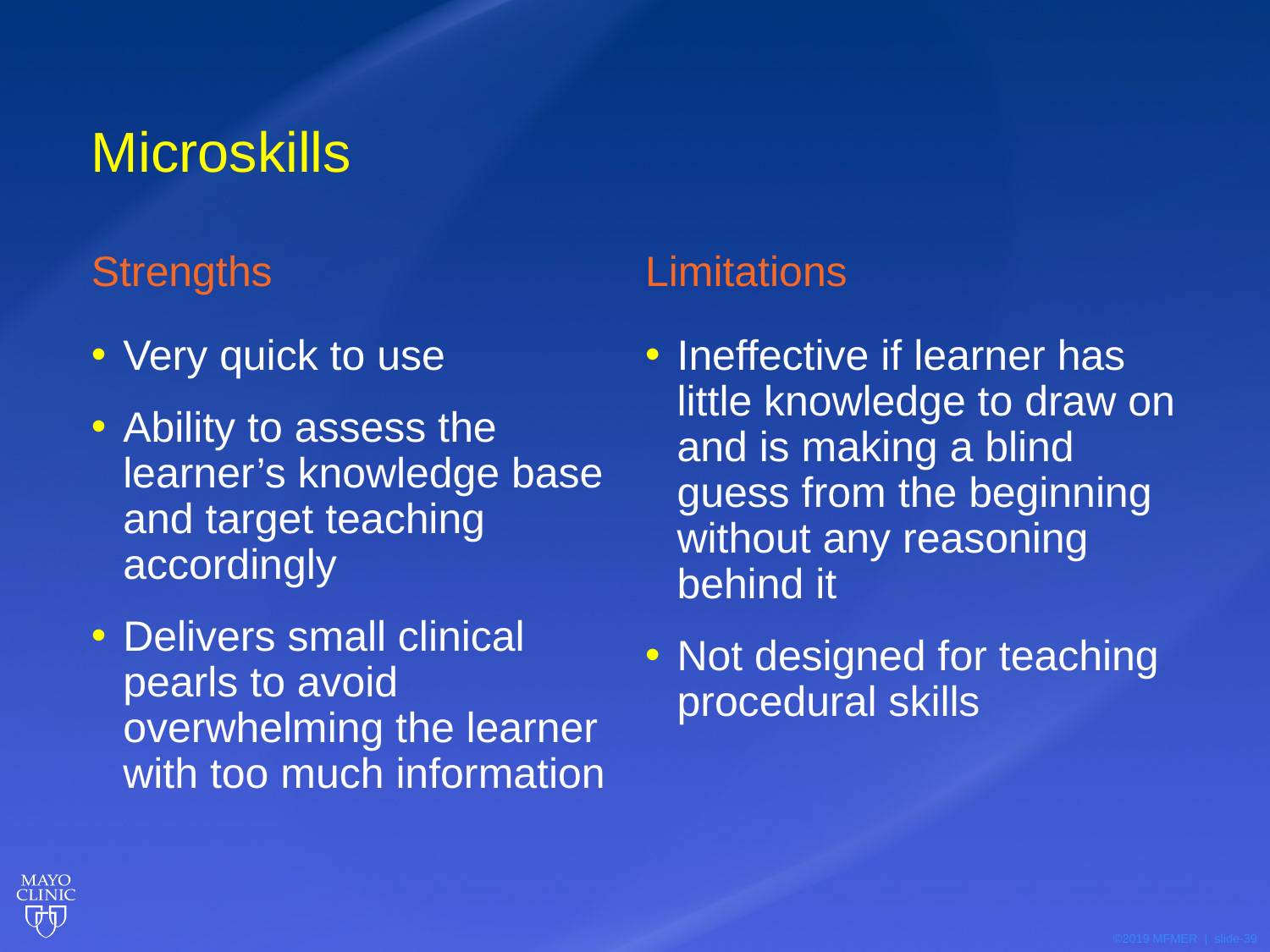

# Microskills
Strengths
Limitations
Very quick to use
Ability to assess the learner’s knowledge base and target teaching accordingly
Delivers small clinical pearls to avoid overwhelming the learner with too much information
Ineffective if learner has little knowledge to draw on and is making a blind guess from the beginning without any reasoning behind it
Not designed for teaching procedural skills

## Slide 40
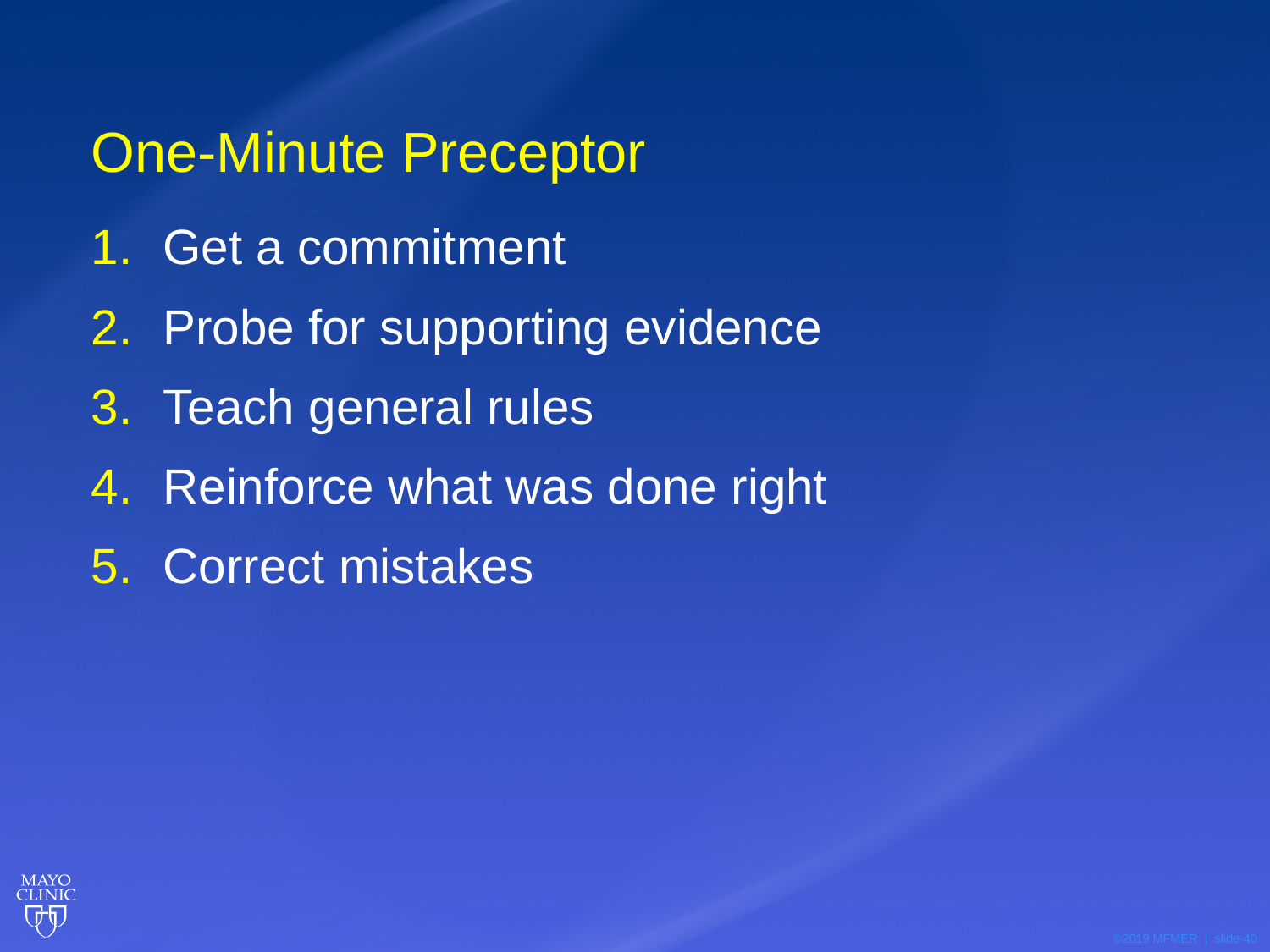

# One-Minute Preceptor
Get a commitment
Probe for supporting evidence
Teach general rules
Reinforce what was done right
Correct mistakes

## Slide 41
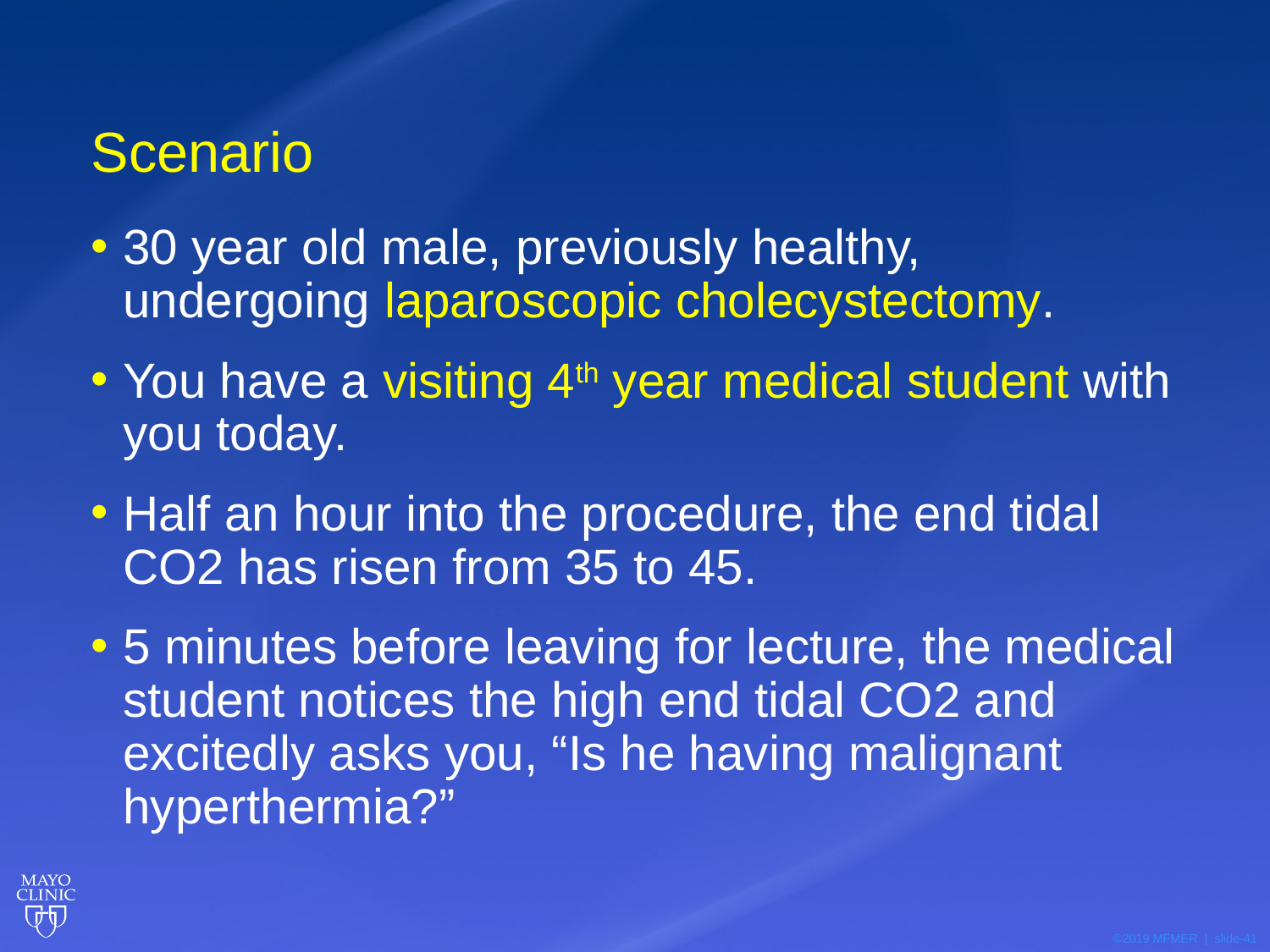

# Scenario
30 year old male, previously healthy, undergoing laparoscopic cholecystectomy.
You have a visiting 4th year medical student with you today.
Half an hour into the procedure, the end tidal CO2 has risen from 35 to 45.
5 minutes before leaving for lecture, the medical student notices the high end tidal CO2 and excitedly asks you, “Is he having malignant hyperthermia?”

## Slide 42
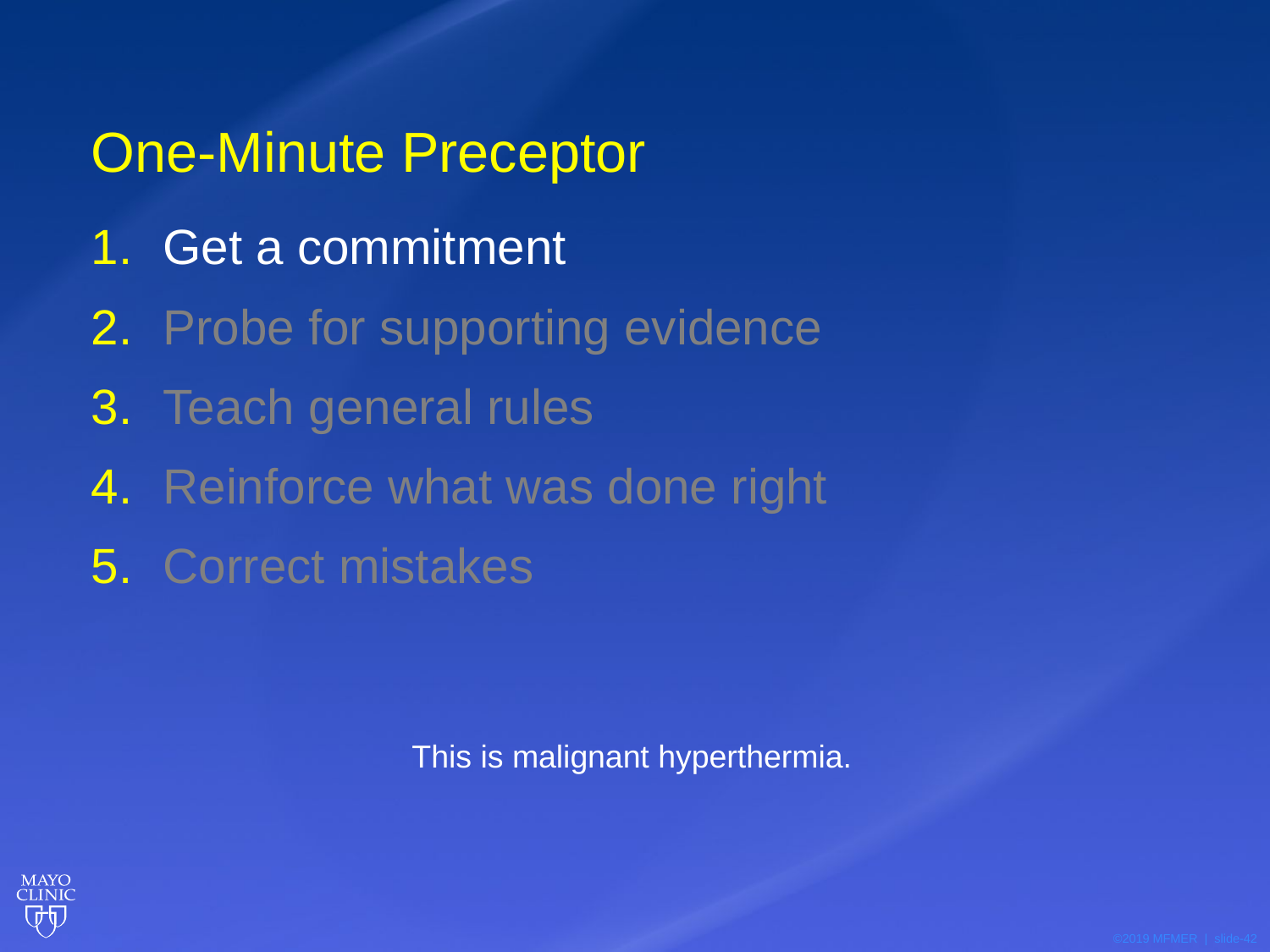

# One-Minute Preceptor
Get a commitment
Probe for supporting evidence
Teach general rules
Reinforce what was done right
Correct mistakes
This is malignant hyperthermia.

## Slide 43
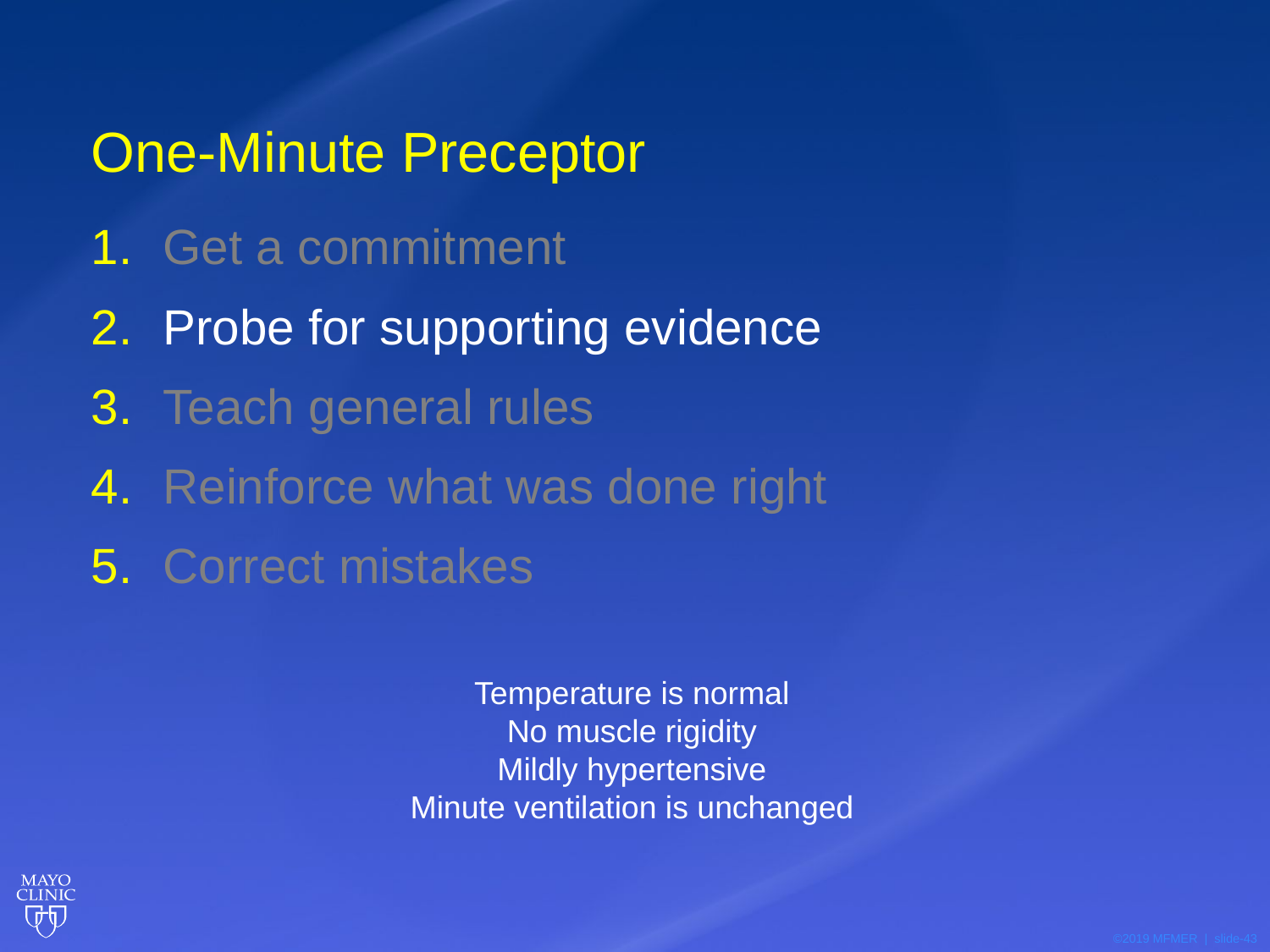

# One-Minute Preceptor
Get a commitment
Probe for supporting evidence
Teach general rules
Reinforce what was done right
Correct mistakes
Temperature is normal
No muscle rigidity
Mildly hypertensive
Minute ventilation is unchanged

## Slide 44
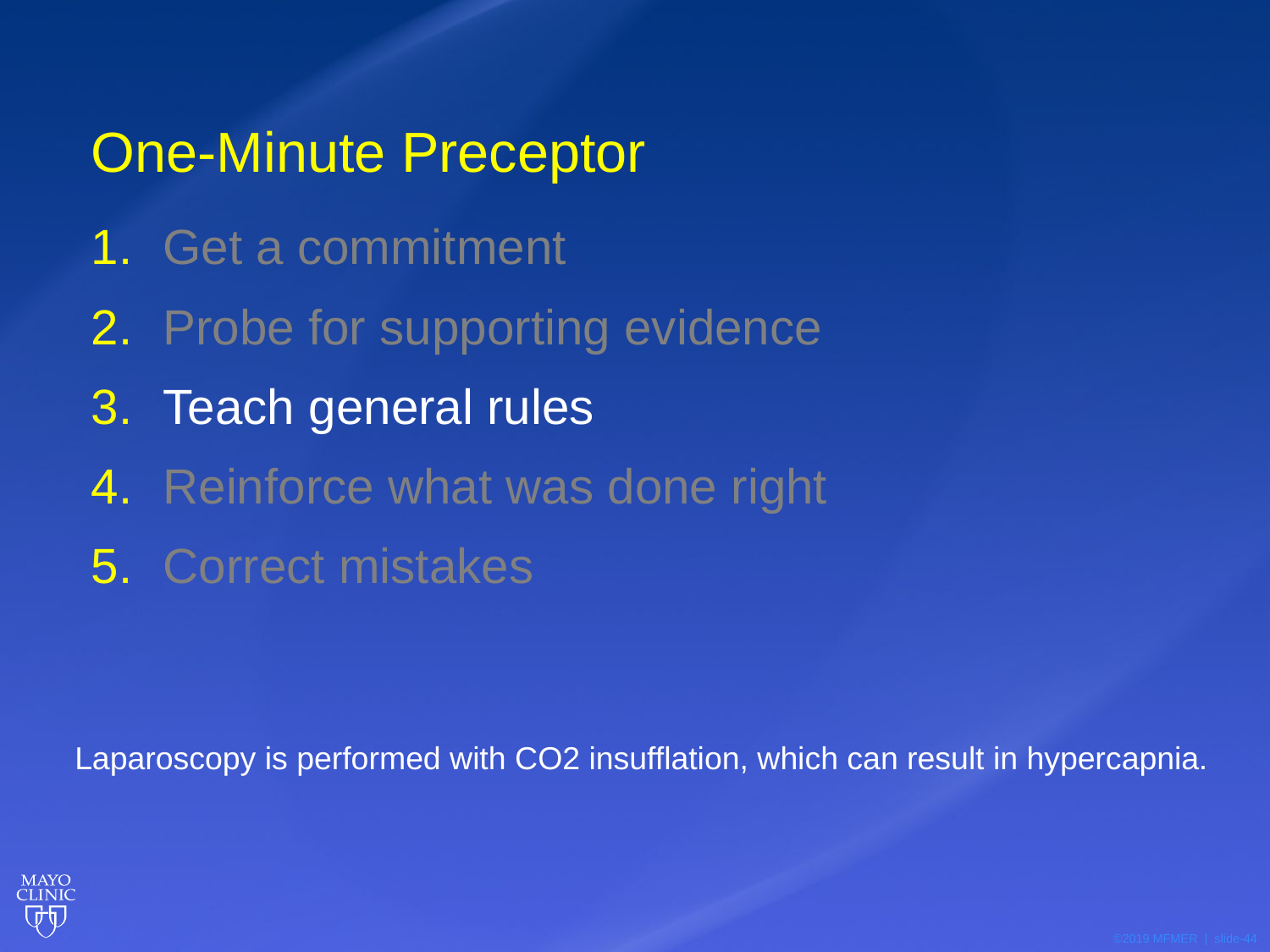

# One-Minute Preceptor
Get a commitment
Probe for supporting evidence
Teach general rules
Reinforce what was done right
Correct mistakes
Laparoscopy is performed with CO2 insufflation, which can result in hypercapnia.

## Slide 45
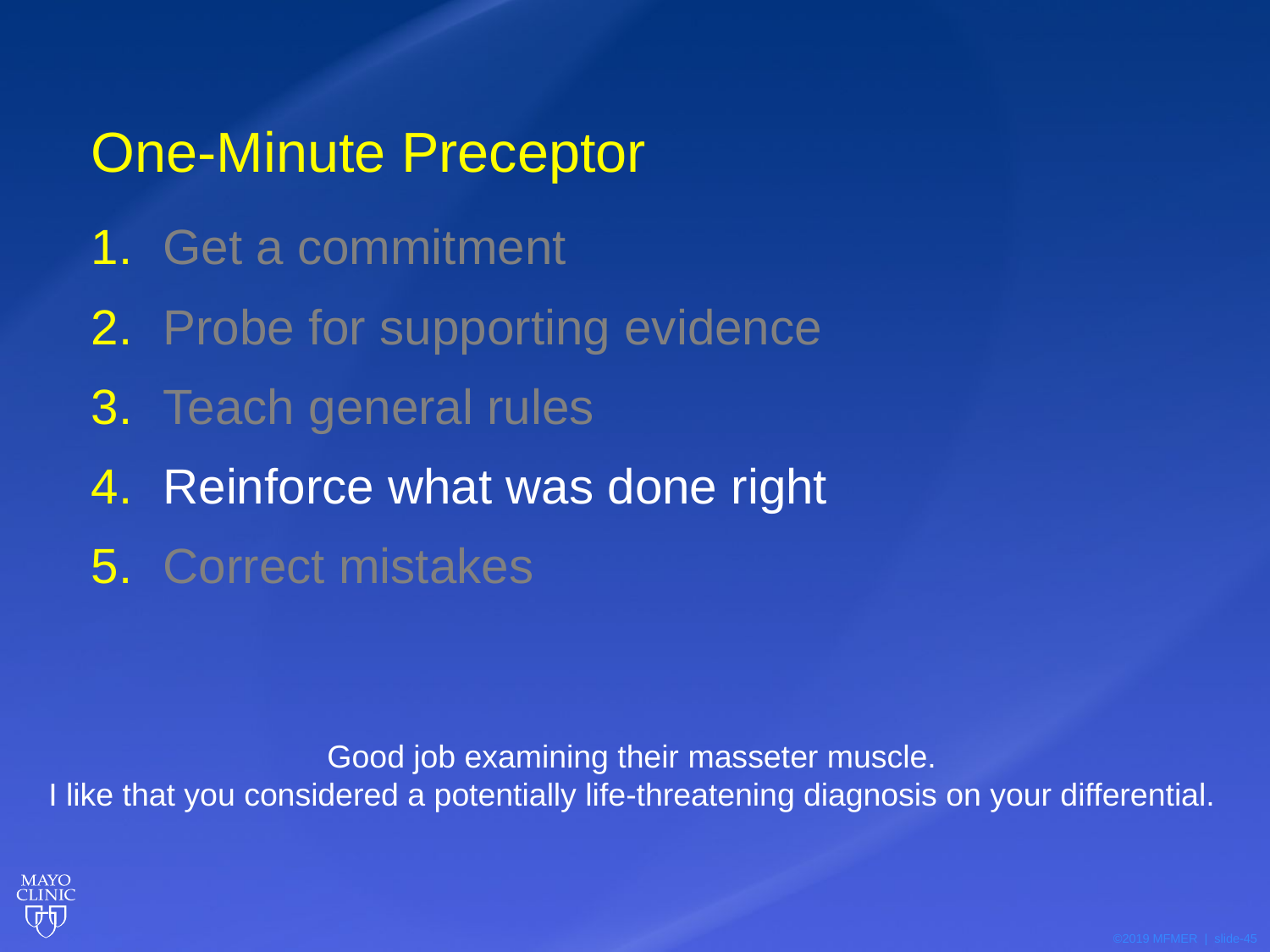

# One-Minute Preceptor
Get a commitment
Probe for supporting evidence
Teach general rules
Reinforce what was done right
Correct mistakes
Good job examining their masseter muscle.
I like that you considered a potentially life-threatening diagnosis on your differential.

## Slide 46
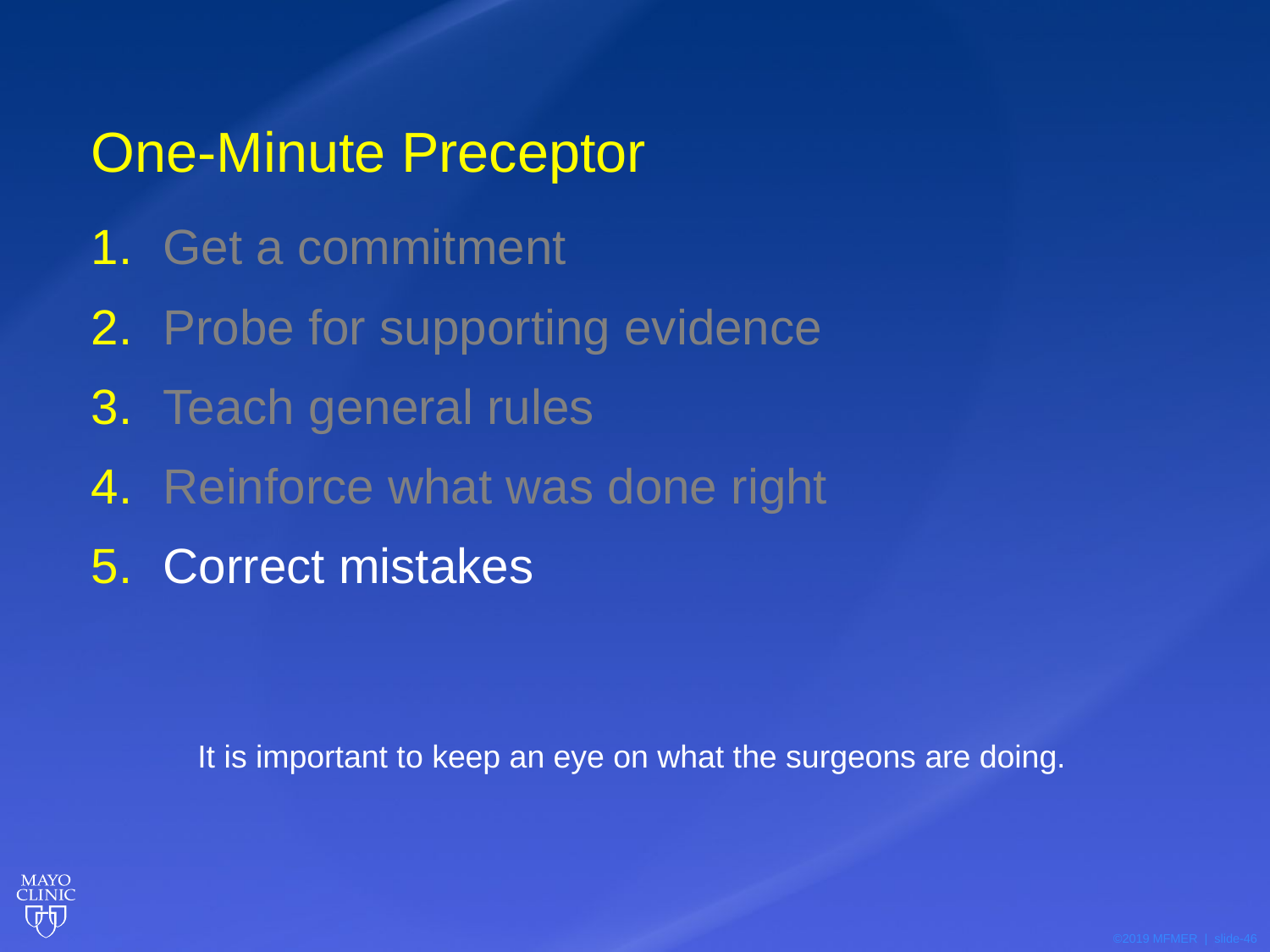

# One-Minute Preceptor
Get a commitment
Probe for supporting evidence
Teach general rules
Reinforce what was done right
Correct mistakes
It is important to keep an eye on what the surgeons are doing.

## Slide 47
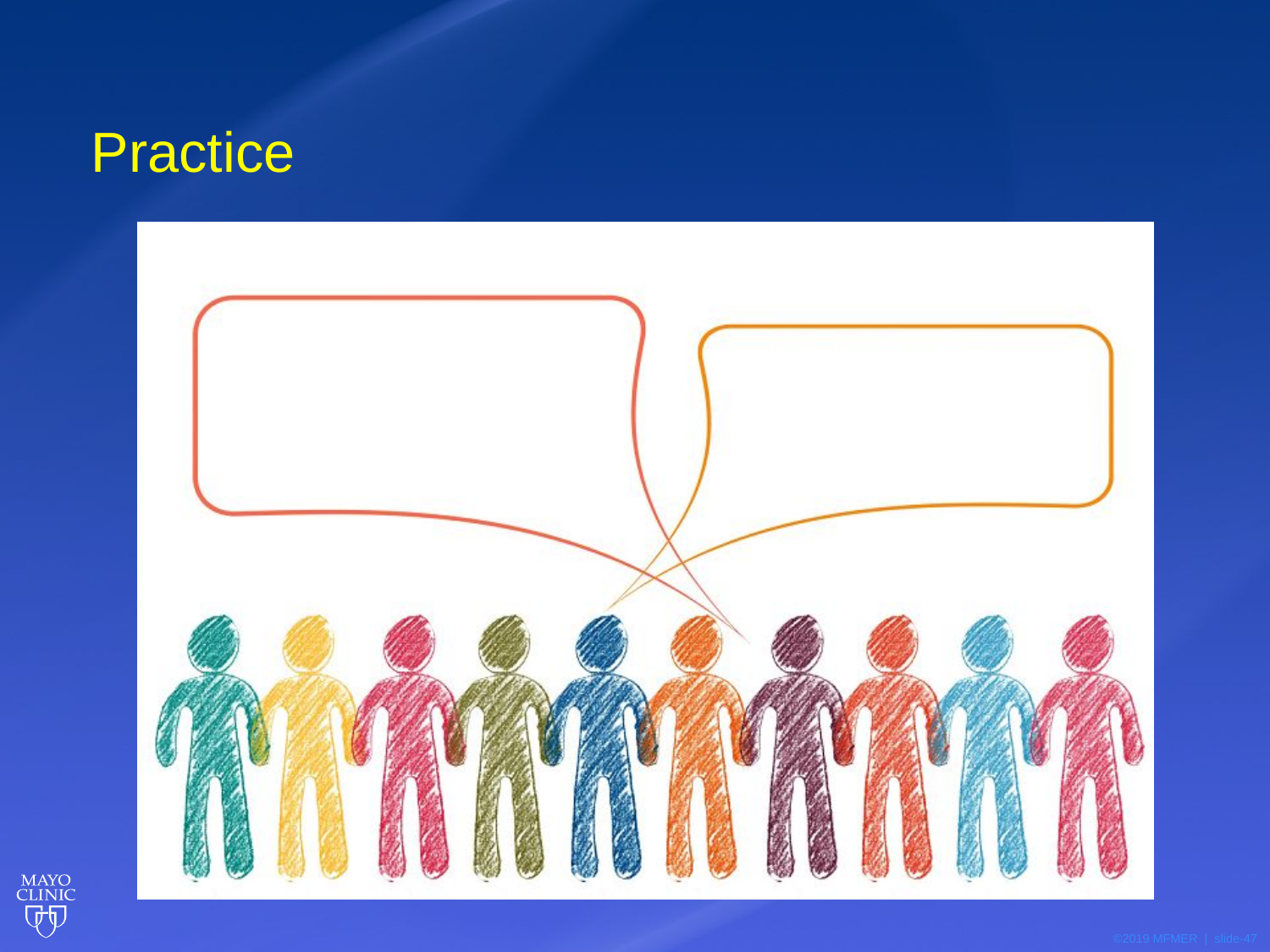

# Practice

## Slide 48
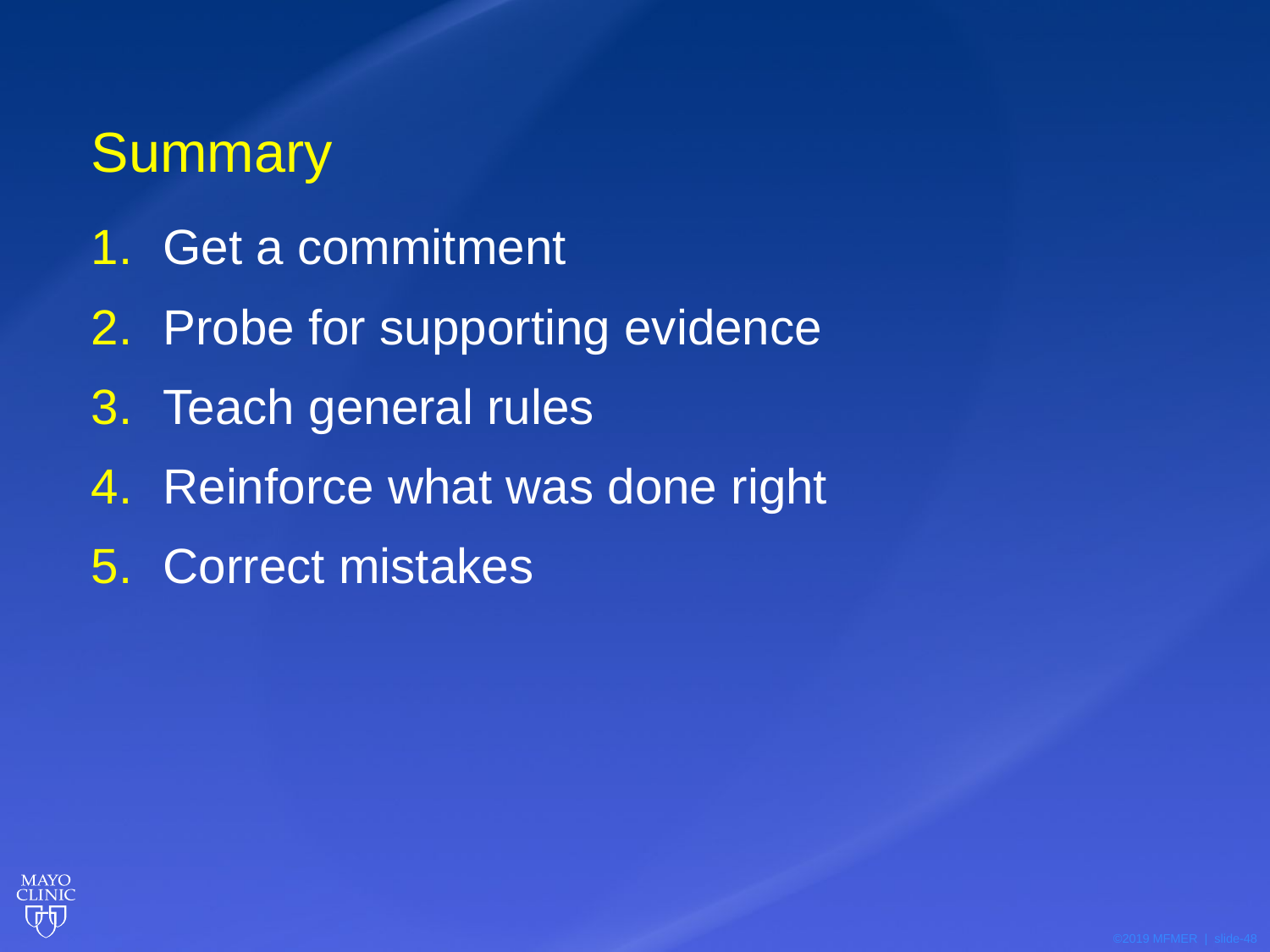

# Summary
Get a commitment
Probe for supporting evidence
Teach general rules
Reinforce what was done right
Correct mistakes

## Slide 49
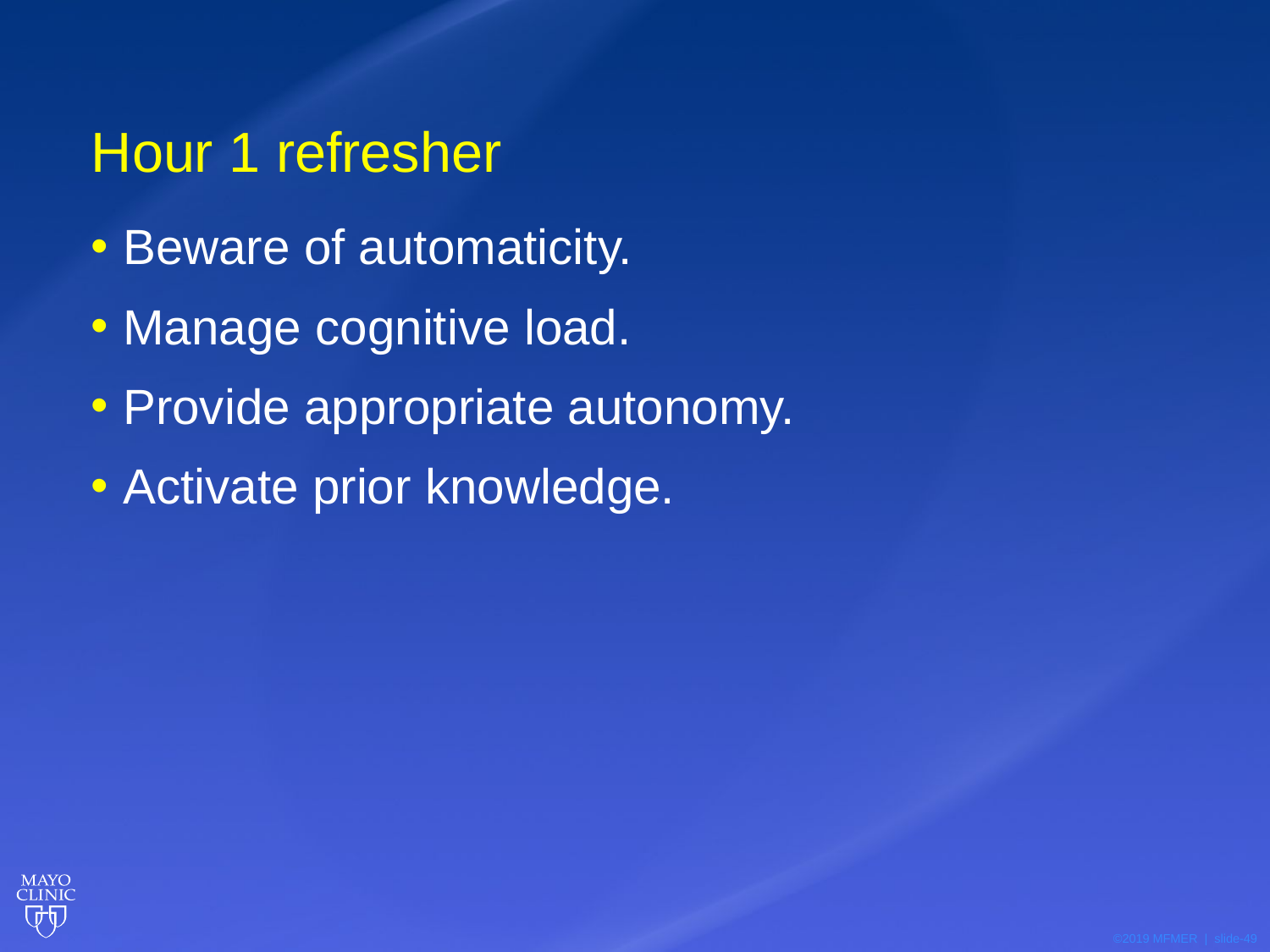

# Hour 1 refresher
Beware of automaticity.
Manage cognitive load.
Provide appropriate autonomy.
Activate prior knowledge.

## Slide 50
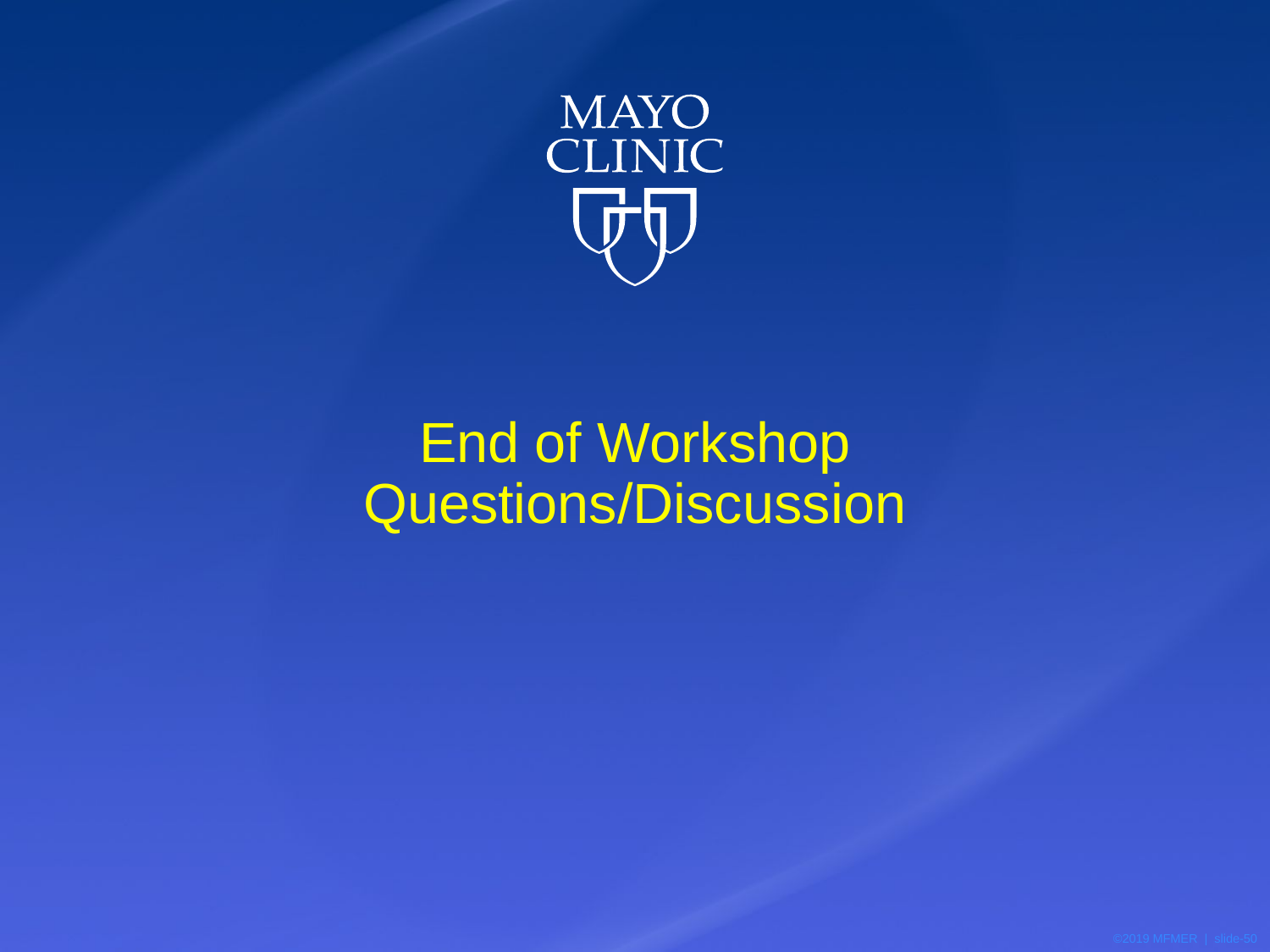

# End of Workshop Questions/Discussion

## Slide 51
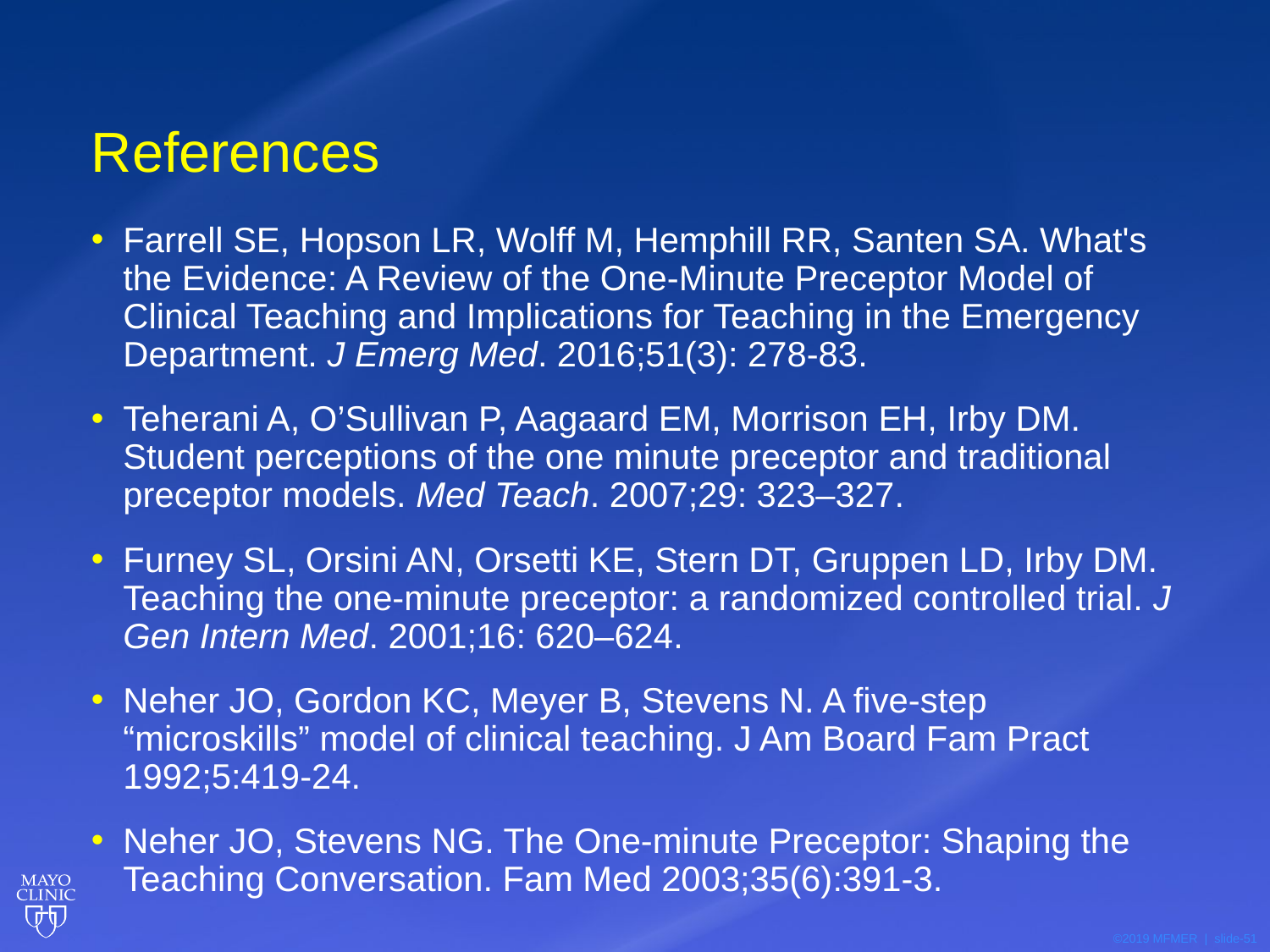

# References
Farrell SE, Hopson LR, Wolff M, Hemphill RR, Santen SA. What's the Evidence: A Review of the One-Minute Preceptor Model of Clinical Teaching and Implications for Teaching in the Emergency Department. J Emerg Med. 2016;51(3): 278-83.
Teherani A, O’Sullivan P, Aagaard EM, Morrison EH, Irby DM. Student perceptions of the one minute preceptor and traditional preceptor models. Med Teach. 2007;29: 323–327.
Furney SL, Orsini AN, Orsetti KE, Stern DT, Gruppen LD, Irby DM. Teaching the one-minute preceptor: a randomized controlled trial. J Gen Intern Med. 2001;16: 620–624.
Neher JO, Gordon KC, Meyer B, Stevens N. A five-step “microskills” model of clinical teaching. J Am Board Fam Pract 1992;5:419-24.
Neher JO, Stevens NG. The One-minute Preceptor: Shaping the Teaching Conversation. Fam Med 2003;35(6):391-3.
