# Supplementary material for: Training the Trainer: Preparing Anesthesiology Residents to be Trainers in the Operating Room
Source: MedEdPORTAL. 2021 Mar 4;17:11116. doi: 10.15766/mep_2374-8265.11116 (PMC7970634; doi:10.15766/mep_2374-8265.11116)
Supplement: Supplementary file 1 — Primer Document.docxWorkshop Handout.docxWorkshop PowerPoint.pptxInstructor Manual.docxPresurvey.pdfPostsurvey.pdf1-Week Follow-up Survey.docx1-Month Follow-up Survey.docxNew CA 1 Survey.docx [file mep_2374-8265.11116-s001.zip › D. Instructor Manual.docx]

Appendix D: Instructor Manual

Preparation:

This workshop is intended to be administered over the span of two hours. See Appendix C for the associated PowerPoint slides.

One week prior to workshop:

Distribute the **primer document (Appendix A)** to residents and ask them to review the document prior to the workshop.

Day of workshop:

Distribute the **workshop handout (Appendix B)** to all residents. See below for slide notes for the PowerPoint presentation. Small group breakout sessions will occur throughout the presentation and the details are described below in the slide notes.

Optional: **Appendix E (Pre Survey)** can be administered to all residents before starting the presentation, to assess baseline resident knowledge.

Slide 1:

*(Notes to presenter: The small group sessions are designed to be open book. During small group sessions, residents should be encouraged to refer to the primer document and make full use of the internet/smart devices to help answer the questions. The examples provided in the speaker notes throughout this presentation are geared towards teaching new CA-1 residents. However, the concepts still apply for teaching medical students and other learners. Examples involving medical students may be used instead if desired)*

**[Make sure residents are seated in such a way that they will be able to break into small groups of 2-3 residents throughout this talk]**

Welcome everyone.

**[Introduce self]**

During this workshop, we will cover various concepts from learning theory to help make you a more effective trainer.

Think back to when you were a medical student rotating on the wards. There were times when you were learning a lot and it felt great. And there were also experiences that were less good.

Slide 2:

The goal is that by the end of this workshop, you will acquire the knowledge and the skills so that you too can brighten someone’s day. And remember, quality teaching benefits both the student and the teacher. You may have heard of the phrase “See one, do one, teach one.” Teaching is a fantastic way to solidify concepts in your head. And it’s a great way to see how far you yourself have come and maybe even identify areas of improvement for yourself.

Slide 3:

During the first hour, we will focus on the first three items here: cognitive load theory, general principles of effective teaching, and a framework for evaluating the quality of teaching. During the second hour, we will discuss and practice microskills.

Slide 4:

We’ll start with cognitive load theory – how to avoid drowning your learner with too much information at once.

Slide 5:

Cognitive load theory has been around for several decades. One of the key works was Chase and Simon in 1973 who discussed the idea of schema construction in the setting of the board game chess. Whereas beginners in chess perceive the board as individual pieces (pawn, knight, rook, etc), masters recognize various formations of multiple pieces in a particular configuration as a single unit – a schema or chunk if you will. This allows them to process the state of the game board at a higher level.

Slide 6:

Schema construction applies to physical actions too. In other words, muscle memory.

From Wikipedia (<https://en.wikipedia.org/wiki/Automaticity>): “The ability to do things without occupying the mind with the low-level details”

**[Audience response: what are some examples of automaticity in anesthesia?]**

*Suggested examples: bag mask ventilation, administering IV drugs, direct laryngoscopy*

For example, let’s talk about bag mask ventilation. When you were brand new CA-1s, your brain had to manually consider the position of each finger of your left hand. Now that you are all experienced anesthesia providers, for a typical patient you can simply think, I need to mask ventilate and your left hand positions the mask automatically with minimal thought required. This frees up mental capacity so that instead you can do things like watch for chest rise, check the tidal volumes, look at the end tidal CO2 tracing, etc.

**[Audience response: what are some of the dangers of automaticity when it comes to teaching a new trainee?]**

Often times, the tasks we perform are so automated that we don’t even think about the individual steps. This can make it challenging to transmit the information from you to your trainee. Or we may forget what it was like as a brand-new CA-1 and overwhelm the CA-1 with too much information at once.

Now before we go to the next slide, I want you to turn to your neighbor and work through question #1 on your handout together.

**[Small group session – question #1 – *ask residents to name and explain each of the 3 types of cognitive load (intrinsic load, extraneous load, and germane load). After each explanation, ask for an example related to anesthesia (can call on a different resident if desired)*]**

*Suggested examples:*

*Intrinsic load: placing a central line is intrinsically more cognitively challenging than placing a peripheral IV.*

*Extraneous load: holding a casual conversation with your trainee while they are trying to concentrate on charting.*

*Germane load: this is the cognitive work that your trainee’s brain is doing when they learn how to bag mask.*

Slide 7:

**[Small group session – question #2 – let’s say it is July and you are the one in charge of a brand new CA1. How can you minimize extraneous cognitive load so that they can focus on learning the necessary material?] *(ask residents to work with their partner again. Have them identify each item on the next slide (Minimize distractions, Scaffolding, Worked examples, Integration, and Split-attention effect) and ask for an example in anesthesia. See primer document for detailed explanations and references)***

*Suggested examples:*

*Minimize distractions: Delegate and simplify tasks. For example, if they are learning how to bag mask, tell them to focus on the mask ventilation and that you will take care of the patient’s blood pressure.*

*Scaffolding: Gradually withdraw assistance over time. For example, for morning OR setup during the first few days with your trainee you can help make IV fluids and draw up medications, then over the next several days you stop helping with each of these tasks.*

*Worked examples: Step-by-step breakdown of a task. For example for peripheral IV placement, you can divide the process into identifying landmarks, getting the flash, and threading the catheter. This makes it easier to troubleshoot when something doesn’t go smoothly.*

*Integration: Use multiple modalities in a synergistic fashion. For example chalk talks (actively drawing diagrams/figures on a paper while explaining a concept).*

*Split-attention effect: Avoid competing stimuli. When your trainee is using a MAC 3 blade for the first time, resist to urge to start elaborating about the Miller 2 blade and the relative advantages/disadvantages. Let them get the basic concept down first before moving on.*

Slides 8, 9, and 10. (No additional notes)

Slide 11:

Next we will cover general principles of effective teaching.

Slide 12:

These are the 3 principles that we will discuss: Allow sufficient time, Let them figure it out, and Encourage autonomy.

Slide 13:

As we progress through training, a lot of tasks become second-nature (think muscle memory). This can complicate teaching because something seems obvious and simple, but the trainee is still processing a lot of information and forming mental connections. This takes time. Remember what we talked about earlier with automaticity. Preparing a bag of IV fluids is easy for you, but it is a lot more cognitively challenging for your new CA-1.

Finish one thing at a time before moving on to the next item. For example, if you asked your trainee to give a bolus of medication, let your trainee finish giving the medication before you continue talking again.

Slide 14:

Instead of telling your trainee the answer, use questions and prompts to help guide them to the correct answer. There is a famous quote “Give a man a fish and you feed him for a day; teach a man to fish and you feed him for a lifetime.”

By making your trainee work a little bit for the answer, you will help form more long-lasting mental connections.

Slide 15:

Think for a moment: why does encouraging autonomy lead to better learning?

**[Audience response] (answer: autonomy is a key part of self-determination theory, aka what motivates people to do stuff on their own)**

**[Small group sessions – question #3 – components of self-determination theory] *(ask residents to work together to complete question #3 on the handout)***

Slide 16:

What motivates people to want to learn? One answer is self-determination theory which breaks down into these 3 components: relatedness, competence, autonomy.

Relatedness and competence typically come along naturally from being a resident:

Relatedness: a sense of community – knowing there are other people in the same boat as you. You are part of a group.

Competence: you want to become competent at something.

As a trainer, you can impact all three of these components up here. Most people will naturally satisfy the first two components of relatedness and competence. But we can sometimes accidentally overlook autonomy.

Think back to the last time you were in the operating room and somebody took over a procedure that you had started because you were too slow.

**(Pause to think)**

Think of the last time your attending rejected your plan without a good explanation why (“We’re going to do it this way, no arguments”).

**(Pause to think)**

One of the hardest parts of being a trainer is learning to be hands off. It’s been ingrained in us to help out. To solve this, once your trainee has achieved some basic competence, step back and keep your arms folded. This will help you fight the urge to jump in immediately when something happens. If you must, stand in the corner of the room so that you are far away. Try to let your trainee solve problems on their own if it is safe for the patient (You should still step in if there is something critical happening that can’t wait, but even in those circumstances, when you debrief and discuss afterwards you can ask them what they would have done and why).

Slides 17 and 18. (No additional notes)

Slide 19:

Our last topic during this first hour will be a framework for figuring out if you are delivering high-quality education.

Slide 20:

I would like you all to think back to a time during your medical education (medical school or residency) when you had a great learning experience. Please fill out question #4 and then discuss with your neighbor.

**[Small group session – question #4 – personal experience with learning. Please note that there are 4 parts to the question]**

Would anyone like to share their experiences with the group?

**(Discuss answers to question #4)**

**[Small group session – question #5A – what are the 5 steps to evaluating the quality of teaching]**

*(Residents should work together as a group to answer question #5A and write down the 5 steps)*

Slide 21:

**[Small group session – question #5B - Explain the significance of each step]**

*(Allow residents to discuss question #5B with each other, then proceed through the following 5 slides for each step)*

Slide 22:

Think back to the last time someone yelled at you in the OR. Your learning was probably impaired after the event. For ACLS training nowadays, they emphasize a collaborative environment where people who are lower on the totem pole can still feel safe making suggestions or pointing out errors. This is very similar in teaching. For maximum learning, it is important to provide psychological safety and establish a non-punitive climate. Let your trainee know that it is okay to admit when they don’t know the answer. Validate that it is okay that they don’t know the answer and that this is expected as a learner. Be warm and welcoming. Don’t punish them for mistakes – they are here to learn.

Slide 23:

It is easier for your trainee to follow along if you start out by letting them know what you are about to cover and what you want them to get out of it. For example, “Let’s talk about how to place an arterial line” or “Let’s discuss pressors, specifically phenylephrine and ephedrine”

Slide 24:

Adult learning theory differs from child learning theory. Children have no basis for comparison and accept information a priori (i.e. “This is the color red”). On the other hand, adults learn by relating information back to what they already know. As a teacher, you can promote understanding by **activating prior knowledge**. For example, when talking about atelectasis and lung compliance, ask learners to think about the difficulty of inflating a balloon. The more you can tie a new concept back to something they already know, the better it will stick. Be careful to **avoid information overload**, as discussed above.

Slide 25:

The types of questions that you ask can be important too.

Slide 26:

There are various types of questions you can ask.

**[Small group session – question #6 – types of questions. Ask the audience to explain and provide examples of each type of question (Recall, Synthesis, Application)]**

*Suggested examples:*

***Recall****: “What are the criteria for severe aortic stenosis?”*

***Synthesis****: “What will happen to the left ventricle with longstanding severe aortic stenosis?”*

***Application****: “What are your hemodynamic goals for a patient with severe aortic stenosis?”*

Recall questions are the easiest ones to ask since they are essentially fact regurgitation.

Effective teachers will attempt to use higher-order Synthesis and Application questions to challenge learners to think more deeply and form those mental connections.

Slide 27:

Ensure that there is two-way communication so that you and your trainee remain on the same page. Did that make sense? Am I moving too quickly? Is this too simple?

Slide 28:

**[Small group session – question #7 – microteaching]**

*(Allow residents a few minutes to individually write down a topic for question #7. Ask residents to break into pairs. Each person will practice microteaching for 5 minutes to their partner and then their partner will provide feedback (2 minutes) using the above framework. The preceptor should notify residents when the 5 minutes are up and then ask residents to provide feedback for 2 minutes. Then ask residents to switch roles and repeat.)*

How did that feel?

*(Solicit feedback from residents)*

Slides 29 and 30 (No additional notes)

Slide 31:

*(Offer to take a break before the second hour)*

Slide 32 (No additional notes)

Slide 33:

For this second hour we are going to focus on microskills, otherwise known as the one-minute preceptor. Other frameworks for teaching exist; we have decided to focus on microskills in particular as it has been well-studied with plenty of literature to show its effectiveness in the clinical setting.

Slide 34 (No additional notes)

Slide 35:

Think of this as another tool for your toolbox – it may not be the best way to teach every single topic, but it fills a useful niche.

Slide 36:

There are papers from multiple specialties and disciplines showing that microskills has wide applicability.

Slide 37:

We also have evidence to show that the students learn better and have a better experience when their preceptors use microskills.

Slide 38:

Microskills have been shown to improve resident teaching.

Slide 39 (No additional notes)

Slide 40:

Here are the five steps.

Slide 41:

Let’s use an example as we explain these 5 steps.

Slide 42:

Ask your trainee to commit to a possible explanation. It’s okay if it turns out that they are incorrect. In fact, it works even better if they are incorrect. The purpose of the exercise is to develop their critical thinking skills.

"What do you think is going on with this patient?"

"What do you think the main problem is here?"

"What complaint is the most important to focus on during this visit?"

Slide 43:

Ask your trainee to look for clinical evidence that supports or argues against their hypothesis. Remember, this is still the fact-gathering stage – do not confirm or deny his/her hypothesis yet.

Slide 44:

Explain how you would approach a similar situation. What are the most common explanations for this scenario? What critical issues do you need to rule out?

Slide 45:

Now for the feedback: start by highlighting the things they did well.

Slide 46:

And finally, explain any errors that they made and how to correct them.

Slides 47 through 51 (No additional notes)

End of workshop wrap-up:

Optional: **Appendix F (Post Survey)** can be administered to residents to assess knowledge retention and change in trainer preparedness from the workshop.

1 week post-workshop:

Optional: **Appendix G (1 Week Follow-up Survey)** can be distributed to residents to assess trainer preparedness.

1 month post-workshop:

Optional: **Appendix H (1 Month Follow-up Survey)** can be distributed to residents to assess for knowledge retention.
