# Supplementary material for: Training the Trainer: Preparing Anesthesiology Residents to be Trainers in the Operating Room
Source: MedEdPORTAL. 2021 Mar 4;17:11116. doi: 10.15766/mep_2374-8265.11116 (PMC7970634; doi:10.15766/mep_2374-8265.11116)
Supplement: Supplementary file 1 — Primer Document.docxWorkshop Handout.docxWorkshop PowerPoint.pptxInstructor Manual.docxPresurvey.pdfPostsurvey.pdf1-Week Follow-up Survey.docx1-Month Follow-up Survey.docxNew CA 1 Survey.docx [file mep_2374-8265.11116-s001.zip › F. Postsurvey.pdf]

Posttest

Study ID Number: ________________

1) What is your level of training?

◯ CA-1
◯ CA-2
◯ CA-3

		Strongly Agree	Agree	Neither Agree nor Disagree	Disagree	Strongly Disagree
2)	I am prepared to train a new incoming CA-1 resident in July	◯	◯	◯	◯	◯

3) Please list the 3 types of cognitive load. You may leave this blank or provide a partial response if you are unsure.

4) I am comfortable using Microskills (aka One-Minute Preceptor) to teach someone.

◯ Yes
◯ No

5) Please list the 5 steps of Microskills teaching. You may leave this blank or provide a partial response if you are unsure.

Summary

		Strongly Agree	Agree	Neither Agree nor Disagree	Disagree	Strongly Disagree
6)	This workshop was useful.	◯	◯	◯	◯	◯
7)	This workshop should continue as part of the annual curriculum.	◯	◯	◯	◯	◯

8) Comments/Feedback: (Optional)
