# Supplementary material for: Training the Trainer: Preparing Anesthesiology Residents to be Trainers in the Operating Room
Source: MedEdPORTAL. 2021 Mar 4;17:11116. doi: 10.15766/mep_2374-8265.11116 (PMC7970634; doi:10.15766/mep_2374-8265.11116)
Supplement: Supplementary file 1 — Primer Document.docxWorkshop Handout.docxWorkshop PowerPoint.pptxInstructor Manual.docxPresurvey.pdfPostsurvey.pdf1-Week Follow-up Survey.docx1-Month Follow-up Survey.docxNew CA 1 Survey.docx [file mep_2374-8265.11116-s001.zip › G. 1-Week Follow-up Survey.docx]

1 Week Follow-up Survey

Start of Block: Default Question Block

Q2 Did you attend the workshop on [Insert DATE here] titled “Leverage Learning Theory to Become a Better Trainer”?

- Yes (1)
- No (2)

Display This Question:

If Did you attend the workshop on June 17, 2019 titled “Leverage Learning Theory to Become a Better... = No

Q3 If you did not attend the workshop, have you reviewed the workshop materials on Blackboard?

- Yes (1)
- No (2)

Q1 Year of residency:

- CA-1 (1)
- CA-2 (2)
- CA-3 (3)

Q4 *Please indicate your level of agreement with the following statement:*
I am prepared to train a new incoming CA-1 in July.

- Strongly agree (1)
- Somewhat agree (2)
- Neither agree nor disagree (3)
- Somewhat disagree (4)
- Strongly disagree (5)

End of Block: Default Question Block
