# Supplementary material for: Training the Trainer: Preparing Anesthesiology Residents to be Trainers in the Operating Room
Source: MedEdPORTAL. 2021 Mar 4;17:11116. doi: 10.15766/mep_2374-8265.11116 (PMC7970634; doi:10.15766/mep_2374-8265.11116)
Supplement: Supplementary file 1 — Primer Document.docxWorkshop Handout.docxWorkshop PowerPoint.pptxInstructor Manual.docxPresurvey.pdfPostsurvey.pdf1-Week Follow-up Survey.docx1-Month Follow-up Survey.docxNew CA 1 Survey.docx [file mep_2374-8265.11116-s001.zip › H. 1-Month Follow-up Survey.docx]

1 Month Follow-up Survey

Start of Block: Default Question Block

Q1 Did you attend the workshop (Leveraging Learning Theory to Become a Better Trainer), or read the workshop materials if you were unable to attend?

- Yes (23)
- No (24)

Q2 Regarding the Training-In period:

|  | Strongly Agree (1) | Agree (2) | Neither Agree nor Disagree (3) | Disagree (4) | Strongly Disagree (5) |
| --- | --- | --- | --- | --- | --- |
| I was prepared to train my CA-1 resident. (1) |  |  |  |  |  |
| I provided sufficient autonomy to my CA-1 resident. (2) |  |  |  |  |  |
| I covered all of the essential material. (3) |  |  |  |  |  |

Q7 Without looking it up, please list the 3 types of cognitive load. You may leave this blank or provide a partial response if you are unsure.

________________________________________________________________

Q3 I am comfortable using Microskills (aka One-Minute Preceptor) to teach someone.

- Strongly Agree (1)
- Agree (2)
- Neither Agree nor Disagree (3)
- Disagree (4)
- Strongly Disagree (5)

Q4 Without looking it up, please list the 5 steps of Microskills teaching. You may leave this blank or provide a partial response if you are unsure.

________________________________________________________________

Q5 Comments/Feedback (optional)

________________________________________________________________

End of Block: Default Question Block
