# Supplementary material for: Training the Trainer: Preparing Anesthesiology Residents to be Trainers in the Operating Room
Source: MedEdPORTAL. 2021 Mar 4;17:11116. doi: 10.15766/mep_2374-8265.11116 (PMC7970634; doi:10.15766/mep_2374-8265.11116)
Supplement: Supplementary file 1 — Primer Document.docxWorkshop Handout.docxWorkshop PowerPoint.pptxInstructor Manual.docxPresurvey.pdfPostsurvey.pdf1-Week Follow-up Survey.docx1-Month Follow-up Survey.docxNew CA 1 Survey.docx [file mep_2374-8265.11116-s001.zip › I. New CA 1 Survey.docx]

New CA1s Survey

Start of Block: Default Question Block

Q1 Regarding the Training-In period:

|  | Strongly Agree (1) | Agree (2) | Neither Agree nor Disagree (3) | Disagree (4) | Strongly Disagree (5) |
| --- | --- | --- | --- | --- | --- |
| My trainer was prepared to train me. (1) |  |  |  |  |  |
| My trainer allowed me sufficient autonomy. (2) |  |  |  |  |  |
| My trainer covered all of the essential material. (3) |  |  |  |  |  |

Q2 Comments/Feedback (optional)

________________________________________________________________

End of Block: Default Question Block
