# Supplementary material for: Training the Trainer: Preparing Anesthesiology Residents to be Trainers in the Operating Room
Source: MedEdPORTAL. 2021 Mar 4;17:11116. doi: 10.15766/mep_2374-8265.11116 (PMC7970634; doi:10.15766/mep_2374-8265.11116)
Supplement: Supplementary file 1 — Primer Document.docxWorkshop Handout.docxWorkshop PowerPoint.pptxInstructor Manual.docxPresurvey.pdfPostsurvey.pdf1-Week Follow-up Survey.docx1-Month Follow-up Survey.docxNew CA 1 Survey.docx [file mep_2374-8265.11116-s001.zip › _Educational_Summary_Report_11116.pdf]

MedEdPORTAL® The AAMC Journal of Teaching and Learning Resources

Original Publication Open Access

Training the Trainer: Preparing Anesthesiology Residents to be Trainers in the Operating Room

Jeffrey Huang, MD*, Lauren Licatino, MD, Charles R. Sims III, MD

*Corresponding author: huang.jeffrey@mayo.edu

Abstract

Introduction: The transition into clinical anesthesiology is a challenging period that requires swift acquisition of clinical knowledge and procedural skills. Senior residents are in a prime position to help their junior colleagues into the operating room environment due to their ability to relate from personal experience. We created a workshop for enhancing peer apprenticeship during this transition. **Methods:** The workshop consisted of PowerPoint didactics interspersed with small-group practice sessions. Surveys were administered pre-, post-, 1-week post-, and 1-month postworkshop. The primary outcome was pre-post improvement in the proportion of residents prepared to be a trainer. Secondary outcomes included pre- to 1-week postworkshop improvement, pre-postworkshop change in knowledge of learning theory concepts, and pre-postworkshop change in first-year clinical anesthesiology perceptions of trainers. **Results:** Of residents, 12 of 43 (28%) eligible to be resident trainers attended the workshop. The proportion of residents who felt prepared increased from 75% preworkshop to 100% postworkshop and remained at 93% at 1 week. Knowledge of cognitive load and microskills improved from 0% preworkshop to 83% postworkshop but dropped to 0% at 1 month. Comfort using microskills improved from 0% preworkshop to 83% postworkshop. **Discussion:** Early anesthesiology training demands rapid acquisition of novel cognitive and procedural skills. Senior anesthesiology residents are in a prime position to train junior residents, yet many are uncomfortable with this role. We developed a workshop to transition residents into a peer trainer role and significantly increased their confidence to be a trainer. Other programs may benefit from implementing similar training.

Keywords
Orientation, Peer Tutoring, Microskills, Cognitive Load Theory, Anesthesiology, Clinical/Procedural Skills Training

Educational Objectives

By the end of this activity, learners will be able to:

1. Orient new anesthesiology residents to the operating room environment over the course of 3 weeks.
2. Understand how to decrease and increase cognitive load appropriately for their trainee.
3. Use microskills to enhance intraoperative teaching.

Introduction

While the benefits of resident-as-teacher programs are well described for many specialties, anesthesiology remains underrepresented in this area.[1-4] Prior anesthesiology literature has focused on preparing residents to supervise a wide range of other health care professionals.[5] We believe that this workshop represents a unique contribution to the literature by specifically focusing on the transition from intern year into the operating room environment.

Citation:
Huang J, Licatino L, Sims CR III. Training the trainer: preparing anesthesiology residents to be trainers in the operating room. *MedEdPORTAL*. 2021;17:11116.
https://doi.org/10.15766/mep_2374-8265.11116

Anesthesiology uniquely challenges new residents, who are introduced to a high-pressure environment where mistakes and oversights can have immediate and life-threatening adverse consequences for patients. Knowledge retention and future memory retrieval are known to be impaired under stressful circumstances, and stress biomarkers have been shown to be elevated during this transition period.[6,7] Yet within the span of a few weeks, anesthesiology residents are expected to rapidly acquire basic competence in both the clinical knowledge and procedural skills necessary to function as a perioperative care provider.

Prior research has shown that a majority of United States anesthesiology residency programs pair new residents 1:1

Copyright © 2021 Huang et al. This is an open-access publication distributed under the terms of the Creative Commons Attribution license.

with a senior resident (70%) and/or a faculty member (75%).[8] Similar to other programs around the country, the Mayo Clinic's anesthesiology residency program in Rochester, Minnesota employs direct observational learning during an orientation period in the first 3 weeks of first-year clinical anesthesiology (CA 1), where each new trainee is paired with a senior resident. The Mayo Clinic anesthesiology residency program begins the academic year on July 1 each year. All CA 1 residents undergo a 4-week orientation period starting July 1. Anesthesia care at our institution is provided under a care team model. Each CA 1 resident works with many different supervising anesthesiologists over this 3-week period but spends the majority of their clinical time consistently paired with a specific assigned senior resident. The senior resident is usually a second- (CA 2; PGY 3) or third-year (CA 3; PGY 4) clinical anesthesiology trainee, but occasionally residents who have extended their training beyond CA 3 may participate as peer trainers.

As is the case at most anesthesiology residency programs in the United States,[8] senior residents do not receive any formal teaching preparation or introduction to learning theory prior to these interactions. We have solicited feedback from senior residents in the past and found that a significant portion of residents felt underprepared to be trainers and desired a formal curriculum. A preliminary survey of 56 anesthesiology residents with a response rate of 34 (61%) found that 23 of the 34 respondents (68%) felt prepared to train a new CA 1 resident during the upcoming academic year. Our goal was to improve this proportion to 90%.

Goodlad and Hirst define peer tutoring as, "The system of instruction in which learners help each other and learn by teaching."[9] Undergraduate medical education literature has demonstrated multiple benefits of peer teaching, including greater motivation in students and teachers, role modeling for students, promoting a safe learning environment, and preparing trainees to be future educators.[10-13] We believe that many of these same benefits carry over at the resident level. Specifically, we applied this process to the opening months of clinical anesthesiology training, where cognitive and procedural learning curves are steep. Because senior residents are expected to both tutor on clinical concepts and coach their junior resident through procedures, we believe that the term "peer apprenticeship" best describes the relationship between the senior resident trainer and junior resident apprentice during this critical period. The target audience of this module is educational leaders in anesthesiology residency programs. We aimed to provide a template that can be implemented at other institutions to prepare their senior anesthesiology residents as trainers.

We selected a workshop format enhanced by digital content due to the benefit of in-person interactions in describing and evaluating essential skills while balancing resident scheduling constraints. Our primary objective was to improve residents' confidence in their ability to be a trainer. In this instance, training confidence was defined by the level at which a senior resident (CA 2 or CA 3) felt prepared to transmit essential knowledge and skills required by a junior resident (CA 1) during the transition into the operating room environment. We secondarily worked to have senior residents: 1) enhance their ability to identify and manage learner cognitive load, a key topic due to the vast amount of material to cover in a short period of time and its effects on knowledge acquisition and retention;[14-19] 2) apply elements of self-determination theory and Bloom's taxonomy which are useful frameworks for resident trainers;[20,21] and 3) incorporate microskills into their teaching, due to the volume of evidence supporting its use across multiple clinical contexts and specialties.[22-26]

Methods

After institutional review board exemption (ID 19-008959), we developed a workshop to prepare senior residents for the July 2019 orientation period. The authors gathered input from the residency program educational leadership on the list of workshop topics then author Jeffrey Huang, MD created the workshop materials with feedback from the other authors and educational leadership. We scheduled this workshop as a 2-hour core didactic session in June 2019 in an audio/video-equipped classroom. All residents who were eligible to be resident trainers during the upcoming July 2019 orientation period were invited to attend. The intended audience of this workshop is the senior resident learner and those who wish to enhance intraoperative peer apprenticeship. Facilitators of this workshop should be physician-educators within the practice of anesthesiology. We recommend that at least one facilitator have completed residency training within the past 5 years or be near the end of their residency training. We recommend conducting the workshop 1 month prior to CA 1 resident orientation.

One week prior to the workshop, we distributed a primer document (Appendix A) through our learning management system (Blackboard Collaborate) and asked residents to read the material before attending the workshop. At the start of the workshop, we distributed a handout (Appendix B) to all attendees. We asked residents to seat themselves at tables arranged in horizontal rows facing the projector screen at the front of the room. Over the course of 2 hours, we interspersed lecture-based didactics using a PowerPoint presentation

Copyright © 2021 Huang et al. This is an open-access publication distributed under the terms of the Creative Commons Attribution license.

(Appendix C) and interactive small-group sessions. We have included an instructor manual containing the PowerPoint slide notes as Appendix D. We introduced each topic using PowerPoint slides and then reinforced with hands-on practice as residents worked together in groups of two to three to complete the corresponding portion of the handout. We have noted the transitions between large- and small-group sessions in the speaker notes for the PowerPoint slides. During the first hour of the workshop, we focused on cognitive load theory, principles of effective teaching, and methods of evaluating the quality of teaching. During the second hour we focused on microskills.

We assessed residents' knowledge and confidence with printed copies of the pre- and postsurveys before and after the workshop (Appendices E and F). We created follow-up surveys in Qualtrics software and distributed the surveys electronically through email 1 week (Appendix G) and 1 month (Appendix H) after the workshop to all 43 residents who were potentially eligible to be trainers. We also distributed a survey (Appendix I) to new CA 1 residents at the end of their orientation month to assess their perceptions of their senior resident trainers. These surveys were designed and revised based on feedback from members of departmental educational leadership and all responses were collected anonymously.

Our primary outcome was improvement in the proportion of residents who felt prepared to be trainers, from pre- to postworkshop. We used a 5-point Likert response to the statement, "I am prepared to train in a new CA 1 resident in July." We counted responses as feeling prepared if a resident selected either *agree* or *strongly agree*. As a secondary outcome, we assessed the same statement from pre- to 1-week postworkshop. We also included the following secondary outcomes: learner reactions to the workshop; change in knowledge pre- to postworkshop regarding the three types of cognitive load and the five steps of microskills; and retention of cognitive load and microskills knowledge at 1-month postworkshop. As a counterbalance measure, we also included changes in CA 1 perceptions of their trainers from past orientation periods (preworkshop) to postworkshop orientation.

Results

A total of 14 residents attended the workshop. Twelve residents (six CA 1s, five CA 2s, and one CA 3) submitted their pre- and postworkshop surveys representing 28% of the 43 residents who could potentially be selected as trainers for the upcoming new CA 1 orientation. The remaining two resident participants were CA 3 residents who were not eligible to be trainers in July due to their imminent graduation and departure.

Reactions

At preworkshop, nine of the 12 residents (75%) agreed that they were prepared to train a new incoming CA 1 resident in July. Postworkshop, this increased to all 12 residents (100%) agreeing with the above statement. Ten of 12 residents (83%) felt that the workshop was useful.

Learning

At preworkshop, none of the 12 residents (0%) were able to correctly list the three types of cognitive load or the five steps of microskills, and none (0%) felt comfortable using microskills. Postworkshop, 10 of 12 residents (83%) were able to correctly list the three types of cognitive load, 10 of 12 residents (83%) were able to correctly list the five steps of microskills, and 10 of 12 residents (83%) felt comfortable using microskills.

Apprentice Perception of Their Trainer

At preworkshop, 11 of the 12 senior residents (92%) felt that their own trainer in the past had been prepared, 12 of 12 residents (100%) felt that their trainer had provided appropriate autonomy, and 10 of 12 residents (83%) felt that their trainer had covered all of the essential material. After the workshop, at the end of July 2019, the new CA 1 class reported that 17 of 17 residents (100%) felt that their trainer was prepared, 16 of 17 residents (94%) felt that their trainer provided appropriate autonomy, and 16 of 17 residents (94%) felt that their trainers covered all of the essential material.

Overall Impact

In order to assess the overall impact of the workshop on all residents, we distributed the 1-week and 1-month postworkshop surveys to the 43 residents (excluding the first author) who could potentially be selected as trainers (19 CA 1s, 18 CA 2s, and six CA 3s) including residents who were unable to attend the workshop but were provided with the digital workshop materials as self-directed learning. The response rate for these surveys was 14 (33%) at 1-week post and seven (16%) at 1-month post. Out of those who responded to the 1-week postsurvey, 13 of 14 (93%) felt prepared to train a new incoming CA 1 resident in July. Nine respondents had attended the workshop. Of the five respondents who did not attend, four had reviewed the digital workshop materials. The one respondent who did not feel prepared was the only one who neither attended the workshop nor reviewed the digital workshop materials. At the 1-month postsurvey, none of the seven respondents were able to correctly list all three types of cognitive load or all five steps of microskills.

Copyright © 2021 Huang et al. This is an open-access publication distributed under the terms of the Creative Commons Attribution license.

Discussion

The transition into clinical anesthesiology is a stressful and challenging period. There is high potential for information overload due to multiple, unfamiliar procedural skills as well as clinical knowledge that must be acquired in a short period of time. Among the many benefits of peer teaching are the shared knowledge base and the ability to create a comfortable and safe learning environment.[10-13] As such, senior residents are in a prime position to help their junior colleagues. Adequate preparation of the senior residents may help new learners mitigate the stress of this transition and promote optimal learning and patient safety. This study found that at baseline senior residents were not familiar with formal learning theory or cognitive load, and that overall CA 1 resident perceptions of their trainers was favorable and not negatively impacted by our intervention.

Lessons Learned and Future Directions

Our workshop successfully improved both senior resident confidence and knowledge of teaching principles. Familiarity with and ability to apply microskills also improved. All 12 residents who participated in the workshop and were eligible to train incoming CA 1 residents felt prepared to be a trainer by the end of the workshop. The 1-week postsurvey was sent to workshop attendees, as well as nonattendees since they had access to the workshop digital material. While the 1-week response rate was low overall, a large proportion of attendees who responded felt prepared at 1 week out from the workshop. Four of the nonattendee respondents indicated that although they were unable to attend the workshop, they had reviewed the digital workshop content. These results highlighted the importance of distributing the workshop content digitally to reach residents who were unable to physically attend the workshop. We utilized a learning management system to facilitate content distribution. Email distribution is a reasonable alternative for programs that have not yet implemented a learning management system platform. The in-person component of the workshop can be readily adapted for digital delivery in the setting of virtual learning by holding the workshop through remote conferencing software. In such a scenario, the small-group sessions may be replaced with audience polling either through the conferencing software or through a separate online audience response system.

An important observation from our study is that content reinforcement needs to continue well beyond the initial classroom session. Senior trainees failed to recall many of the details of the workshop experience 1 month after the event. Future work could assess retention rates at 6 months and 1 year, with variable review strategies to determine an appropriate reinforcement timeline. As we iteratively evaluate our workshop, we have considered adding additional scenarios and incorporating in situ simulation. For example, participants may role-play using brief scripts and then critique the training. Prerecorded videos of teaching moments could also be similarly critiqued on items that went well and items that could be improved. We plan to repeat the workshop on an annual basis and lecture recordings will help further improve our ability to reach residents who are unable to attend in person. We would also like to introduce elements of gamification, which has been shown to enhance learning and could help further improve the peer apprenticeship process by encouraging friendly competition among senior and junior residents.[27]

Limitations

Our sample size was limited by the nature of residency schedules, with some interested residents unable to participate due to clinical duties and other obligations. Improvements in availability of residents could be accomplished by live-streaming the workshop to allow more flexibility in attendance or providing multiple sessions across a period of time to catch the residents who were unable to attend the first time. Other limitations included a low response rate on the survey instruments and that the primary outcome was a subjective measure, which may not truly reflect senior resident preparation to be a trainer. The subjective assessments of trainers by their new CA 1 trainees were also limited in that the new CA 1 residents did not have a reference point and may thus have overrated their trainer's preparedness. This could be improved by asking CA 1 residents to evaluate their trainer again at some point later on in their training. We did include objective outcomes in the form of knowledge acquisition by trainers. A more objective assessment of preparation would be a formalized exam of senior residents after the workshop. Of note, we did not follow a formal survey validation process in designing the survey instruments used in this workshop.

The questions about past orientation experience as a CA 1 may have been affected by recall bias, as CA 2 and CA 3 residents (pregroup) were multiple years distant from their CA 1 orientation experience, whereas the new CA 1 residents (postgroup) had just completed their orientation. However, residents consistently reported positive opinions of their trainers in prior years as well as after the workshop.

Knowledge retention was poor at 1 month after the workshop. Very few residents responded to the 1-month survey, though we suspect that the outcome would have remained similar even with

Copyright © 2021 Huang et al. This is an open-access publication distributed under the terms of the Creative Commons Attribution license.

a higher response rate. The format of these follow-up questions (i.e., recall rather than recognition) may also explain this apparent rapid decay of information by 1 month. This assessment could be improved by asking residents to identify specific trainer behaviors or having a trained observer present while residents are teaching. The workshop would also likely be more effective as a longitudinal curriculum, such as a scheduled monthly resident development session covering essential topics beyond learning theory.

We included in our study population residents in their last month of CA 1 year. We recognize that there are certain downsides to including CA 1 residents as trainers who may themselves be in the process of transitioning and could benefit from further apprenticeship. It may be helpful to have an attending anesthesiologist screen potential resident trainers with a checklist to ensure that residents meet designated performance measures prior to being selected as a trainer.

Conclusion

The successful ability of senior residents to transfer knowledge and skill to junior residents is a vital part of residency. Our senior residents felt insufficiently prepared to train their new junior colleagues. We successfully addressed this deficiency by developing and implementing a workshop on evidence-based learning theory. Incorporating a training-the-trainer curriculum may improve training confidence and learner retention among senior residents at other institutions. However, additional higher-powered studies are needed.

Appendices

A. Primer Document.docx
B. Workshop Handout.docx
C. Workshop PowerPoint.pptx
D. Instructor Manual.docx
E. Presurvey.pdf
F. Postsurvey.pdf
G. 1-Week Follow-up Survey.docx
H. 1-Month Follow-up Survey.docx
I. New CA 1 Survey.docx

All appendices are peer reviewed as integral parts of the Original Publication.

Jeffrey Huang, MD: Fellow, Department of Critical Care, Mayo Clinic Rochester; ORCID: https://orcid.org/0000-0001-5574-6756

Lauren Licatino, MD: Assistant Professor, Department of Anesthesiology and Perioperative Medicine, Mayo Clinic Rochester

Charles R. Sims III, MD: Assistant Professor, Department of Anesthesiology and Perioperative Medicine, Mayo Clinic Rochester; Assistant Professor, Department of Critical Care, Mayo Clinic Rochester

Acknowledgments
We would like to acknowledge the Harvard Macy Institute's Program for Postgraduate Trainees: Future Academic Clinician-Educators for providing the background on evaluating the quality of teaching, adapted from Stanford University. We would like to acknowledge Jonathan Charnin, MD, for his guidance on cognitive load theory.

Disclosures
None to report.

Funding/Support
None to report.

Ethical Approval
The Mayo Clinic Institutional Review Board approved this study.

References

1. Frey-Vogel A. A resident-as-teacher curriculum for senior residents leading morning report: a learner-centered approach through targeted faculty mentoring. *MedEdPORTAL*. 2020;16:10954. https://doi.org/10.15766/mep_2374-8265.10954
2. Caton JB, Penn EH, Nemer MK, Katz JT, Yialamas MA. Getting up to speed: a resident-led inpatient curriculum for new internal medicine interns. *MedEdPORTAL*. 2019;15:10866. https://doi.org/10.15766/mep_2374-8265.10866
3. Farrell SE, Pacella C, Egan D, et al. Resident-as-teacher: a suggested curriculum for emergency medicine. *Acad Emerg Med*. 2006;13(6):677-679. https://doi.org/10.1197/j.aem.2005.12.014
4. Geary A, Hess DT, Pernar LIM. Resident-as-teacher programs in general surgery residency—a review of published curricula. *Am J Surg*. 2019;217(2):209-213. https://doi.org/10.1016/j.amjsurg.2018.09.003
5. Berger JS, Daneshpayeh N, Sherman M, et al. Anesthesiology residents-as-teachers program: a pilot study. *J Grad Med Educ*. 2012;4(4):525-528. https://doi.org/10.4300/JGME-D-11-00300.1
6. Vogel S, Schwabe L. Learning and memory under stress: implications for the classroom. *NPJ Science Learn*. 2016;1:16011. https://doi.org/10.1038/npjscilearn.2016.11
7. Eisenach JH, Sprung J, Clark MM, et al. The psychological and physiological effects of acute occupational stress in new anesthesiology residents: a pilot trial. *Anesthesiology*. 2014;121(4):878-893. https://doi.org/10.1097/ALN.0000000000000397

Copyright © 2021 Huang et al. This is an open-access publication distributed under the terms of the Creative Commons Attribution license.

8. Huang J, Licatino LK, Long TR. Methods of orienting new anesthesiology residents to the operating room environment: a national survey of residency program directors. *J Educ Perioper Med*. 2020;22(3):E645. https://doi.org/10.46374/volxxii-issue3-Licatino

9. Goodlad S, Hirst B. *Peer Tutoring. A Guide to Learning by Teaching*. Nichols Publishing; 1989.

10. Tucker C, Gibbs K. The medical master tutor training. *MedEdPORTAL*. 2012;8:9116. https://doi.org/10.15766/mep_2374-8265.9116

11. Bell M, Defilippo C, Miloslavsky E, et al. Medical school peer-tutoring training curricula. *MedEdPORTAL*. 2010;6:7810. https://doi.org/10.15766/mep_2374-8265.7810

12. Meredith S, Greenberg L, Blatt B. Resource to develop medical students into peer mentors. *MedEdPORTAL*. 2014;10:9691. https://doi.org/10.15766/mep_2374-8265.9691

13. Cate OT, Durning S. Peer teaching in medical education: twelve reasons to move from theory to practice. *Med Teach*. 2007;29(6): 591-599. https://doi.org/10.1080/01421590701606799

14. Miller GA. The magical number seven, plus or minus two: some limits on our capacity for processing information. *Psychol Rev*. 1956;63(2):81-97. https://doi.org/10.1037/h0043158

15. Chase WG, Simon HA. Perception in chess. *Cognitive Psychol*. 1973;4(1):55-81. https://doi.org/10.1016/0010-0285(73)90004-2

16. Clark RC, Nguyen F, Sweller J. *Efficiency in Learning: Evidence-Based Guidelines to Manage Cognitive Load*. Pfeiffer; 2006. https://doi.org/10.1002/pfi.4930450920

17. Mayer RE, Moreno R. Nine ways to reduce cognitive load in multimedia learning. *Educ Psychol*. 2003;38(1):43-52. https://doi.org/10.1207/S15326985EP3801_6

18. Sweller J. The worked example effect and human cognition. *Learn Instruc*. 2006;16(2):165-169. https://doi.org/10.1016/j.learninstruc.2006.02.005

19. Chandler P, Sweller J. The split-attention effect as a factor in the design of instruction. *Educ Psychol*. 1992;62(2):233-246. https://doi.org/10.1111/j.2044-8279.1992.tb01017.x

20. Ryan RM, Deci EL. Self-determination theory and the facilitation of intrinsic motivation, social development, and well-being. *Am Psychol*. 2000;55(1):68-78. https://doi.org/10.1037/0003-066X.55.1.68

21. Bloom BS, Englehart MD, Furst E, Hill WH, Krathwohl DR. *Taxonomy of Educational Objectives: The Classification of Educational Goals. Handbook I: Cognitive Domain*. David McKay Company; 1956.

22. Neher JO, Gordon KC, Meyer B, Stevens N. A five-step "microskills" model of clinical teaching. *J Am Board Fam Pract*. 1992;5:419-24.

23. Neher JO, Stevens NG. The one-minute preceptor: shaping the teaching conversation. *Fam Med*. 2003;35(6):391-393.

24. Farrell SE, Hopson LR, Wolff M, Hemphill RR, Santen SA. What's the evidence: a review of the one-minute preceptor model of clinical teaching and implications for teaching in the emergency department. *J Emerg Med*. 2016;51(3):278-83. https://doi.org/10.1016/j.jemermed.2016.05.007

25. Teherani A, O'Sullivan P, Aagaard EM, Morrison EH, Irby DM. Student perceptions of the one-minute preceptor and traditional preceptor models. *Med Teach*. 2007;29(4):323-327. https://doi.org/10.1080/01421590701287988

26. Furney SL, Orsini AN, Orsetti KE, Stern DT, Gruppen LD, Irby DM. Teaching the one-minute preceptor: a randomized controlled trial. *J Gen Intern Med*. 2001;16:620-624. https://doi.org/10.1046/j.1525-1497.2001.016009620.x

27. Chon DYK. Benefits and challenges with gamified multimedia physiotherapy case studies: a mixed method study. *Arch Physiotherapy*. 2019;9(7). https://doi.org/10.1186/s40945-019-0059-2

Received: April 24, 2020
Accepted: December 24, 2020
Published: March 4, 2021

Copyright © 2021 Huang et al. This is an open-access publication distributed under the terms of the Creative Commons Attribution license.
